# Effect of Nutrient Solution Flow Rate on Hydroponic Plant Growth and Root Morphology

**DOI:** 10.3390/plants10091840

**Published:** 2021-09-05

**Authors:** Bateer Baiyin, Kotaro Tagawa, Mina Yamada, Xinyan Wang, Satoshi Yamada, Yang Shao, Ping An, Sadahiro Yamamoto, Yasuomi Ibaraki

**Affiliations:** 1 United Graduate School of Agricultural Sciences, Tottori University, Tottori 680-8550, Japan; d19a3004z@edu.tottori-u.ac.jp; 2 Faculty of Agriculture, Tottori University, Tottori 680-8553, Japan; myamada.mimosa@gmail.com (M.Y.); syamada@tottori-u.ac.jp (S.Y.); yamasada@tottori-u.ac.jp (S.Y.); 3 Graduate School of Sustainability Science, Tottori University, Tottori 680-8550, Japan; wangxinyan.tottori@gmail.com; 4 Arid Land Research Center, Tottori University, Tottori 680-0001, Japan; D16A4001Z@edu.tottori-u.ac.jp (Y.S.); an.ping@alrc.tottori-u.ac.jp (P.A.); 5 Faculty of Agriculture, Yamaguchi University, Yamaguchi 753-8515, Japan; ibaraki@yamaguchi-u.ac.jp

**Keywords:** hydroponics, nutrient uptake, plant growth, root morphology, LED, flow rate, eustress, mechanical stimulation, thigmomorphogenesis, dryland agriculture

## Abstract

Crop production under hydroponic environments has many advantages, yet the effects of solution flow rate on plant growth remain unclear. We conducted a hydroponic cultivation study using different flow rates under light-emitting diode lighting to investigate plant growth, nutrient uptake, and root morphology under different flow rates. Swiss chard plants were grown hydroponically under four nutrient solution flow rates (2 L/min, 4 L/min, 6 L/min, and 8 L/min). After 21 days, harvested plants were analyzed for root and shoot fresh weight, root and shoot dry weight, root morphology, and root cellulose and hemicellulose content. We found that suitable flow rates, acting as a eustress, gave the roots appropriate mechanical stimulation to promote root growth, absorb more nutrients, and increase overall plant growth. Conversely, excess flow rates acted as a distress that caused the roots to become compact and inhibited root surface area and root growth. Excess flow rate thereby resulted in a lower root surface area that translated to reduced nutrient ion absorption and poorer plant growth compared with plans cultured under a suitable flow rate. Our results indicate that regulating flow rate can regulate plant thigmomorphogenesis and nutrient uptake, ultimately affecting hydroponic crop quality.

## 1. Introduction

Increased water shortage and land degradation pose challenges to dryland conventional agriculture [1]. In recent years, the development of light-emitting diode (LED) technology has enabled low-cost indoor cultivation using artificial light [2] and soilless culture [3] to enable plant production in places that are unsuitable for crop growth.

Hydroponics is a water-saving cultivation method [4] that is often used in dryland farming. Compared with traditional soil culture and substrate culture, hydroponics uses different culture media (nutrient solution) and incorporates strict environmental control technologies to regulate pH, electrical conductivity (EC), and temperature [5]. Due to its flowable culture media, hydroponics also displays different nutrient migration patterns than does traditional soil culture.

Plant nutrients in the soil typically reach the root surface by root extension, mass flow, and diffusion [6]. In hydroponic cultivation, flowable nutrient solution cultivation substrates also allow nutrients to be transported to the root surfaces via turbulent diffusion [7], which transfers nutrient ions to the root surface by the irregular motion of fluid particles. Turbulent diffusion is affected by a container’s flow rate, which determines the circulation and diffusion of nutrient ions in the container and the resulting nutrient uptake and plant growth.

Recently, various researchers have investigated the effect of nutrient solution flow rate on crop growth. Dalastra et al. [8] evaluated hydroponic lettuce nutrition and production based on nutrient solution flow rates of 0.5, 1, 2, and 4 L/min applied to separate cultivation channels. The greatest nutrient accumulation was found in the plant shoots, with the highest lettuce yield obtained at a nutrient solution flow rate of 1 L/min. Soares et al. [9] evaluated two nutrient solution flow rates (1.5 and 2.5 L/min) using brackish water in hydroponic vegetable production, with the flow rate of 1.5 L/min showing the best results in shoot fresh weight, shoot dry weight, leaf area, number of leaves, plant height, and shoot diameter. Genuncio et al. [10] studied the fresh weights of three different lettuce varieties (Lucy, Izabela, and Veneza) grown at three different flow rates (0.75, 1, and 1.5 L/min) and nutrient ion concentrations (the 50%, 75%, and 100% concentration of the standard nutrient solution in that study). Nutrient solution with a flow rate of 1.5 L/min and 100% ionic concentration increased the fresh weight of Izabela and Veneza lettuce varieties. Finally, Al-Tawaha et al. [11] investigated the effect of three different nutrient solution flow rates (10, 20, and 30 L/min) on lettuce growth, finding that the lettuce weight increased most under a 20 L/min flow rate. The results of these studies indicate that flow rate affects hydroponic plant growth, and that regulating flow rate is recommended to increase hydroponic vegetable yields.

The influence of nutrient solution flow rate on plant growth is related to the plants’ physical environment. The flow of nutrient solution not only promotes nutrient ion diffusion, but also increases the kinetic energy (dynamic pressure) available to plant roots. Previous researchers have demonstrated that nutrient solution flow rate affects hydroponic plant growth; however, the mechanism of this phenomenon has not been explained in detail. In particular, the response of hydroponic plant roots to physical stimulation by water flow has not yet been described in detail. The regulation of flow rate in hydroponic production affects plant growth, which in turn affects crop yield and quality. Therefore, it is necessary to explain the effect of flow rate on plant growth to better regulate hydroponic crop quality.

We performed a hydroponic cultivation experiment under artificial lighting to investigate how plants respond to different nutrient solution flow rates. To do this, we evaluated the effect of different nutrient solution flow rates on plant growth, nutrient uptake, root morphology, and cell wall composition.

## 2. Results

### 2.1. Effect of Flow Rate on Plant Growth and Nutrient Uptake

**Figure 1:**
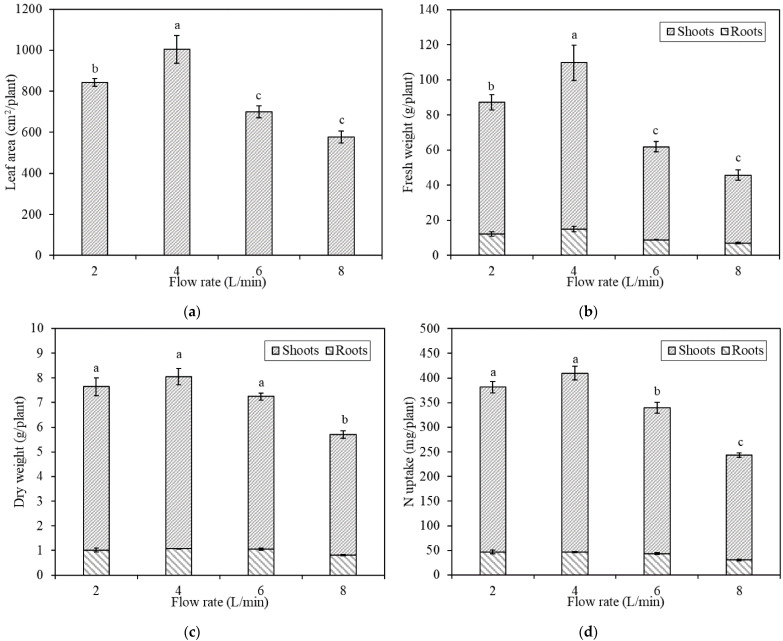
Plant growth and nutrient uptake under different flow rates as measured by (**a**) leaf area; (**b**) fresh weight; (**c**) dry weight; and (**d**) N uptake. Bars labeled with different letters differ significantly (*p* < 0.05). Data are expressed as means ± standard error (*n* = 4).

Plant leaf area increased by 19.0% when the flow rate increased from 2 L/min to 4 L/min. However, the leaf area decreased by 30.3% when the flow rate increased from 4 L/min to 6 L/min, and by 42.5% when the flow rate increased from 4 L/min to 8 L/min (Figure 1a).

We found that plant fresh weight increased by 26.0% when the flow rate increased from 2 L/min to 4 L/min. However, the plant fresh weight decreased by 43.6% when the flow rate increased from 4 L/min to 6 L/min, and by 58.3% when the flow rate increased from 4 L/min to 8 L/min (Figure 1b).

The mean plant dry weight increased when the flow rate increased from 2 L/min to 4 L/min and decreased when the flow rate increased from 4 L/min to 8 L/min (Figure 1c). The mean dry weight showed a significant decrease at 8 L/min compared with the other flow rates.

The plant nitrogen (N) uptake increased by 7.5% when the flow rate increased from 2 L/min to 4 L/min. Conversely, the N uptake decreased by 17.1% when the flow rate increased from 4 L/min to 6 L/min, and by 40.5% when the flow rate increased from 4 L/min to 8 L/min (Figure 1d).

### 2.2. Effect of Flow Rate on Root Morphology, Cellulose, and Hemicellulose

Nutrient solution flow rate affects plant roots before other plant organs. Therefore, to explain the impact of flow rate on plant growth, we must explore the impact of flow rate on roots and the response of roots to the stimulation of flow rate.

**Figure 2:**
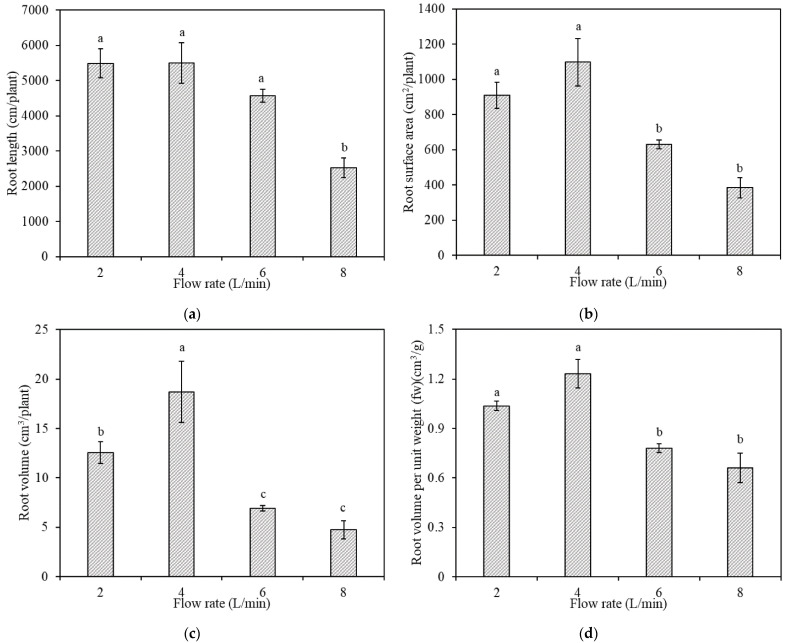

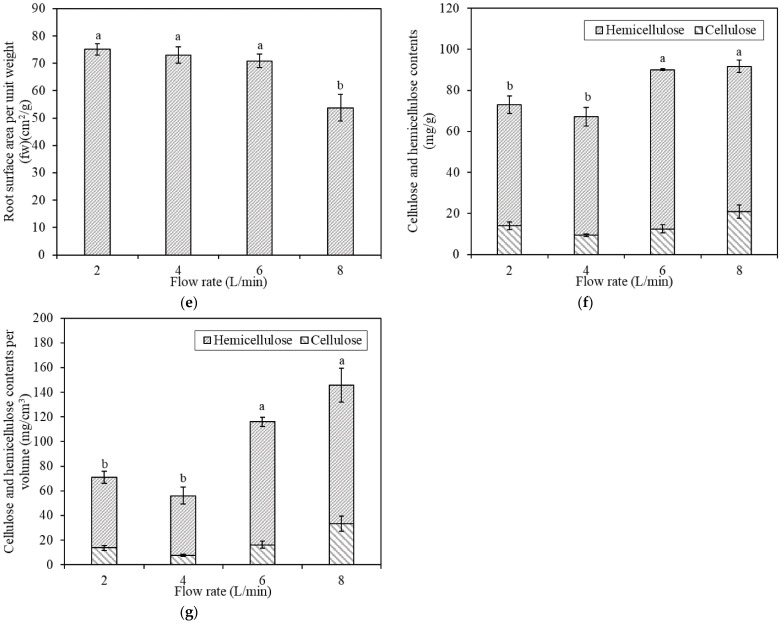
Morphology and cell wall composition of roots under different flow rates, as measured by (**a**) root length; (**b**) root surface area; (**c**) root volume; (**d**) root volume per unit weight; (**e**) root surface per unit weight; (**f**) cellulose and hemicellulose contents per unit fresh weight; and (**g**) cellulose and hemicellulose contents per unit volume. Bars labeled with different letters differ significantly (*p* < 0.05). Data are expressed as means ± standard error (*n* = 4).

Root length did not differ significantly as the nutrient solution flow rate increased from 2 L/min to 6 L/min (Figure 2a) but significantly decreased at 8 L/min. Root surface area increased by 20.7% when the flow rate increased from 2 L/min to 4 L/min. Conversely, the root surface area decreased by 42.6% when the flow rate increased from 4 L/min to 6 L/min, and by 65.0% when the flow rate increased from 4 L/min to 8 L/min (Figure 2b). The root surface area was significantly larger at low flow rates (2–4 L/min) than at high flow rates (6–8 L/min). Root volume increased by 49.0% when the flow rate increased from 2 L/min to 4 L/min. Conversely, the root volume decreased by 63.0% when the flow rate increased from 4 L/min to 6 L/min, and by 74.6% when the flow rate increased from 4 L/min to 8 L/min (Figure 2c).

The root volume per unit fresh weight (VFW) increased by 18.8% when the flow rate increased from 2 L/min to 4 L/min. Conversely, the VFW decreased by 36.6% when the flow rate increased from 4 L/min to 6 L/min, and by 46.3% when the flow rate increased from 4 L/min to 8 L/min (Figure 2d). The VFW was significantly larger at low flow rates (2–4 L/min) than at high flow rates (6–8 L/min). The root surface area per unit fresh weight (SAFW) did not differ significantly as the flow rate increased from 2 L/min to 6 L/min, but significantly decreased at 8 L/min (Figure 2e). The root cellulose and hemicellulose contents per unit fresh weight (CFW; Figure 2f) and per unit volume (CV; Figure 2g) were significantly lower at low flow rates (2–4 L/min) that at high flow rates (6–8 L/min).

## 3. Discussion

The leaf is the most important organ for plants to transfer light energy to chemical energy by means of photosynthesis. Photosynthetic capacity is affected by leaf area and is closely related to plant growth [12]. Fresh and dry plant weights are considered the main indexes for measuring yield.

In this study, we found that plant growth was promoted by flow rates ranging from 2 L/min to 4 L/min, and a flow rate of 4 L/min optimized the maximum plant yield. Beyond this optimal flow rate, the plant yield decreased. We directly observed the effects of flow rate on plant growth as seen by the plant growth indexes of dry weight, fresh weight, leaf area, nutrient uptake, root length, root surface area, and root volume.

Nitrogen (N) is considered to be the most important nutrient element. N is essential in making sure plants are healthy as they develop [13]. In this study, we used N as a representative to investigate the effect of flow rate on nutrient uptake by plants. It is worth mentioning that although each plant growth index varied in its specific values, they all shared similar trends in plant growth index and N uptake with increased flow rate. Generally, these indexes increased as the flow rate increased from 2 L/min to 4 L/min, and decreased as the flow rate increased from 4 L/min to 8 L/min.

These results indicate that for a hydroponic crop, there is an optimal flow rate that will maximize root growth by increasing root length, surface area, and volume over other flow rates. Root length and root surface area are related to plants’ nutrient absorption capacity [14]. Therefore, the appropriate mechanical stimulation from the flow rate will increase root growth and facilitate higher nutrient absorption to promote overall plant growth.

We found that root VFW was significantly higher at low flow rates (2–4 L/min) than at high flow rates (6–8 L/min). Additionally, the root SAFW decreased significantly at 8 L/min compared with the other flow rates. This indicates that the roots initiated a morphological response to adapt to the flow rate environment. Decreasing VFW corresponds to increasing density and the plant becoming more compact [15]. Consequently, roots become more compact at high flow rates, which reduces the root SAFW. Decreased root surface area therefore causes the roots to uptake less N at higher flow rates.

Cellulose and hemicellulose are essential components for maintaining the root cell wall framework. Thus, the structural and compositional changes in cellulose and hemicellulose are also the main factors affecting the cell wall’s extension, elongation, and mechanical strength [16]. In this study, we found that the CFW and the CV were significantly smaller at low flow rates than at high flow rates. Moreover, the root strength was highly correlated with cellulose content [17], indicating that plants grown under high flow rates will develop stronger and more compact roots.

Plant responses are triggered following a range of biological, chemical, and physical stimuli [18,19]. The stress theory indicates a dose-response relationship between the strength and duration of the stimulus and the corresponding plant response. Moderate stimuli positively affect plant growth (eustress), whereas excessive stimuli negatively affect plant growth (distress) [20]. Several studies [21,22,23] have reported beneficial effects from applying biological, chemical, and physical eustressors in horticulture. Managed eustress dose and duration may stimulate the plant response to improve overall plant yield and quality [20]. Such managed eustress may also apply toward enhancing hydroponic production.

**Figure 3:**
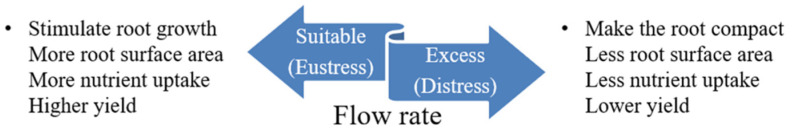
Relationship between root morphology and yield as affected by hydroponic flow rate.

In hydroponics, the flow of nutrient solution provides mechanical stimulation to the plant roots. Previous studies have shown that mechanical stimulation is related to thigmomorphogenesis [15,24,25,26,27]. In this study, low flow rates (2–4 L/min) can be regarded as eustress, and increasing the flow rate within this range can provide the roots with the appropriate mechanical stimulation for promoting root growth. Increased root growth allows the root system to absorb more nutrients, which in turn increases plant growth. Conversely, high flow rates (6–8 L/min) may be regarded as distress. To adapt to the high flow rate environment, the roots become compact, and the root surface area and overall root growth become inhibited. Root length and root surface are the key factors that determine nutrient ion absorption, and in turn, overall plant growth. Smaller root surface areas result in lower nutrient absorption, which in turn reduces overall plant growth. Our results (Figure 3) indicate that regulating the nutrient solution flow rate can regulate plant thigmomorphogenesis and nutrient absorption, and thereby increase hydroponic crop quality.

To our knowledge, there have been no previous studies on plant morphological formation as affected by nutrient solution flow rate in hydroponics. It is regrettable that we cannot compare the results of our study with others. However, reviewing previous studies on the effects of flow rate on plant growth has led us to reach similar conclusions with other researchers (Table 1). We have determined that there is an optimal flow rate for different plants to achieve the highest yield. Combined with these studies, we confirm that it is possible to regulate crop quality by regulating the flow rate’s mechanical stimulation of hydroponic crops.

In this study, according to the result of dry weight under different flow rates, the optimal flow rate is considered to be 2–6 L/min. That being said, it should be mentioned that plant growth is not only affected by the flow rate. The growth of plants is affected by a combination of the biological environment, the physical environment, and the chemical environment, which means that the optimal flow rate in this environment may not be the same in other environments (such as different nutrient solutions, light environment, cultivation container, and plant species, etc.). It is very important to find the ideal flow rate for hydroponic production because it is helpful to increase the yield [11]. At the same time, based on the above reasons, it is not easy to determine the ideal flow rate, which still needs specific analysis for different specific situations. To investigate the coupling effect of flow and other environmental factors on plants is a future topic for us. Furthermore, concerning the plant characteristics, cultivation container shape and environmental characteristics, to put forward a comprehensive index not only including flow rate to guide ideal flow regulation is also a topic for us in the future [7].

## 4. Materials and Methods

### 4.1. Cultivation and Measurement

The cultivation experiment was performed in an indoor cultivation room equipped with artificial lighting at the Faculty of Region, Tottori University, Japan. During the experiment (10 May through 31 May 2021), we used a humidity/temperature/atmospheric pressure sensor (VP-4, Decagon Devices, Pullman, WA, USA) and an ultrasonic anemometer (ATMOS22, METER Environment, Pullman, WA, USA) to record environmental data in the cultivation room (Supplementary Data Table S1).

**Figure 4:**
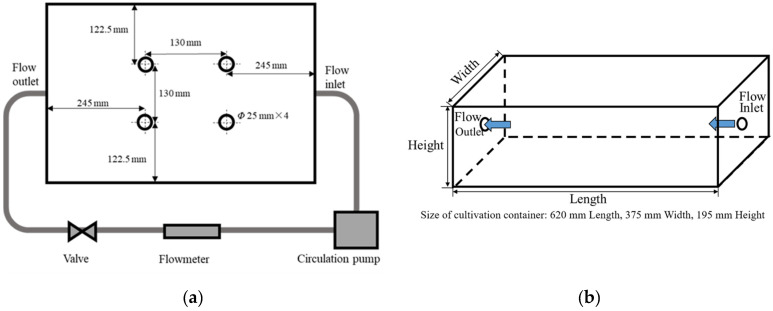
Hydroponics system used to study Swiss chard growth under variable nutrient solution flow rates. (**a**) Diagram of the setup from above. (**b**) Cultivation container orientation and dimensions.

**Figure 5:**
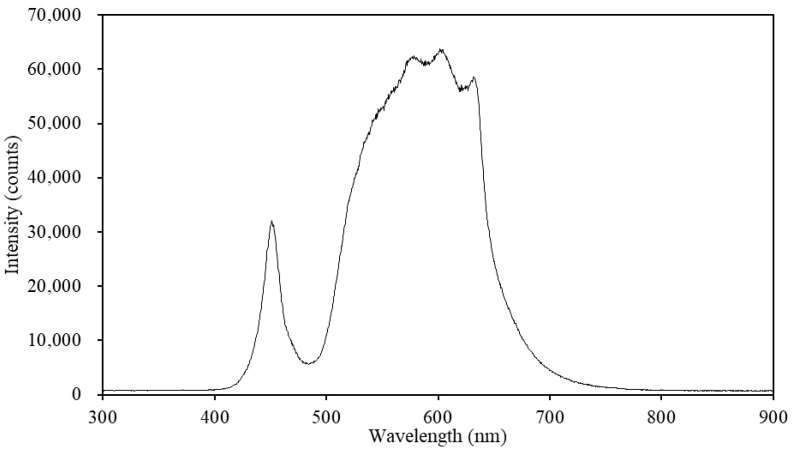
LED spectra used in this study.

We prepared a standard nutrient solution (OTA fertilizer A; Table 2) by combining OTA No. 1 fertilizer (OTA house fertilizer series, OAT Agrio, Tokyo, Japan), OTA No. 2 fertilizer (OTA house fertilizer series, OAT Agrio, Tokyo, Japan), and tap water (pH 7.0, EC 0.07 mS/cm) [32]. Swiss chard (*Beta vulgaris* L. spp. *cicla* cv. Seiyou Shirokuki), a halophyte plant that plays a considerable role in arid and semi-arid land diets, was sown in vermiculite. When the first true leaves appeared, the seedlings were transplanted into plastic containers (620 mm Length, 375 mm Width, 195 mm Height) filled with 40 L of non-flowing diluted nutrient solution (OTA fertilizer A solution at 0.25 concentration with pH 6.5 and EC 0.68 mS/cm). We illuminated the seedlings using LEDs (Derlights B07YV633CT ShenZhen Lighting Inc., Shenzhen, China) on a 12:12 light:dark schedule. We set the photosynthetic photon flux density (PPFD) at the surface of the cultivation plate to 900 μmol/m^2^·s. After ten days, we transplanted the seedlings into four-hole floating panels and installed them into sixteen 40 L hydroponic cultivation systems containing nutrient solution (OTA fertilizer A at 0.5 concentration with pH 6.5 and EC of 1.26 mS/cm). The hydroponic cultivation system (Figure 4) consisted of a 620 mm × 375 mm × 195 mm cultivation container, a pump (DC40A, ZKSJ, Shenzhen Century Zhongke Technology Co., Ltd., Shenzhen, Guangdong, China), a valve, and a digital flow sensor (Sea Zhongjiang, Jinan, China). Each cultivation container’s inlet and outlet flows had a diameter of 18 mm and were positioned at the center of container’s sidewall. The nutrient solution was circulated by a pump continuously. Each cultivation container received a different flow rate. The experimental flow rates were 2 L/min, 4 L/min, 6 L/min, and 8 L/min, and were established using the valve. LEDs were mounted above each cultivation container, and the PPFD at the surface of each cultivation plate was set at 900 μmol/m^2^·s. The LED spectrum was measured by a fiber-optic spectrometer (USB2000+, Ocean Optics Inc., Ostfildern, Germany) 1 m below the LEDs (Figure 5). Each of the four flow rate treatments consisted of four replicates (i.e., hydroponic cultivation systems), and four plants were planted in each cultivation container. The nutrient solution was replaced every 14 days.

The plants in each cultivation container were harvested after 21 days and separated into shoots and roots. Each plant’s fresh weight and leaf area were measured using a precision balance (UP623Y, Shimadzu, Kyoto, Japan) and leaf area meter (LI-3100 AREA METER, LI-COR Biosciences, Lincoln, NE, USA). Root length, root surface area, and root volume were measured by a root scanner and processed by software (WinRhizo 2008a, REGENT INS, Quebec, QC, Canada). The plants were then placed into a convection oven (DKM600, Yamato Scientific, Tokyo, Japan) at 75 °C and dried for 72 h before measuring dry weight. Next, the dried plants were crushed to measure their nutrient content. The total nitrogen content in each shoot and root sample was measured using an organic elemental analyzer (CN corder JM1000CN, J-Science Group, Tokyo, Japan). The whole-plant N uptake was then calculated according to the dry weight (Table S2) and N contents (Table S3).

The root cell wall hemicellulose and cellulose contents were analyzed according to the modified procedure of Ping An et al. [33]. First, the dried root was crushed into powder by machine, and about 20 mg root powder was added into a test tube. Then, the pellet-containing cell walls were further purified by sequential incubation and centrifugation in ethanol, acetone, and a methanol:chloroform mixture (1:1 *v*/*v*). Hemicelluloses I and II were sequentially extracted with 1 M and 4 M KOH. The extractions were repeated three times at intervals of 8, 16, and 8 h. The residual insoluble sediments were then successively washed with water and ethanol and dried at 50 °C. The residues were designated as cellulose fractions after dissolving in 2 mL of 72% (*v*/*v*) sulfuric acid and diluting with 8 mL water. The total sugar in each fraction was measured using the phenol–sulfuric acid method [34].

The dry weight hemicellulose I and II contents (Table S4) were added together to calculate the total dry weight hemicellulose content of the root cell wall. The root hemicellulose CFW was then calculated by multiplying the root dry weight (Table S2) by the dry weight hemicellulose content and dividing by the root fresh weight (Table S2). Similarly, the root hemicellulose CV was obtained by multiplying the root dry weight by the dry weight hemicellulose content and then dividing by the root volume (Table S3).

We calculated the root cellulose CFW by multiplying the root dry weight by the dry weight cellulose content (Table S4) and dividing by the root fresh weight. Similarly, the root cellulose CV was obtained by multiplying the root dry weight by the dry weight cellulose content and then dividing by the root volume.

### 4.2. Data Analysis

We used SPSS (version 25, IBM, Chicago, IL, USA) for data analysis. We used a one-way analysis of variance followed by Duncan’s multiple range test with results considered statistically significant at *p* < 0.05. The statistical results were expressed as means ± standard error (*n* = 4).

## 5. Conclusions

In drylands, plants are exposed to different biotic and abiotic stress factors such as drought, salt stress, and water shortage. These stress factors limit plant yield, growth, quality, and appearance. Agricultural and horticultural practices for food production in dryland aim to minimize stress by using suitable production systems, either under controllable or open-field environments. Hydroponic cultivation is widely used in drylands as a form of controlled environmental agriculture to alleviate stresses during crop production. The flow of nutrient solution in the hydroponic substrate mechanically stimulates plants and affects plant thigmomorphogenesis.

A suitable flow rate can be regarded as a eustress. In the range of suitable flow rates, increased flow provides plant roots with an appropriate level of mechanical stimulation to promote root growth. Increased root growth allows the root system to absorb more nutrients, which makes the plant grow better. Conversely, excess flow rates may be regarded as stress. To adapt to the high flow environment, plant roots become compact, which inhibits the root surface area and root growth. Root length and surface area are key factors that determine a plant’s capability for nutrient ion absorption, which in turn affects the overall plant growth compared with optimal flow rate conditions. Our results indicate that regulating flow rate can regulate plant thigmomorphogenesis and nutrient absorption, and therefore affect crop quality in hydroponics.

Determining the ideal flow rate for hydroponic production may help increase yield. However, such a determination requires specific analysis of each crop and growing environment. We intend to investigate the coupling effects of flow rate and other environmental factors on plants in future studies, and to develop a comprehensive index of other plant characteristics, including cultivation container shape and environmental characteristics, to guide the ideal flow regulation under different growth scenarios.

## Figures and Tables

**Table 1:** Summary of previous studies on the effect of flow rate on hydroponic plant growth.

Study	Flow Rate (L/min) (Optimal Value for Best Yield)	Plant Species
Genuncio et al. (2000) [10]	0.75, 1, 1.5 (1.5)	Lettuce
AI-Tawaha et al. (2018) [11]	10, 20, 30 (20)	
Dalastra et al. (2020) [8]	0.5, 1.0, 2.0, 4.0 (1.0)	
Khater et al. (2015) [28]	1.0, 1.5, 2.0 (1.0)	
Nuwansi et al. (2016) [29]	0.8, 2.4, 4.0 (0.8)	Spinach
Endut et al. (2009) [30]	0.8, 1.6, 2.4, 3.2, 4.0 (1.6)	
Hussain et al. (2015) [31]	1.0, 1.5, 3.2 (1.5)	
Bateer et al. (2021) [7]	0, 2, 4, 6, 8 (6)	Swiss chard
Hammady et al. (2020) [9]	1.5, 2.5 (1.5)	Cauliflower

**Table 2:** Composition of the OTA fertilizer A standard nutrient solution.

Composition	T-N	P_2_O_5_	K_2_O	CaO	MgO	MnO	B_2_O_3_	Fe	Cu	Zn	Mo
**Concentration (ppm)**	260	120	405	230	60	1.5	1.5	2.7	0.03	0.09	0.03

## Data Availability

All data generated or analyzed during this study are included in this published article.

## Supplementary Materials

The following are available online at https://www.mdpi.com/article/10.3390/plants10091840/s1, Table S1: The environmental data in the cultivation room, Table S2: Detail data of leaf area, fresh weight, and dry weight under different flow rates in this study, Table S3: Detail data of root morphology and N contents of plants under different flow rates in this study, and Table S4: Detail data of hemicellulose I, II, and cellulose contents of roots under different flow rates in this study.
