# Supplementary material for: Effect of Nutrient Solution Flow Rate on Hydroponic Plant Growth and Root Morphology
Source: Plants (Basel). 2021 Sep 5;10(9):1840. doi: 10.3390/plants10091840 (PMC8465728; doi:10.3390/plants10091840)
Supplement: Supplementary file 1 [file plants-10-01840-s001.zip › plants-1359110-supplementary/Table S1. The environmental data in the cultivation room..pdf]

Table S1. The environmental data in the cultivation room.

	Humidity	Tempurature	Barometer	Ultrasonic Anemometer
Measurement Time	RH	℃	kPa Pressure	m/s
2021/5/10 0:00	0.666	24.6	100.88	0.06
2021/5/10 0:10	0.666	24.6	100.88	0.06
2021/5/10 0:20	0.663	24.6	100.87	0.05
2021/5/10 0:30	0.666	24.6	100.87	0.05
2021/5/10 0:40	0.663	24.6	100.87	0.05
2021/5/10 0:50	0.663	24.6	100.87	0.06
2021/5/10 1:00	0.667	24.5	100.87	0.06
2021/5/10 1:10	0.667	24.5	100.87	0.06
2021/5/10 1:20	0.667	24.5	100.87	0.06
2021/5/10 1:30	0.670	24.5	100.87	0.06
2021/5/10 1:40	0.667	24.5	100.85	0.06
2021/5/10 1:50	0.667	24.5	100.85	0.06
2021/5/10 2:00	0.667	24.5	100.84	0.05
2021/5/10 2:10	0.667	24.5	100.84	0.06
2021/5/10 2:20	0.667	24.5	100.85	0.06
2021/5/10 2:30	0.671	24.4	100.85	0.06
2021/5/10 2:40	0.671	24.4	100.85	0.06
2021/5/10 2:50	0.671	24.4	100.84	0.05
2021/5/10 3:00	0.671	24.4	100.84	0.06
2021/5/10 3:10	0.671	24.4	100.84	0.05
2021/5/10 3:20	0.671	24.4	100.85	0.05
2021/5/10 3:30	0.671	24.4	100.87	0.06
2021/5/10 3:40	0.671	24.4	100.85	0.06
2021/5/10 3:50	0.671	24.4	100.84	0.05
2021/5/10 4:00	0.675	24.3	100.85	0.06
2021/5/10 4:10	0.675	24.3	100.85	0.06
2021/5/10 4:20	0.675	24.3	100.85	0.06
2021/5/10 4:30	0.675	24.3	100.85	0.07
2021/5/10 4:40	0.675	24.3	100.87	0.06
2021/5/10 4:50	0.678	24.3	100.88	0.06
2021/5/10 5:00	0.678	24.3	100.87	0.05
2021/5/10 5:10	0.678	24.3	100.88	0.06
2021/5/10 5:20	0.683	24.2	100.90	0.06
2021/5/10 5:30	0.683	24.2	100.90	0.06
2021/5/10 5:40	0.683	24.2	100.90	0.05
2021/5/10 5:50	0.683	24.2	100.91	0.05
2021/5/10 6:00	0.683	24.2	100.92	0.06
2021/5/10 6:10	0.683	24.2	100.94	0.06
2021/5/10 6:20	0.683	24.2	100.95	0.06
2021/5/10 6:30	0.678	24.4	100.95	0.11
2021/5/10 6:40	0.673	24.6	100.97	0.14
2021/5/10 6:50	0.668	24.8	100.97	0.14
2021/5/10 7:00	0.667	24.9	100.94	0.13
2021/5/10 7:10	0.667	24.9	100.94	0.14
2021/5/10 7:20	0.663	25.0	100.95	0.13
2021/5/10 7:30	0.663	25.1	100.95	0.14
2021/5/10 7:40	0.663	25.1	100.97	0.14
2021/5/10 7:50	0.663	25.1	100.95	0.13
2021/5/10 8:00	0.663	25.1	100.98	0.14
2021/5/10 8:10	0.659	25.2	101.00	0.14
2021/5/10 8:20	0.659	25.2	101.01	0.14
2021/5/10 8:30	0.659	25.2	101.01	0.13
2021/5/10 8:40	0.657	25.4	101.00	0.14

2021/5/10 8:50	0.648	25.7	100.98	0.13
2021/5/10 9:00	0.637	26.0	100.95	0.13
2021/5/10 9:10	0.636	26.1	100.98	0.13
2021/5/10 9:20	0.632	26.2	100.98	0.13
2021/5/10 9:30	0.632	26.2	101.00	0.13
2021/5/10 9:40	0.632	26.3	101.03	0.11
2021/5/10 9:50	0.632	26.3	101.01	0.13
2021/5/10 10:00	0.628	26.4	101.00	0.12
2021/5/10 10:10	0.628	26.4	101.00	0.12
2021/5/10 10:20	0.624	26.5	100.98	0.11
2021/5/10 10:30	0.624	26.5	101.00	0.12
2021/5/10 10:40	0.627	26.5	101.00	0.11
2021/5/10 10:50	0.623	26.6	101.01	0.10
2021/5/10 11:00	0.626	26.6	101.01	0.09
2021/5/10 11:10	0.620	26.7	101.01	0.08
2021/5/10 11:20	0.623	26.7	101.01	0.06
2021/5/10 11:30	0.616	26.8	101.04	0.07
2021/5/10 11:40	0.622	26.8	101.01	0.08
2021/5/10 11:50	0.619	26.8	101.01	0.09
2021/5/10 12:00	0.622	26.8	101.01	0.06
2021/5/10 12:10	0.616	26.8	101.00	0.06
2021/5/10 12:20	0.622	26.8	101.00	0.06
2021/5/10 12:30	0.615	26.9	101.01	0.06
2021/5/10 12:40	0.615	26.9	101.01	0.05
2021/5/10 12:50	0.615	26.9	101.01	0.05
2021/5/10 13:00	0.615	26.9	101.03	0.05
2021/5/10 13:10	0.613	26.9	101.01	0.05
2021/5/10 13:20	0.613	26.9	101.01	0.05
2021/5/10 13:30	0.613	26.9	101.01	0.04
2021/5/10 13:40	0.615	26.9	101.00	0.05
2021/5/10 13:50	0.613	26.9	101.00	0.05
2021/5/10 14:00	0.613	26.9	101.00	0.06
2021/5/10 14:10	0.613	26.9	100.98	0.06
2021/5/10 14:20	0.609	27.0	100.97	0.06
2021/5/10 14:30	0.612	27.0	100.98	0.05
2021/5/10 14:40	0.609	27.0	100.98	0.06
2021/5/10 14:50	0.603	27.0	100.98	0.06
2021/5/10 15:00	0.603	27.0	100.97	0.06
2021/5/10 15:10	0.595	27.0	100.97	0.09
2021/5/10 15:20	0.607	26.5	100.97	0.14
2021/5/10 15:30	0.618	26.1	100.95	0.13
2021/5/10 15:40	0.626	25.9	100.97	0.14
2021/5/10 15:50	0.629	25.8	100.95	0.13
2021/5/10 16:00	0.624	26.2	100.97	0.11
2021/5/10 16:10	0.616	26.5	100.98	0.12
2021/5/10 16:20	0.608	26.7	100.98	0.12
2021/5/10 16:30	0.608	26.8	101.00	0.12
2021/5/10 16:40	0.608	26.8	101.00	0.12
2021/5/10 16:50	0.604	26.9	101.01	0.11
2021/5/10 17:00	0.610	26.8	101.01	0.12
2021/5/10 17:10	0.608	26.8	101.01	0.11
2021/5/10 17:20	0.610	26.8	101.01	0.12
2021/5/10 17:30	0.610	26.8	101.03	0.11
2021/5/10 17:40	0.610	26.8	101.03	0.12
2021/5/10 17:50	0.610	26.8	101.03	0.12
2021/5/10 18:00	0.610	26.8	101.03	0.12

2021/5/10 18:10	0.614	26.7	101.04	0.12
2021/5/10 18:20	0.615	26.6	101.04	0.11
2021/5/10 18:30	0.620	26.3	101.06	0.10
2021/5/10 18:40	0.636	25.7	101.07	0.10
2021/5/10 18:50	0.642	25.4	101.07	0.09
2021/5/10 19:00	0.649	25.2	101.06	0.08
2021/5/10 19:10	0.654	25.0	101.07	0.06
2021/5/10 19:20	0.655	24.9	101.07	0.06
2021/5/10 19:30	0.658	24.8	101.07	0.06
2021/5/10 19:40	0.658	24.8	101.09	0.05
2021/5/10 19:50	0.662	24.7	101.10	0.05
2021/5/10 20:00	0.659	24.7	101.12	0.06
2021/5/10 20:10	0.663	24.6	101.10	0.05
2021/5/10 20:20	0.663	24.6	101.10	0.05
2021/5/10 20:30	0.663	24.6	101.12	0.05
2021/5/10 20:40	0.664	24.5	101.12	0.05
2021/5/10 20:50	0.664	24.5	101.13	0.04
2021/5/10 21:00	0.664	24.5	101.16	0.04
2021/5/10 21:10	0.664	24.5	101.17	0.05
2021/5/10 21:20	0.668	24.4	101.19	0.04
2021/5/10 21:30	0.665	24.4	101.19	0.05
2021/5/10 21:40	0.665	24.4	101.20	0.05
2021/5/10 21:50	0.669	24.3	101.20	0.05
2021/5/10 22:00	0.669	24.3	101.19	0.05
2021/5/10 22:10	0.665	24.3	101.19	0.05
2021/5/10 22:20	0.665	24.3	101.19	0.05
2021/5/10 22:30	0.669	24.2	101.19	0.06
2021/5/10 22:40	0.666	24.2	101.22	0.06
2021/5/10 22:50	0.666	24.2	101.20	0.06
2021/5/10 23:00	0.666	24.2	101.19	0.05
2021/5/10 23:10	0.663	24.2	101.20	0.05
2021/5/10 23:20	0.667	24.1	101.23	0.06
2021/5/10 23:30	0.667	24.1	101.25	0.06
2021/5/10 23:40	0.667	24.1	101.25	0.06
2021/5/10 23:50	0.663	24.1	101.23	0.05
2021/5/11 0:00	0.663	24.1	101.20	0.06
2021/5/11 0:10	0.663	24.1	101.19	0.06
2021/5/11 0:20	0.664	24.0	101.17	0.05
2021/5/11 0:30	0.664	24.0	101.14	0.05
2021/5/11 0:40	0.664	24.0	101.16	0.06
2021/5/11 0:50	0.661	24.0	101.14	0.05
2021/5/11 1:00	0.665	23.9	101.16	0.05
2021/5/11 1:10	0.665	23.9	101.14	0.05
2021/5/11 1:20	0.665	23.9	101.13	0.05
2021/5/11 1:30	0.661	23.9	101.12	0.06
2021/5/11 1:40	0.661	23.9	101.09	0.05
2021/5/11 1:50	0.661	23.9	101.12	0.06
2021/5/11 2:00	0.662	23.8	101.13	0.07
2021/5/11 2:10	0.662	23.8	101.13	0.05
2021/5/11 2:20	0.662	23.8	101.12	0.06
2021/5/11 2:30	0.658	23.8	101.09	0.06
2021/5/11 2:40	0.658	23.8	101.07	0.05
2021/5/11 2:50	0.658	23.8	101.09	0.05
2021/5/11 3:00	0.659	23.7	101.12	0.04
2021/5/11 3:10	0.659	23.7	101.12	0.05
2021/5/11 3:20	0.659	23.7	101.12	0.06

2021/5/11 3:30	0.656	23.7	101.12	0.05
2021/5/11 3:40	0.660	23.6	101.12	0.05
2021/5/11 3:50	0.660	23.6	101.13	0.06
2021/5/11 4:00	0.660	23.6	101.13	0.06
2021/5/11 4:10	0.660	23.6	101.13	0.05
2021/5/11 4:20	0.660	23.6	101.16	0.06
2021/5/11 4:30	0.656	23.6	101.14	0.05
2021/5/11 4:40	0.656	23.6	101.13	0.05
2021/5/11 4:50	0.660	23.5	101.12	0.04
2021/5/11 5:00	0.660	23.5	101.10	0.05
2021/5/11 5:10	0.657	23.5	101.10	0.05
2021/5/11 5:20	0.657	23.5	101.09	0.05
2021/5/11 5:30	0.657	23.5	101.09	0.05
2021/5/11 5:40	0.657	23.5	101.10	0.04
2021/5/11 5:50	0.661	23.4	101.10	0.05
2021/5/11 6:00	0.657	23.4	101.06	0.05
2021/5/11 6:10	0.657	23.4	101.06	0.05
2021/5/11 6:20	0.657	23.5	101.07	0.06
2021/5/11 6:30	0.652	23.7	101.09	0.07
2021/5/11 6:40	0.643	24.2	101.07	0.11
2021/5/11 6:50	0.631	24.6	101.07	0.12
2021/5/11 7:00	0.627	24.8	101.07	0.13
2021/5/11 7:10	0.619	25.1	101.07	0.14
2021/5/11 7:20	0.618	25.2	101.06	0.14
2021/5/11 7:30	0.614	25.3	101.04	0.15
2021/5/11 7:40	0.614	25.3	101.04	0.14
2021/5/11 7:50	0.611	25.4	101.04	0.14
2021/5/11 8:00	0.614	25.4	101.03	0.14
2021/5/11 8:10	0.610	25.5	101.03	0.15
2021/5/11 8:20	0.610	25.5	101.01	0.15
2021/5/11 8:30	0.607	25.6	101.00	0.16
2021/5/11 8:40	0.606	25.7	100.98	0.15
2021/5/11 8:50	0.606	25.7	100.98	0.15
2021/5/11 9:00	0.606	25.7	100.97	0.15
2021/5/11 9:10	0.609	25.7	100.97	0.15
2021/5/11 9:20	0.605	25.8	100.95	0.15
2021/5/11 9:30	0.605	25.8	100.92	0.15
2021/5/11 9:40	0.605	25.8	100.91	0.15
2021/5/11 9:50	0.605	25.9	100.88	0.16
2021/5/11 10:00	0.605	25.9	100.87	0.16
2021/5/11 10:10	0.601	26.0	100.84	0.15
2021/5/11 10:20	0.601	26.1	100.81	0.14
2021/5/11 10:30	0.600	26.2	100.79	0.14
2021/5/11 10:40	0.597	26.3	100.78	0.13
2021/5/11 10:50	0.597	26.3	100.78	0.13
2021/5/11 11:00	0.597	26.3	100.78	0.15
2021/5/11 11:10	0.597	26.3	100.73	0.15
2021/5/11 11:20	0.591	26.3	100.72	0.15
2021/5/11 11:30	0.588	26.3	100.70	0.15
2021/5/11 11:40	0.585	26.3	100.69	0.15
2021/5/11 11:50	0.582	26.3	100.69	0.16
2021/5/11 12:00	0.579	26.3	100.69	0.15
2021/5/11 12:10	0.576	26.3	100.70	0.15
2021/5/11 12:20	0.573	26.3	100.70	0.14
2021/5/11 12:30	0.570	26.3	100.69	0.15
2021/5/11 12:40	0.570	26.3	100.69	0.13

2021/5/11 12:50	0.567	26.3	100.69	0.15
2021/5/11 13:00	0.571	26.2	100.69	0.16
2021/5/11 13:10	0.571	26.2	100.69	0.16
2021/5/11 13:20	0.571	26.2	100.66	0.14
2021/5/11 13:30	0.577	26.2	100.63	0.15
2021/5/11 13:40	0.583	26.1	100.60	0.16
2021/5/11 13:50	0.582	26.2	100.60	0.16
2021/5/11 14:00	0.585	26.2	100.60	0.16
2021/5/11 14:10	0.588	26.2	100.59	0.16
2021/5/11 14:20	0.592	26.1	100.59	0.15
2021/5/11 14:30	0.592	26.1	100.57	0.15
2021/5/11 14:40	0.592	26.1	100.54	0.14
2021/5/11 14:50	0.592	26.1	100.50	0.15
2021/5/11 15:00	0.592	26.1	100.51	0.16
2021/5/11 15:10	0.592	26.1	100.49	0.14
2021/5/11 15:20	0.592	26.1	100.46	0.15
2021/5/11 15:30	0.595	26.1	100.44	0.15
2021/5/11 15:40	0.592	26.1	100.41	0.14
2021/5/11 15:50	0.598	26.0	100.38	0.12
2021/5/11 16:00	0.609	25.7	100.37	0.14
2021/5/11 16:10	0.613	25.5	100.38	0.14
2021/5/11 16:20	0.617	25.4	100.34	0.15
2021/5/11 16:30	0.621	25.3	100.32	0.14
2021/5/11 16:40	0.624	25.3	100.34	0.15
2021/5/11 16:50	0.624	25.3	100.29	0.14
2021/5/11 17:00	0.624	25.3	100.28	0.14
2021/5/11 17:10	0.627	25.3	100.28	0.15
2021/5/11 17:20	0.627	25.3	100.27	0.14
2021/5/11 17:30	0.630	25.3	100.27	0.15
2021/5/11 17:40	0.630	25.3	100.25	0.14
2021/5/11 17:50	0.630	25.3	100.24	0.14
2021/5/11 18:00	0.633	25.3	100.24	0.14
2021/5/11 18:10	0.633	25.3	100.22	0.15
2021/5/11 18:20	0.633	25.3	100.22	0.12
2021/5/11 18:30	0.634	25.2	100.22	0.07
2021/5/11 18:40	0.634	25.1	100.21	0.07
2021/5/11 18:50	0.635	25.0	100.18	0.06
2021/5/11 19:00	0.643	24.8	100.16	0.06
2021/5/11 19:10	0.643	24.7	100.15	0.06
2021/5/11 19:20	0.643	24.7	100.16	0.06
2021/5/11 19:30	0.647	24.6	100.18	0.05
2021/5/11 19:40	0.651	24.5	100.18	0.06
2021/5/11 19:50	0.651	24.5	100.19	0.05
2021/5/11 20:00	0.658	24.4	100.22	0.06
2021/5/11 20:10	0.658	24.4	100.21	0.07
2021/5/11 20:20	0.658	24.4	100.21	0.06
2021/5/11 20:30	0.658	24.4	100.19	0.06
2021/5/11 20:40	0.662	24.3	100.21	0.06
2021/5/11 20:50	0.662	24.3	100.25	0.06
2021/5/11 21:00	0.662	24.3	100.25	0.07
2021/5/11 21:10	0.662	24.3	100.25	0.07
2021/5/11 21:20	0.665	24.3	100.25	0.07
2021/5/11 21:30	0.665	24.3	100.27	0.06
2021/5/11 21:40	0.665	24.3	100.28	0.06
2021/5/11 21:50	0.669	24.3	100.28	0.06
2021/5/11 22:00	0.673	24.2	100.28	0.07

2021/5/11 22:10	0.673	24.2	100.27	0.07
2021/5/11 22:20	0.673	24.2	100.27	0.06
2021/5/11 22:30	0.673	24.2	100.25	0.06
2021/5/11 22:40	0.676	24.2	100.25	0.06
2021/5/11 22:50	0.676	24.2	100.25	0.07
2021/5/11 23:00	0.676	24.2	100.24	0.07
2021/5/11 23:10	0.676	24.2	100.22	0.07
2021/5/11 23:20	0.679	24.2	100.22	0.08
2021/5/11 23:30	0.679	24.2	100.21	0.07
2021/5/11 23:40	0.679	24.2	100.21	0.07
2021/5/11 23:50	0.679	24.2	100.19	0.06
2021/5/12 0:00	0.679	24.2	100.16	0.06
2021/5/12 0:10	0.687	24.1	100.15	0.06
2021/5/12 0:20	0.687	24.1	100.12	0.06
2021/5/12 0:30	0.687	24.1	100.10	0.07
2021/5/12 0:40	0.687	24.1	100.05	0.07
2021/5/12 0:50	0.690	24.1	100.02	0.06
2021/5/12 1:00	0.690	24.1	100.00	0.07
2021/5/12 1:10	0.690	24.1	100.00	0.07
2021/5/12 1:20	0.690	24.1	99.97	0.06
2021/5/12 1:30	0.690	24.1	99.97	0.05
2021/5/12 1:40	0.690	24.1	99.97	0.07
2021/5/12 1:50	0.693	24.1	99.97	0.07
2021/5/12 2:00	0.693	24.1	99.99	0.06
2021/5/12 2:10	0.693	24.1	100.02	0.07
2021/5/12 2:20	0.698	24.0	100.05	0.06
2021/5/12 2:30	0.698	24.0	100.05	0.05
2021/5/12 2:40	0.701	24.0	100.07	0.06
2021/5/12 2:50	0.701	24.0	100.06	0.07
2021/5/12 3:00	0.701	24.0	100.03	0.07
2021/5/12 3:10	0.701	24.0	100.00	0.06
2021/5/12 3:20	0.701	24.0	99.97	0.06
2021/5/12 3:30	0.704	24.0	99.97	0.07
2021/5/12 3:40	0.704	24.0	99.93	0.07
2021/5/12 3:50	0.704	24.0	99.87	0.07
2021/5/12 4:00	0.704	24.0	99.84	0.07
2021/5/12 4:10	0.704	24.0	99.84	0.06
2021/5/12 4:20	0.704	24.0	99.84	0.07
2021/5/12 4:30	0.708	23.9	99.85	0.07
2021/5/12 4:40	0.708	23.9	99.87	0.07
2021/5/12 4:50	0.708	23.9	99.93	0.06
2021/5/12 5:00	0.712	23.9	99.96	0.07
2021/5/12 5:10	0.708	23.9	99.94	0.07
2021/5/12 5:20	0.712	23.9	99.96	0.06
2021/5/12 5:30	0.712	23.9	99.99	0.07
2021/5/12 5:40	0.712	23.9	100.00	0.07
2021/5/12 5:50	0.712	23.9	100.00	0.06
2021/5/12 6:00	0.712	23.9	99.97	0.07
2021/5/12 6:10	0.712	23.9	100.00	0.07
2021/5/12 6:20	0.715	23.9	100.03	0.08
2021/5/12 6:30	0.710	24.1	100.05	0.17
2021/5/12 6:40	0.705	24.3	100.00	0.18
2021/5/12 6:50	0.703	24.5	100.06	0.17
2021/5/12 7:00	0.702	24.6	100.07	0.18
2021/5/12 7:10	0.698	24.7	100.07	0.16
2021/5/12 7:20	0.701	24.7	100.09	0.17

2021/5/12 7:30	0.697	24.8	100.10	0.16
2021/5/12 7:40	0.700	24.8	100.09	0.15
2021/5/12 7:50	0.700	24.8	100.09	0.15
2021/5/12 8:00	0.700	24.8	100.10	0.16
2021/5/12 8:10	0.696	24.9	100.13	0.16
2021/5/12 8:20	0.699	24.9	100.13	0.17
2021/5/12 8:30	0.699	24.9	100.13	0.16
2021/5/12 8:40	0.699	24.9	100.10	0.17
2021/5/12 8:50	0.699	24.9	100.12	0.17
2021/5/12 9:00	0.695	25.0	100.15	0.17
2021/5/12 9:10	0.695	25.0	100.15	0.16
2021/5/12 9:20	0.695	25.0	100.15	0.16
2021/5/12 9:30	0.695	25.0	100.15	0.16
2021/5/12 9:40	0.695	25.0	100.16	0.15
2021/5/12 9:50	0.694	25.1	100.16	0.16
2021/5/12 10:00	0.694	25.1	100.16	0.16
2021/5/12 10:10	0.694	25.1	100.15	0.16
2021/5/12 10:20	0.693	25.2	100.15	0.16
2021/5/12 10:30	0.693	25.2	100.16	0.17
2021/5/12 10:40	0.690	25.2	100.16	0.15
2021/5/12 10:50	0.693	25.2	100.16	0.15
2021/5/12 11:00	0.692	25.3	100.15	0.16
2021/5/12 11:10	0.704	25.3	100.15	0.15
2021/5/12 11:20	0.706	25.4	100.12	0.15
2021/5/12 11:30	0.708	25.5	100.12	0.16
2021/5/12 11:40	0.708	25.5	100.12	0.17
2021/5/12 11:50	0.707	25.6	100.12	0.16
2021/5/12 12:00	0.710	25.6	100.12	0.17
2021/5/12 12:10	0.706	25.7	100.09	0.16
2021/5/12 12:20	0.706	25.7	100.10	0.17
2021/5/12 12:30	0.702	25.8	100.09	0.17
2021/5/12 12:40	0.702	25.8	100.12	0.17
2021/5/12 12:50	0.703	25.7	100.13	0.16
2021/5/12 13:00	0.703	25.7	100.12	0.16
2021/5/12 13:10	0.689	25.6	100.10	0.18
2021/5/12 13:20	0.712	25.2	100.09	0.15
2021/5/12 13:30	0.704	25.3	100.09	0.15
2021/5/12 13:40	0.700	25.4	100.09	0.15
2021/5/12 13:50	0.696	25.5	100.10	0.16
2021/5/12 14:00	0.692	25.6	100.12	0.15
2021/5/12 14:10	0.689	25.6	100.13	0.16
2021/5/12 14:20	0.689	25.6	100.15	0.16
2021/5/12 14:30	0.689	25.6	100.16	0.15
2021/5/12 14:40	0.683	25.6	100.16	0.16
2021/5/12 14:50	0.683	25.6	100.15	0.14
2021/5/12 15:00	0.680	25.6	100.15	0.15
2021/5/12 15:10	0.680	25.6	100.16	0.16
2021/5/12 15:20	0.677	25.6	100.16	0.15
2021/5/12 15:30	0.674	25.6	100.18	0.14
2021/5/12 15:40	0.671	25.6	100.19	0.15
2021/5/12 15:50	0.675	25.5	100.19	0.15
2021/5/12 16:00	0.671	25.5	100.21	0.14
2021/5/12 16:10	0.664	25.6	100.22	0.15
2021/5/12 16:20	0.668	25.5	100.24	0.15
2021/5/12 16:30	0.661	25.6	100.22	0.17
2021/5/12 16:40	0.661	25.6	100.24	0.15

2021/5/12 16:50	0.661	25.6	100.24	0.15
2021/5/12 17:00	0.658	25.6	100.25	0.15
2021/5/12 17:10	0.662	25.5	100.25	0.15
2021/5/12 17:20	0.655	25.6	100.25	0.15
2021/5/12 17:30	0.659	25.5	100.25	0.14
2021/5/12 17:40	0.659	25.5	100.27	0.15
2021/5/12 17:50	0.656	25.5	100.25	0.15
2021/5/12 18:00	0.656	25.5	100.25	0.15
2021/5/12 18:10	0.653	25.5	100.28	0.14
2021/5/12 18:20	0.653	25.5	100.28	0.13
2021/5/12 18:30	0.650	25.5	100.29	0.07
2021/5/12 18:40	0.652	25.3	100.29	0.06
2021/5/12 18:50	0.652	25.2	100.29	0.06
2021/5/12 19:00	0.653	25.1	100.29	0.07
2021/5/12 19:10	0.654	25.0	100.32	0.06
2021/5/12 19:20	0.655	24.9	100.34	0.06
2021/5/12 19:30	0.655	24.8	100.35	0.08
2021/5/12 19:40	0.659	24.7	100.37	0.07
2021/5/12 19:50	0.656	24.7	100.37	0.07
2021/5/12 20:00	0.656	24.7	100.37	0.06
2021/5/12 20:10	0.657	24.6	100.38	0.06
2021/5/12 20:20	0.657	24.6	100.40	0.06
2021/5/12 20:30	0.653	24.6	100.40	0.07
2021/5/12 20:40	0.657	24.5	100.40	0.05
2021/5/12 20:50	0.657	24.5	100.41	0.06
2021/5/12 21:00	0.654	24.5	100.43	0.06
2021/5/12 21:10	0.654	24.5	100.44	0.05
2021/5/12 21:20	0.654	24.5	100.46	0.06
2021/5/12 21:30	0.655	24.4	100.47	0.05
2021/5/12 21:40	0.655	24.4	100.47	0.06
2021/5/12 21:50	0.652	24.4	100.46	0.05
2021/5/12 22:00	0.652	24.4	100.46	0.05
2021/5/12 22:10	0.648	24.4	100.47	0.05
2021/5/12 22:20	0.652	24.3	100.49	0.05
2021/5/12 22:30	0.649	24.3	100.47	0.06
2021/5/12 22:40	0.649	24.3	100.47	0.06
2021/5/12 22:50	0.649	24.3	100.49	0.05
2021/5/12 23:00	0.646	24.3	100.47	0.06
2021/5/12 23:10	0.646	24.3	100.49	0.06
2021/5/12 23:20	0.646	24.2	100.49	0.05
2021/5/12 23:30	0.646	24.2	100.49	0.05
2021/5/12 23:40	0.646	24.2	100.47	0.06
2021/5/12 23:50	0.643	24.2	100.47	0.05
2021/5/13 0:00	0.643	24.2	100.47	0.06
2021/5/13 0:10	0.643	24.2	100.47	0.06
2021/5/13 0:20	0.639	24.2	100.47	0.05
2021/5/13 0:30	0.643	24.1	100.47	0.06
2021/5/13 0:40	0.643	24.1	100.49	0.06
2021/5/13 0:50	0.643	24.1	100.47	0.05
2021/5/13 1:00	0.643	24.1	100.47	0.06
2021/5/13 1:10	0.640	24.1	100.46	0.06
2021/5/13 1:20	0.640	24.1	100.46	0.06
2021/5/13 1:30	0.640	24.1	100.46	0.05
2021/5/13 1:40	0.644	24.0	100.46	0.05
2021/5/13 1:50	0.644	24.0	100.46	0.06
2021/5/13 2:00	0.644	24.0	100.44	0.06

2021/5/13 2:10	0.644	24.0	100.44	0.05
2021/5/13 2:20	0.641	24.0	100.43	0.05
2021/5/13 2:30	0.641	24.0	100.44	0.05
2021/5/13 2:40	0.641	24.0	100.44	0.06
2021/5/13 2:50	0.641	24.0	100.44	0.05
2021/5/13 3:00	0.641	23.9	100.46	0.06
2021/5/13 3:10	0.641	23.9	100.46	0.05
2021/5/13 3:20	0.641	23.9	100.46	0.06
2021/5/13 3:30	0.644	23.9	100.47	0.05
2021/5/13 3:40	0.644	23.9	100.47	0.05
2021/5/13 3:50	0.644	23.9	100.47	0.05
2021/5/13 4:00	0.641	23.9	100.47	0.05
2021/5/13 4:10	0.645	23.8	100.49	0.05
2021/5/13 4:20	0.648	23.8	100.50	0.06
2021/5/13 4:30	0.648	23.8	100.51	0.06
2021/5/13 4:40	0.645	23.8	100.50	0.06
2021/5/13 4:50	0.645	23.8	100.51	0.06
2021/5/13 5:00	0.645	23.8	100.54	0.06
2021/5/13 5:10	0.641	23.8	100.54	0.06
2021/5/13 5:20	0.641	23.8	100.54	0.05
2021/5/13 5:30	0.638	23.8	100.54	0.05
2021/5/13 5:40	0.642	23.7	100.54	0.05
2021/5/13 5:50	0.642	23.7	100.56	0.05
2021/5/13 6:00	0.639	23.7	100.57	0.06
2021/5/13 6:10	0.639	23.7	100.59	0.05
2021/5/13 6:20	0.639	23.7	100.59	0.09
2021/5/13 6:30	0.638	23.8	100.60	0.16
2021/5/13 6:40	0.630	24.1	100.62	0.17
2021/5/13 6:50	0.630	24.2	100.62	0.17
2021/5/13 7:00	0.622	24.4	100.63	0.17
2021/5/13 7:10	0.622	24.5	100.63	0.17
2021/5/13 7:20	0.622	24.5	100.63	0.17
2021/5/13 7:30	0.618	24.6	100.63	0.17
2021/5/13 7:40	0.618	24.6	100.65	0.17
2021/5/13 7:50	0.621	24.6	100.65	0.16
2021/5/13 8:00	0.617	24.7	100.66	0.17
2021/5/13 8:10	0.617	24.7	100.66	0.16
2021/5/13 8:20	0.617	24.7	100.66	0.17
2021/5/13 8:30	0.617	24.7	100.65	0.16
2021/5/13 8:40	0.621	24.7	100.65	0.16
2021/5/13 8:50	0.621	24.7	100.65	0.17
2021/5/13 9:00	0.617	24.8	100.65	0.16
2021/5/13 9:10	0.617	24.8	100.66	0.17
2021/5/13 9:20	0.617	24.8	100.66	0.17
2021/5/13 9:30	0.617	24.8	100.65	0.16
2021/5/13 9:40	0.620	24.8	100.65	0.17
2021/5/13 9:50	0.616	24.9	100.66	0.16
2021/5/13 10:00	0.616	24.9	100.66	0.16
2021/5/13 10:10	0.620	24.9	100.68	0.17
2021/5/13 10:20	0.616	25.0	100.68	0.17
2021/5/13 10:30	0.616	25.0	100.68	0.17
2021/5/13 10:40	0.619	25.0	100.68	0.17
2021/5/13 10:50	0.619	25.0	100.68	0.16
2021/5/13 11:00	0.619	25.0	100.68	0.17
2021/5/13 11:10	0.615	25.1	100.68	0.16
2021/5/13 11:20	0.619	25.0	100.68	0.16

2021/5/13 11:30	0.622	25.0	100.66	0.16
2021/5/13 11:40	0.619	25.1	100.68	0.16
2021/5/13 11:50	0.619	25.1	100.68	0.16
2021/5/13 12:00	0.619	25.1	100.68	0.17
2021/5/13 12:10	0.619	25.1	100.66	0.17
2021/5/13 12:20	0.622	25.1	100.66	0.17
2021/5/13 12:30	0.622	25.1	100.66	0.17
2021/5/13 12:40	0.622	25.1	100.66	0.16
2021/5/13 12:50	0.625	25.1	100.66	0.16
2021/5/13 13:00	0.625	25.1	100.65	0.16
2021/5/13 13:10	0.625	25.1	100.63	0.16
2021/5/13 13:20	0.624	25.2	100.62	0.17
2021/5/13 13:30	0.627	25.2	100.62	0.15
2021/5/13 13:40	0.627	25.2	100.60	0.17
2021/5/13 13:50	0.627	25.2	100.60	0.16
2021/5/13 14:00	0.627	25.2	100.60	0.15
2021/5/13 14:10	0.627	25.2	100.60	0.16
2021/5/13 14:20	0.627	25.2	100.60	0.15
2021/5/13 14:30	0.627	25.2	100.60	0.15
2021/5/13 14:40	0.627	25.2	100.59	0.15
2021/5/13 14:50	0.627	25.2	100.57	0.15
2021/5/13 15:00	0.627	25.2	100.56	0.16
2021/5/13 15:10	0.627	25.3	100.54	0.15
2021/5/13 15:20	0.624	25.3	100.54	0.15
2021/5/13 15:30	0.627	25.3	100.53	0.15
2021/5/13 15:40	0.627	25.3	100.51	0.16
2021/5/13 15:50	0.623	25.4	100.51	0.14
2021/5/13 16:00	0.623	25.4	100.53	0.15
2021/5/13 16:10	0.623	25.4	100.53	0.15
2021/5/13 16:20	0.623	25.4	100.53	0.15
2021/5/13 16:30	0.623	25.4	100.53	0.15
2021/5/13 16:40	0.623	25.4	100.53	0.14
2021/5/13 16:50	0.623	25.4	100.51	0.15
2021/5/13 17:00	0.623	25.4	100.51	0.14
2021/5/13 17:10	0.623	25.4	100.51	0.14
2021/5/13 17:20	0.623	25.4	100.51	0.14
2021/5/13 17:30	0.623	25.4	100.50	0.15
2021/5/13 17:40	0.623	25.4	100.50	0.13
2021/5/13 17:50	0.623	25.4	100.50	0.15
2021/5/13 18:00	0.626	25.4	100.50	0.15
2021/5/13 18:10	0.626	25.4	100.51	0.14
2021/5/13 18:20	0.623	25.4	100.51	0.13
2021/5/13 18:30	0.624	25.3	100.51	0.06
2021/5/13 18:40	0.621	25.2	100.53	0.06
2021/5/13 18:50	0.625	25.0	100.54	0.06
2021/5/13 19:00	0.630	24.8	100.56	0.07
2021/5/13 19:10	0.630	24.7	100.57	0.07
2021/5/13 19:20	0.634	24.6	100.59	0.07
2021/5/13 19:30	0.631	24.6	100.59	0.07
2021/5/13 19:40	0.635	24.5	100.60	0.06
2021/5/13 19:50	0.635	24.5	100.62	0.07
2021/5/13 20:00	0.638	24.4	100.63	0.07
2021/5/13 20:10	0.638	24.4	100.63	0.06
2021/5/13 20:20	0.638	24.4	100.65	0.06
2021/5/13 20:30	0.639	24.3	100.65	0.06
2021/5/13 20:40	0.642	24.3	100.66	0.06

2021/5/13 20:50	0.642	24.3	100.68	0.06
2021/5/13 21:00	0.646	24.2	100.68	0.05
2021/5/13 21:10	0.646	24.2	100.69	0.05
2021/5/13 21:20	0.646	24.2	100.69	0.05
2021/5/13 21:30	0.646	24.2	100.70	0.06
2021/5/13 21:40	0.646	24.2	100.70	0.05
2021/5/13 21:50	0.650	24.1	100.70	0.05
2021/5/13 22:00	0.650	24.1	100.72	0.06
2021/5/13 22:10	0.650	24.1	100.70	0.06
2021/5/13 22:20	0.650	24.1	100.70	0.06
2021/5/13 22:30	0.654	24.0	100.72	0.06
2021/5/13 22:40	0.651	24.0	100.72	0.05
2021/5/13 22:50	0.651	24.0	100.73	0.05
2021/5/13 23:00	0.651	24.0	100.73	0.05
2021/5/13 23:10	0.651	24.0	100.72	0.05
2021/5/13 23:20	0.654	23.9	100.70	0.06
2021/5/13 23:30	0.654	23.9	100.70	0.05
2021/5/13 23:40	0.654	23.9	100.70	0.06
2021/5/13 23:50	0.651	23.9	100.70	0.05
2021/5/14 0:00	0.651	23.9	100.69	0.06
2021/5/14 0:10	0.655	23.8	100.69	0.06
2021/5/14 0:20	0.652	23.8	100.68	0.06
2021/5/14 0:30	0.652	23.8	100.68	0.05
2021/5/14 0:40	0.652	23.8	100.68	0.06
2021/5/14 0:50	0.652	23.8	100.68	0.05
2021/5/14 1:00	0.652	23.7	100.68	0.06
2021/5/14 1:10	0.652	23.7	100.68	0.06
2021/5/14 1:20	0.652	23.7	100.66	0.05
2021/5/14 1:30	0.649	23.7	100.68	0.05
2021/5/14 1:40	0.653	23.6	100.68	0.05
2021/5/14 1:50	0.649	23.6	100.69	0.05
2021/5/14 2:00	0.649	23.6	100.69	0.05
2021/5/14 2:10	0.646	23.6	100.69	0.05
2021/5/14 2:20	0.650	23.5	100.70	0.05
2021/5/14 2:30	0.650	23.5	100.70	0.05
2021/5/14 2:40	0.646	23.5	100.70	0.05
2021/5/14 2:50	0.646	23.5	100.70	0.04
2021/5/14 3:00	0.650	23.4	100.72	0.05
2021/5/14 3:10	0.647	23.4	100.72	0.04
2021/5/14 3:20	0.647	23.4	100.73	0.05
2021/5/14 3:30	0.647	23.4	100.73	0.04
2021/5/14 3:40	0.647	23.4	100.75	0.05
2021/5/14 3:50	0.647	23.3	100.73	0.04
2021/5/14 4:00	0.647	23.3	100.75	0.05
2021/5/14 4:10	0.647	23.3	100.75	0.04
2021/5/14 4:20	0.647	23.3	100.76	0.05
2021/5/14 4:30	0.647	23.2	100.78	0.05
2021/5/14 4:40	0.647	23.2	100.78	0.04
2021/5/14 4:50	0.647	23.2	100.78	0.05
2021/5/14 5:00	0.644	23.2	100.78	0.04
2021/5/14 5:10	0.647	23.2	100.79	0.05
2021/5/14 5:20	0.644	23.2	100.82	0.04
2021/5/14 5:30	0.648	23.1	100.85	0.04
2021/5/14 5:40	0.644	23.1	100.87	0.03
2021/5/14 5:50	0.644	23.1	100.87	0.03
2021/5/14 6:00	0.644	23.1	100.90	0.03

2021/5/14 6:10	0.648	23.0	100.90	0.04
2021/5/14 6:20	0.641	23.1	100.90	0.10
2021/5/14 6:30	0.637	23.3	100.91	0.16
2021/5/14 6:40	0.629	23.6	100.90	0.17
2021/5/14 6:50	0.628	23.8	100.88	0.17
2021/5/14 7:00	0.627	23.9	100.88	0.17
2021/5/14 7:10	0.627	24.0	100.90	0.17
2021/5/14 7:20	0.623	24.1	100.92	0.17
2021/5/14 7:30	0.623	24.2	100.94	0.16
2021/5/14 7:40	0.623	24.2	100.95	0.17
2021/5/14 7:50	0.626	24.2	100.95	0.18
2021/5/14 8:00	0.626	24.3	100.95	0.17
2021/5/14 8:10	0.629	24.3	100.95	0.18
2021/5/14 8:20	0.625	24.4	100.94	0.17
2021/5/14 8:30	0.629	24.4	100.94	0.17
2021/5/14 8:40	0.632	24.4	100.95	0.17
2021/5/14 8:50	0.628	24.5	100.95	0.17
2021/5/14 9:00	0.631	24.5	100.95	0.17
2021/5/14 9:10	0.635	24.5	100.94	0.17
2021/5/14 9:20	0.635	24.5	100.94	0.16
2021/5/14 9:30	0.634	24.6	100.94	0.17
2021/5/14 9:40	0.634	24.6	100.94	0.17
2021/5/14 9:50	0.633	24.7	100.92	0.17
2021/5/14 10:00	0.633	24.7	100.92	0.17
2021/5/14 10:10	0.633	24.8	100.91	0.17
2021/5/14 10:20	0.633	24.8	100.91	0.17
2021/5/14 10:30	0.632	24.9	100.92	0.17
2021/5/14 10:40	0.632	24.9	100.92	0.17
2021/5/14 10:50	0.629	25.0	100.91	0.17
2021/5/14 11:00	0.632	25.0	100.91	0.16
2021/5/14 11:10	0.635	25.0	100.91	0.16
2021/5/14 11:20	0.631	25.1	100.91	0.17
2021/5/14 11:30	0.634	25.1	100.90	0.17
2021/5/14 11:40	0.634	25.1	100.90	0.16
2021/5/14 11:50	0.634	25.2	100.90	0.16
2021/5/14 12:00	0.634	25.2	100.90	0.15
2021/5/14 12:10	0.634	25.2	100.90	0.15
2021/5/14 12:20	0.634	25.2	100.90	0.15
2021/5/14 12:30	0.637	25.2	100.88	0.15
2021/5/14 12:40	0.637	25.2	100.87	0.17
2021/5/14 12:50	0.640	25.2	100.87	0.15
2021/5/14 13:00	0.640	25.2	100.88	0.17
2021/5/14 13:10	0.636	25.3	100.90	0.16
2021/5/14 13:20	0.639	25.3	100.90	0.16
2021/5/14 13:30	0.639	25.3	100.90	0.17
2021/5/14 13:40	0.642	25.3	100.88	0.17
2021/5/14 13:50	0.645	25.3	100.85	0.15
2021/5/14 14:00	0.645	25.3	100.85	0.16
2021/5/14 14:10	0.645	25.3	100.84	0.15
2021/5/14 14:20	0.648	25.3	100.81	0.15
2021/5/14 14:30	0.645	25.4	100.79	0.17
2021/5/14 14:40	0.652	25.3	100.79	0.14
2021/5/14 14:50	0.648	25.4	100.79	0.15
2021/5/14 15:00	0.652	25.3	100.78	0.16
2021/5/14 15:10	0.655	25.3	100.78	0.14
2021/5/14 15:20	0.655	25.3	100.78	0.16

2021/5/14 15:30	0.654	25.4	100.78	0.14
2021/5/14 15:40	0.654	25.4	100.78	0.15
2021/5/14 15:50	0.654	25.4	100.79	0.16
2021/5/14 16:00	0.657	25.4	100.81	0.14
2021/5/14 16:10	0.654	25.4	100.79	0.15
2021/5/14 16:20	0.657	25.4	100.82	0.14
2021/5/14 16:30	0.657	25.4	100.82	0.15
2021/5/14 16:40	0.657	25.4	100.84	0.15
2021/5/14 16:50	0.660	25.4	100.85	0.15
2021/5/14 17:00	0.660	25.4	100.87	0.15
2021/5/14 17:10	0.660	25.4	100.87	0.16
2021/5/14 17:20	0.664	25.3	100.87	0.15
2021/5/14 17:30	0.663	25.4	100.87	0.14
2021/5/14 17:40	0.663	25.4	100.87	0.14
2021/5/14 17:50	0.659	25.5	100.87	0.14
2021/5/14 18:00	0.662	25.5	100.88	0.14
2021/5/14 18:10	0.663	25.4	100.90	0.15
2021/5/14 18:20	0.663	25.4	100.90	0.13
2021/5/14 18:30	0.668	25.2	100.91	0.06
2021/5/14 18:40	0.666	25.1	100.91	0.07
2021/5/14 18:50	0.666	25.0	100.91	0.06
2021/5/14 19:00	0.671	24.8	100.92	0.06
2021/5/14 19:10	0.672	24.7	100.91	0.06
2021/5/14 19:20	0.669	24.7	100.91	0.05
2021/5/14 19:30	0.670	24.6	100.91	0.05
2021/5/14 19:40	0.674	24.5	100.91	0.06
2021/5/14 19:50	0.674	24.5	100.90	0.06
2021/5/14 20:00	0.670	24.5	100.91	0.06
2021/5/14 20:10	0.674	24.4	100.91	0.07
2021/5/14 20:20	0.671	24.4	100.91	0.07
2021/5/14 20:30	0.671	24.4	100.90	0.07
2021/5/14 20:40	0.675	24.3	100.91	0.07
2021/5/14 20:50	0.672	24.3	100.90	0.06
2021/5/14 21:00	0.672	24.3	100.88	0.07
2021/5/14 21:10	0.672	24.3	100.90	0.07
2021/5/14 21:20	0.672	24.3	100.88	0.07
2021/5/14 21:30	0.676	24.2	100.90	0.07
2021/5/14 21:40	0.673	24.2	100.88	0.07
2021/5/14 21:50	0.673	24.2	100.87	0.06
2021/5/14 22:00	0.673	24.2	100.87	0.08
2021/5/14 22:10	0.673	24.2	100.87	0.08
2021/5/14 22:20	0.669	24.2	100.87	0.07
2021/5/14 22:30	0.669	24.2	100.84	0.07
2021/5/14 22:40	0.673	24.1	100.82	0.08
2021/5/14 22:50	0.670	24.1	100.79	0.07
2021/5/14 23:00	0.670	24.1	100.79	0.07
2021/5/14 23:10	0.670	24.1	100.79	0.08
2021/5/14 23:20	0.670	24.1	100.79	0.08
2021/5/14 23:30	0.670	24.1	100.79	0.08
2021/5/14 23:40	0.667	24.1	100.79	0.08
2021/5/14 23:50	0.667	24.1	100.78	0.08
2021/5/15 0:00	0.667	24.1	100.78	0.08
2021/5/15 0:10	0.663	24.1	100.76	0.08
2021/5/15 0:20	0.663	24.1	100.75	0.08
2021/5/15 0:30	0.667	24.0	100.72	0.08
2021/5/15 0:40	0.664	24.0	100.72	0.08

2021/5/15 0:50	0.664	24.0	100.73	0.09
2021/5/15 1:00	0.664	24.0	100.72	0.09
2021/5/15 1:10	0.661	24.0	100.70	0.09
2021/5/15 1:20	0.661	24.0	100.70	0.08
2021/5/15 1:30	0.661	24.0	100.72	0.08
2021/5/15 1:40	0.657	24.0	100.72	0.08
2021/5/15 1:50	0.657	24.0	100.73	0.10
2021/5/15 2:00	0.657	24.0	100.73	0.08
2021/5/15 2:10	0.661	23.9	100.72	0.08
2021/5/15 2:20	0.658	23.9	100.70	0.08
2021/5/15 2:30	0.658	23.9	100.70	0.08
2021/5/15 2:40	0.654	23.9	100.70	0.09
2021/5/15 2:50	0.654	23.9	100.70	0.08
2021/5/15 3:00	0.654	23.9	100.70	0.08
2021/5/15 3:10	0.651	23.9	100.70	0.08
2021/5/15 3:20	0.655	23.8	100.70	0.07
2021/5/15 3:30	0.655	23.8	100.70	0.07
2021/5/15 3:40	0.652	23.8	100.69	0.07
2021/5/15 3:50	0.652	23.8	100.68	0.08
2021/5/15 4:00	0.648	23.8	100.66	0.08
2021/5/15 4:10	0.652	23.8	100.65	0.08
2021/5/15 4:20	0.648	23.8	100.63	0.07
2021/5/15 4:30	0.648	23.8	100.62	0.08
2021/5/15 4:40	0.652	23.7	100.62	0.09
2021/5/15 4:50	0.652	23.7	100.60	0.07
2021/5/15 5:00	0.652	23.7	100.57	0.06
2021/5/15 5:10	0.652	23.7	100.57	0.07
2021/5/15 5:20	0.652	23.7	100.57	0.08
2021/5/15 5:30	0.649	23.7	100.57	0.09
2021/5/15 5:40	0.649	23.7	100.59	0.08
2021/5/15 5:50	0.649	23.7	100.59	0.07
2021/5/15 6:00	0.649	23.7	100.57	0.07
2021/5/15 6:10	0.649	23.7	100.57	0.08
2021/5/15 6:20	0.649	23.7	100.57	0.10
2021/5/15 6:30	0.648	23.8	100.57	0.17
2021/5/15 6:40	0.643	24.1	100.59	0.18
2021/5/15 6:50	0.643	24.1	100.59	0.16
2021/5/15 7:00	0.643	24.2	100.59	0.15
2021/5/15 7:10	0.643	24.2	100.57	0.15
2021/5/15 7:20	0.643	24.2	100.56	0.15
2021/5/15 7:30	0.646	24.2	100.57	0.15
2021/5/15 7:40	0.646	24.2	100.57	0.15
2021/5/15 7:50	0.642	24.3	100.57	0.13
2021/5/15 8:00	0.646	24.3	100.57	0.14
2021/5/15 8:10	0.646	24.3	100.56	0.15
2021/5/15 8:20	0.646	24.3	100.56	0.15
2021/5/15 8:30	0.646	24.3	100.54	0.14
2021/5/15 8:40	0.649	24.3	100.54	0.15
2021/5/15 8:50	0.645	24.4	100.54	0.15
2021/5/15 9:00	0.645	24.4	100.53	0.15
2021/5/15 9:10	0.648	24.4	100.53	0.16
2021/5/15 9:20	0.648	24.4	100.54	0.15
2021/5/15 9:30	0.648	24.4	100.54	0.15
2021/5/15 9:40	0.648	24.4	100.54	0.14
2021/5/15 9:50	0.648	24.5	100.53	0.15
2021/5/15 10:00	0.648	24.5	100.51	0.15

2021/5/15 10:10	0.651	24.5	100.50	0.15
2021/5/15 10:20	0.651	24.5	100.47	0.14
2021/5/15 10:30	0.650	24.6	100.51	0.14
2021/5/15 10:40	0.653	24.6	100.56	0.14
2021/5/15 10:50	0.653	24.6	100.56	0.14
2021/5/15 11:00	0.650	24.7	100.54	0.14
2021/5/15 11:10	0.650	24.7	100.56	0.14
2021/5/15 11:20	0.650	24.7	100.57	0.14
2021/5/15 11:30	0.653	24.7	100.60	0.13
2021/5/15 11:40	0.653	24.7	100.62	0.13
2021/5/15 11:50	0.653	24.7	100.62	0.14
2021/5/15 12:00	0.656	24.7	100.62	0.15
2021/5/15 12:10	0.659	24.7	100.62	0.14
2021/5/15 12:20	0.662	24.7	100.65	0.08
2021/5/15 12:30	0.658	24.8	100.66	0.07
2021/5/15 12:40	0.658	24.9	100.62	0.08
2021/5/15 12:50	0.658	24.9	100.60	0.06
2021/5/15 13:00	0.658	24.9	100.60	0.08
2021/5/15 13:10	0.661	24.9	100.57	0.07
2021/5/15 13:20	0.661	24.9	100.56	0.07
2021/5/15 13:30	0.661	24.9	100.53	0.07
2021/5/15 13:40	0.661	24.9	100.51	0.07
2021/5/15 13:50	0.664	24.9	100.54	0.07
2021/5/15 14:00	0.664	24.9	100.54	0.08
2021/5/15 14:10	0.664	24.9	100.51	0.07
2021/5/15 14:20	0.664	24.9	100.49	0.08
2021/5/15 14:30	0.660	25.0	100.53	0.13
2021/5/15 14:40	0.663	25.0	100.53	0.14
2021/5/15 14:50	0.659	25.1	100.53	0.15
2021/5/15 15:00	0.655	25.2	100.51	0.12
2021/5/15 15:10	0.659	25.2	100.50	0.13
2021/5/15 15:20	0.655	25.3	100.51	0.13
2021/5/15 15:30	0.654	25.4	100.50	0.13
2021/5/15 15:40	0.654	25.4	100.47	0.13
2021/5/15 15:50	0.654	25.4	100.49	0.13
2021/5/15 16:00	0.657	25.4	100.49	0.12
2021/5/15 16:10	0.657	25.4	100.49	0.12
2021/5/15 16:20	0.653	25.5	100.49	0.12
2021/5/15 16:30	0.653	25.5	100.50	0.13
2021/5/15 16:40	0.656	25.5	100.50	0.12
2021/5/15 16:50	0.656	25.5	100.50	0.11
2021/5/15 17:00	0.652	25.6	100.50	0.11
2021/5/15 17:10	0.652	25.6	100.50	0.12
2021/5/15 17:20	0.652	25.6	100.49	0.12
2021/5/15 17:30	0.652	25.6	100.47	0.12
2021/5/15 17:40	0.655	25.6	100.47	0.11
2021/5/15 17:50	0.655	25.6	100.46	0.11
2021/5/15 18:00	0.655	25.6	100.43	0.11
2021/5/15 18:10	0.655	25.6	100.40	0.11
2021/5/15 18:20	0.655	25.6	100.41	0.10
2021/5/15 18:30	0.658	25.6	100.43	0.08
2021/5/15 18:40	0.656	25.5	100.43	0.06
2021/5/15 18:50	0.658	25.3	100.43	0.05
2021/5/15 19:00	0.659	25.1	100.44	0.03
2021/5/15 19:10	0.668	24.8	100.47	0.04
2021/5/15 19:20	0.669	24.7	100.46	0.03

2021/5/15 19:30	0.674	24.5	100.46	0.03
2021/5/15 19:40	0.674	24.4	100.46	0.03
2021/5/15 19:50	0.675	24.3	100.46	0.03
2021/5/15 20:00	0.679	24.2	100.47	0.03
2021/5/15 20:10	0.676	24.2	100.49	0.03
2021/5/15 20:20	0.680	24.1	100.49	0.03
2021/5/15 20:30	0.677	24.1	100.49	0.03
2021/5/15 20:40	0.677	24.1	100.49	0.03
2021/5/15 20:50	0.681	24.0	100.50	0.03
2021/5/15 21:00	0.677	24.0	100.50	0.03
2021/5/15 21:10	0.677	24.0	100.50	0.03
2021/5/15 21:20	0.677	24.0	100.51	0.03
2021/5/15 21:30	0.681	23.9	100.51	0.02
2021/5/15 21:40	0.678	23.9	100.51	0.02
2021/5/15 21:50	0.678	23.9	100.53	0.03
2021/5/15 22:00	0.678	23.9	100.53	0.03
2021/5/15 22:10	0.678	23.9	100.53	0.03
2021/5/15 22:20	0.682	23.8	100.53	0.03
2021/5/15 22:30	0.679	23.8	100.53	0.03
2021/5/15 22:40	0.682	23.8	100.53	0.02
2021/5/15 22:50	0.679	23.8	100.51	0.03
2021/5/15 23:00	0.679	23.8	100.54	0.03
2021/5/15 23:10	0.679	23.8	100.54	0.03
2021/5/15 23:20	0.679	23.8	100.54	0.03
2021/5/15 23:30	0.679	23.8	100.56	0.03
2021/5/15 23:40	0.683	23.7	100.56	0.03
2021/5/15 23:50	0.679	23.7	100.57	0.03
2021/5/16 0:00	0.683	23.7	100.56	0.03
2021/5/16 0:10	0.679	23.7	100.56	0.02
2021/5/16 0:20	0.679	23.7	100.56	0.02
2021/5/16 0:30	0.679	23.7	100.56	0.02
2021/5/16 0:40	0.679	23.7	100.57	0.03
2021/5/16 0:50	0.679	23.7	100.57	0.02
2021/5/16 1:00	0.684	23.6	100.59	0.02
2021/5/16 1:10	0.684	23.6	100.59	0.02
2021/5/16 1:20	0.684	23.6	100.59	0.02
2021/5/16 1:30	0.680	23.6	100.59	0.03
2021/5/16 1:40	0.680	23.6	100.60	0.02
2021/5/16 1:50	0.680	23.6	100.60	0.02
2021/5/16 2:00	0.680	23.6	100.62	0.02
2021/5/16 2:10	0.681	23.5	100.62	0.02
2021/5/16 2:20	0.681	23.5	100.60	0.03
2021/5/16 2:30	0.681	23.5	100.60	0.02
2021/5/16 2:40	0.681	23.5	100.60	0.02
2021/5/16 2:50	0.681	23.5	100.60	0.02
2021/5/16 3:00	0.681	23.5	100.59	0.02
2021/5/16 3:10	0.677	23.5	100.57	0.02
2021/5/16 3:20	0.677	23.5	100.59	0.02
2021/5/16 3:30	0.677	23.5	100.59	0.02
2021/5/16 3:40	0.677	23.5	100.60	0.02
2021/5/16 3:50	0.681	23.4	100.60	0.02
2021/5/16 4:00	0.681	23.4	100.60	0.02
2021/5/16 4:10	0.681	23.4	100.60	0.02
2021/5/16 4:20	0.681	23.4	100.59	0.02
2021/5/16 4:30	0.681	23.4	100.59	0.02
2021/5/16 4:40	0.681	23.4	100.57	0.02

2021/5/16 4:50	0.681	23.4	100.59	0.02
2021/5/16 5:00	0.681	23.4	100.59	0.02
2021/5/16 5:10	0.686	23.3	100.59	0.02
2021/5/16 5:20	0.686	23.3	100.60	0.03
2021/5/16 5:30	0.686	23.3	100.60	0.02
2021/5/16 5:40	0.682	23.3	100.59	0.03
2021/5/16 5:50	0.682	23.3	100.60	0.03
2021/5/16 6:00	0.682	23.3	100.57	0.03
2021/5/16 6:10	0.686	23.2	100.56	0.03
2021/5/16 6:20	0.690	23.2	100.56	0.05
2021/5/16 6:30	0.685	23.4	100.56	0.11
2021/5/16 6:40	0.679	23.7	100.54	0.13
2021/5/16 6:50	0.674	24.0	100.56	0.15
2021/5/16 7:00	0.673	24.2	100.54	0.16
2021/5/16 7:10	0.668	24.4	100.57	0.16
2021/5/16 7:20	0.667	24.5	100.57	0.17
2021/5/16 7:30	0.666	24.6	100.56	0.16
2021/5/16 7:40	0.666	24.7	100.57	0.17
2021/5/16 7:50	0.666	24.7	100.57	0.16
2021/5/16 8:00	0.665	24.8	100.60	0.16
2021/5/16 8:10	0.668	24.8	100.59	0.15
2021/5/16 8:20	0.664	24.9	100.59	0.17
2021/5/16 8:30	0.667	24.9	100.59	0.16
2021/5/16 8:40	0.667	24.9	100.60	0.17
2021/5/16 8:50	0.670	24.9	100.59	0.16
2021/5/16 9:00	0.670	24.9	100.57	0.16
2021/5/16 9:10	0.670	24.9	100.59	0.17
2021/5/16 9:20	0.670	25.0	100.57	0.16
2021/5/16 9:30	0.670	25.0	100.56	0.17
2021/5/16 9:40	0.670	25.0	100.54	0.17
2021/5/16 9:50	0.673	25.0	100.54	0.15
2021/5/16 10:00	0.669	25.1	100.53	0.14
2021/5/16 10:10	0.669	25.1	100.53	0.15
2021/5/16 10:20	0.668	25.2	100.51	0.15
2021/5/16 10:30	0.668	25.2	100.51	0.16
2021/5/16 10:40	0.668	25.2	100.50	0.15
2021/5/16 10:50	0.667	25.3	100.47	0.14
2021/5/16 11:00	0.667	25.3	100.44	0.15
2021/5/16 11:10	0.663	25.4	100.40	0.15
2021/5/16 11:20	0.667	25.3	100.41	0.17
2021/5/16 11:30	0.667	25.3	100.38	0.16
2021/5/16 11:40	0.670	25.3	100.34	0.16
2021/5/16 11:50	0.666	25.4	100.32	0.15
2021/5/16 12:00	0.666	25.4	100.31	0.15
2021/5/16 12:10	0.669	25.4	100.29	0.14
2021/5/16 12:20	0.669	25.4	100.28	0.15
2021/5/16 12:30	0.669	25.4	100.25	0.14
2021/5/16 12:40	0.666	25.4	100.22	0.15
2021/5/16 12:50	0.666	25.4	100.24	0.14
2021/5/16 13:00	0.666	25.4	100.24	0.15
2021/5/16 13:10	0.662	25.5	100.24	0.15
2021/5/16 13:20	0.662	25.5	100.22	0.15
2021/5/16 13:30	0.662	25.5	100.21	0.14
2021/5/16 13:40	0.662	25.5	100.18	0.14
2021/5/16 13:50	0.662	25.5	100.16	0.15
2021/5/16 14:00	0.662	25.5	100.16	0.15

2021/5/16 14:10	0.662	25.5	100.15	0.14
2021/5/16 14:20	0.662	25.5	100.13	0.14
2021/5/16 14:30	0.662	25.5	100.10	0.15
2021/5/16 14:40	0.665	25.5	100.09	0.13
2021/5/16 14:50	0.661	25.6	100.06	0.15
2021/5/16 15:00	0.661	25.6	100.03	0.15
2021/5/16 15:10	0.661	25.6	100.02	0.15
2021/5/16 15:20	0.661	25.6	100.00	0.15
2021/5/16 15:30	0.661	25.6	99.97	0.15
2021/5/16 15:40	0.664	25.6	99.97	0.15
2021/5/16 15:50	0.664	25.6	99.97	0.16
2021/5/16 16:00	0.664	25.6	100.03	0.15
2021/5/16 16:10	0.664	25.6	100.07	0.15
2021/5/16 16:20	0.664	25.6	100.12	0.14
2021/5/16 16:30	0.661	25.7	100.15	0.14
2021/5/16 16:40	0.661	25.7	100.15	0.14
2021/5/16 16:50	0.664	25.7	100.21	0.13
2021/5/16 17:00	0.660	25.8	100.21	0.13
2021/5/16 17:10	0.660	25.8	100.24	0.13
2021/5/16 17:20	0.663	25.8	100.22	0.12
2021/5/16 17:30	0.663	25.8	100.24	0.12
2021/5/16 17:40	0.660	25.8	100.24	0.11
2021/5/16 17:50	0.660	25.8	100.27	0.11
2021/5/16 18:00	0.660	25.8	100.28	0.12
2021/5/16 18:10	0.657	25.8	100.29	0.12
2021/5/16 18:20	0.660	25.8	100.32	0.11
2021/5/16 18:30	0.657	25.8	100.35	0.07
2021/5/16 18:40	0.654	25.7	100.37	0.08
2021/5/16 18:50	0.656	25.5	100.40	0.07
2021/5/16 19:00	0.658	25.3	100.43	0.06
2021/5/16 19:10	0.655	25.2	100.47	0.06
2021/5/16 19:20	0.657	25.0	100.51	0.05
2021/5/16 19:30	0.658	24.8	100.54	0.05
2021/5/16 19:40	0.656	24.7	100.56	0.04
2021/5/16 19:50	0.653	24.6	100.57	0.05
2021/5/16 20:00	0.654	24.5	100.60	0.04
2021/5/16 20:10	0.648	24.5	100.62	0.04
2021/5/16 20:20	0.645	24.4	100.63	0.04
2021/5/16 20:30	0.642	24.3	100.62	0.04
2021/5/16 20:40	0.639	24.3	100.63	0.04
2021/5/16 20:50	0.639	24.2	100.65	0.04
2021/5/16 21:00	0.633	24.2	100.65	0.04
2021/5/16 21:10	0.637	24.1	100.66	0.03
2021/5/16 21:20	0.633	24.1	100.68	0.04
2021/5/16 21:30	0.630	24.1	100.68	0.04
2021/5/16 21:40	0.630	24.0	100.69	0.04
2021/5/16 21:50	0.627	24.0	100.70	0.04
2021/5/16 22:00	0.624	24.0	100.73	0.04
2021/5/16 22:10	0.620	24.0	100.73	0.03
2021/5/16 22:20	0.624	23.9	100.73	0.03
2021/5/16 22:30	0.621	23.9	100.73	0.03
2021/5/16 22:40	0.617	23.9	100.72	0.03
2021/5/16 22:50	0.614	23.9	100.72	0.03
2021/5/16 23:00	0.618	23.8	100.72	0.03
2021/5/16 23:10	0.614	23.8	100.73	0.03
2021/5/16 23:20	0.611	23.8	100.73	0.03

2021/5/16 23:30	0.611	23.8	100.73	0.03
2021/5/16 23:40	0.611	23.7	100.75	0.03
2021/5/16 23:50	0.611	23.7	100.73	0.03
2021/5/17 0:00	0.608	23.7	100.73	0.03
2021/5/17 0:10	0.608	23.6	100.75	0.03
2021/5/17 0:20	0.608	23.6	100.75	0.02
2021/5/17 0:30	0.608	23.6	100.75	0.03
2021/5/17 0:40	0.608	23.6	100.75	0.02
2021/5/17 0:50	0.608	23.5	100.76	0.03
2021/5/17 1:00	0.608	23.5	100.78	0.03
2021/5/17 1:10	0.608	23.5	100.78	0.03
2021/5/17 1:20	0.605	23.5	100.79	0.03
2021/5/17 1:30	0.605	23.5	100.79	0.03
2021/5/17 1:40	0.608	23.4	100.82	0.03
2021/5/17 1:50	0.608	23.4	100.82	0.03
2021/5/17 2:00	0.605	23.4	100.82	0.03
2021/5/17 2:10	0.605	23.4	100.82	0.03
2021/5/17 2:20	0.601	23.4	100.81	0.03
2021/5/17 2:30	0.605	23.3	100.81	0.03
2021/5/17 2:40	0.605	23.3	100.81	0.03
2021/5/17 2:50	0.602	23.3	100.82	0.03
2021/5/17 3:00	0.602	23.3	100.82	0.03
2021/5/17 3:10	0.605	23.2	100.82	0.03
2021/5/17 3:20	0.602	23.2	100.84	0.03
2021/5/17 3:30	0.602	23.2	100.87	0.04
2021/5/17 3:40	0.602	23.2	100.88	0.03
2021/5/17 3:50	0.602	23.2	100.90	0.03
2021/5/17 4:00	0.602	23.1	100.90	0.03
2021/5/17 4:10	0.602	23.1	100.90	0.03
2021/5/17 4:20	0.602	23.1	100.90	0.03
2021/5/17 4:30	0.598	23.1	100.90	0.04
2021/5/17 4:40	0.602	23.0	100.90	0.04
2021/5/17 4:50	0.602	23.0	100.90	0.04
2021/5/17 5:00	0.598	23.0	100.88	0.04
2021/5/17 5:10	0.602	22.9	100.88	0.05
2021/5/17 5:20	0.602	22.9	100.91	0.04
2021/5/17 5:30	0.602	22.9	100.91	0.04
2021/5/17 5:40	0.598	22.9	100.91	0.03
2021/5/17 5:50	0.598	22.9	100.92	0.03
2021/5/17 6:00	0.598	22.9	100.92	0.04
2021/5/17 6:10	0.602	22.8	100.94	0.04
2021/5/17 6:20	0.602	22.8	100.97	0.04
2021/5/17 6:30	0.598	23.0	100.98	0.11
2021/5/17 6:40	0.595	23.3	101.00	0.10
2021/5/17 6:50	0.591	23.6	100.98	0.09
2021/5/17 7:00	0.591	23.8	100.95	0.10
2021/5/17 7:10	0.587	24.0	100.98	0.11
2021/5/17 7:20	0.583	24.2	101.00	0.11
2021/5/17 7:30	0.580	24.3	101.04	0.11
2021/5/17 7:40	0.579	24.4	101.06	0.13
2021/5/17 7:50	0.579	24.5	101.06	0.13
2021/5/17 8:00	0.576	24.6	101.07	0.14
2021/5/17 8:10	0.579	24.6	101.06	0.13
2021/5/17 8:20	0.579	24.6	101.07	0.13
2021/5/17 8:30	0.579	24.7	101.09	0.16
2021/5/17 8:40	0.579	24.7	101.09	0.17

2021/5/17 8:50	0.582	24.7	101.09	0.16
2021/5/17 9:00	0.582	24.7	101.09	0.17
2021/5/17 9:10	0.585	24.7	101.09	0.18
2021/5/17 9:20	0.585	24.7	101.10	0.18
2021/5/17 9:30	0.588	24.7	101.12	0.18
2021/5/17 9:40	0.592	24.7	101.13	0.18
2021/5/17 9:50	0.588	24.8	101.13	0.18
2021/5/17 10:00	0.591	24.8	101.13	0.18
2021/5/17 10:10	0.591	24.8	101.13	0.17
2021/5/17 10:20	0.591	24.8	101.12	0.18
2021/5/17 10:30	0.591	24.8	101.12	0.17
2021/5/17 10:40	0.585	24.9	101.12	0.17
2021/5/17 10:50	0.585	24.9	101.12	0.16
2021/5/17 11:00	0.585	24.9	101.10	0.17
2021/5/17 11:10	0.581	25.0	101.09	0.17
2021/5/17 11:20	0.581	25.0	101.09	0.17
2021/5/17 11:30	0.581	25.0	101.07	0.17
2021/5/17 11:40	0.578	25.1	101.07	0.16
2021/5/17 11:50	0.578	25.1	101.07	0.15
2021/5/17 12:00	0.574	25.2	101.07	0.16
2021/5/17 12:10	0.574	25.2	101.07	0.16
2021/5/17 12:20	0.571	25.2	101.06	0.16
2021/5/17 12:30	0.571	25.2	101.06	0.16
2021/5/17 12:40	0.571	25.2	101.04	0.16
2021/5/17 12:50	0.571	25.2	101.03	0.15
2021/5/17 13:00	0.571	25.2	101.00	0.15
2021/5/17 13:10	0.571	25.2	101.00	0.16
2021/5/17 13:20	0.571	25.2	101.00	0.16
2021/5/17 13:30	0.571	25.2	100.98	0.15
2021/5/17 13:40	0.568	25.3	100.97	0.15
2021/5/17 13:50	0.568	25.3	100.97	0.16
2021/5/17 14:00	0.565	25.3	100.94	0.16
2021/5/17 14:10	0.561	25.4	100.94	0.15
2021/5/17 14:20	0.561	25.4	100.94	0.13
2021/5/17 14:30	0.561	25.4	100.94	0.15
2021/5/17 14:40	0.558	25.4	100.91	0.14
2021/5/17 14:50	0.558	25.4	100.91	0.15
2021/5/17 15:00	0.555	25.4	100.88	0.13
2021/5/17 15:10	0.555	25.4	100.90	0.14
2021/5/17 15:20	0.552	25.4	100.92	0.14
2021/5/17 15:30	0.552	25.4	100.92	0.13
2021/5/17 15:40	0.549	25.4	100.90	0.13
2021/5/17 15:50	0.549	25.4	100.88	0.14
2021/5/17 16:00	0.546	25.4	100.87	0.14
2021/5/17 16:10	0.546	25.4	100.85	0.14
2021/5/17 16:20	0.549	25.4	100.88	0.13
2021/5/17 16:30	0.549	25.4	100.87	0.13
2021/5/17 16:40	0.549	25.4	100.88	0.14
2021/5/17 16:50	0.552	25.4	100.90	0.13
2021/5/17 17:00	0.552	25.4	100.88	0.13
2021/5/17 17:10	0.555	25.4	100.90	0.15
2021/5/17 17:20	0.555	25.4	100.91	0.12
2021/5/17 17:30	0.558	25.4	100.91	0.13
2021/5/17 17:40	0.558	25.4	100.88	0.13
2021/5/17 17:50	0.558	25.4	100.87	0.14
2021/5/17 18:00	0.561	25.4	100.85	0.15

2021/5/17 18:10	0.561	25.5	100.84	0.14
2021/5/17 18:20	0.568	25.4	100.84	0.10
2021/5/17 18:30	0.568	25.4	100.82	0.05
2021/5/17 18:40	0.565	25.3	100.81	0.05
2021/5/17 18:50	0.568	25.1	100.81	0.05
2021/5/17 19:00	0.572	24.9	100.82	0.05
2021/5/17 19:10	0.576	24.7	100.82	0.05
2021/5/17 19:20	0.576	24.6	100.81	0.04
2021/5/17 19:30	0.576	24.5	100.79	0.05
2021/5/17 19:40	0.580	24.3	100.79	0.04
2021/5/17 19:50	0.583	24.2	100.79	0.04
2021/5/17 20:00	0.580	24.2	100.78	0.04
2021/5/17 20:10	0.583	24.1	100.78	0.03
2021/5/17 20:20	0.587	24.0	100.78	0.03
2021/5/17 20:30	0.587	24.0	100.78	0.03
2021/5/17 20:40	0.590	23.9	100.78	0.03
2021/5/17 20:50	0.590	23.9	100.78	0.03
2021/5/17 21:00	0.587	23.9	100.76	0.03
2021/5/17 21:10	0.591	23.8	100.75	0.03
2021/5/17 21:20	0.591	23.8	100.75	0.03
2021/5/17 21:30	0.591	23.8	100.75	0.03
2021/5/17 21:40	0.587	23.8	100.75	0.03
2021/5/17 21:50	0.587	23.8	100.76	0.03
2021/5/17 22:00	0.587	23.8	100.76	0.03
2021/5/17 22:10	0.587	23.7	100.76	0.03
2021/5/17 22:20	0.587	23.7	100.76	0.03
2021/5/17 22:30	0.584	23.7	100.76	0.03
2021/5/17 22:40	0.584	23.7	100.78	0.03
2021/5/17 22:50	0.580	23.7	100.79	0.04
2021/5/17 23:00	0.577	23.7	100.79	0.04
2021/5/17 23:10	0.577	23.7	100.78	0.04
2021/5/17 23:20	0.574	23.7	100.76	0.04
2021/5/17 23:30	0.570	23.7	100.75	0.04
2021/5/17 23:40	0.570	23.7	100.75	0.04
2021/5/17 23:50	0.570	23.6	100.75	0.04
2021/5/18 0:00	0.570	23.6	100.75	0.04
2021/5/18 0:10	0.567	23.6	100.75	0.04
2021/5/18 0:20	0.567	23.6	100.75	0.04
2021/5/18 0:30	0.563	23.6	100.76	0.04
2021/5/18 0:40	0.563	23.6	100.78	0.04
2021/5/18 0:50	0.560	23.6	100.79	0.04
2021/5/18 1:00	0.556	23.6	100.81	0.04
2021/5/18 1:10	0.556	23.6	100.81	0.04
2021/5/18 1:20	0.556	23.5	100.79	0.04
2021/5/18 1:30	0.556	23.5	100.78	0.04
2021/5/18 1:40	0.556	23.5	100.78	0.04
2021/5/18 1:50	0.556	23.5	100.76	0.04
2021/5/18 2:00	0.560	23.4	100.75	0.04
2021/5/18 2:10	0.563	23.4	100.72	0.04
2021/5/18 2:20	0.563	23.4	100.72	0.04
2021/5/18 2:30	0.563	23.4	100.69	0.04
2021/5/18 2:40	0.570	23.3	100.68	0.03
2021/5/18 2:50	0.570	23.3	100.68	0.04
2021/5/18 3:00	0.570	23.3	100.66	0.03
2021/5/18 3:10	0.574	23.3	100.65	0.04
2021/5/18 3:20	0.577	23.2	100.62	0.03

2021/5/18 3:30	0.577	23.2	100.60	0.04
2021/5/18 3:40	0.581	23.2	100.59	0.04
2021/5/18 3:50	0.581	23.2	100.57	0.04
2021/5/18 4:00	0.581	23.2	100.56	0.04
2021/5/18 4:10	0.584	23.2	100.51	0.04
2021/5/18 4:20	0.584	23.2	100.47	0.04
2021/5/18 4:30	0.588	23.2	100.44	0.04
2021/5/18 4:40	0.591	23.1	100.41	0.04
2021/5/18 4:50	0.591	23.1	100.37	0.04
2021/5/18 5:00	0.591	23.1	100.31	0.04
2021/5/18 5:10	0.595	23.1	100.25	0.04
2021/5/18 5:20	0.595	23.1	100.22	0.04
2021/5/18 5:30	0.598	23.0	100.25	0.04
2021/5/18 5:40	0.598	23.0	100.25	0.04
2021/5/18 5:50	0.602	23.0	100.25	0.04
2021/5/18 6:00	0.602	23.0	100.24	0.04
2021/5/18 6:10	0.602	23.0	100.21	0.04
2021/5/18 6:20	0.606	23.0	100.19	0.06
2021/5/18 6:30	0.609	23.1	100.19	0.11
2021/5/18 6:40	0.605	23.4	100.18	0.12
2021/5/18 6:50	0.608	23.6	100.15	0.11
2021/5/18 7:00	0.604	23.9	100.15	0.11
2021/5/18 7:10	0.600	24.1	100.18	0.12
2021/5/18 7:20	0.603	24.2	100.16	0.14
2021/5/18 7:30	0.603	24.3	100.10	0.13
2021/5/18 7:40	0.602	24.4	100.07	0.13
2021/5/18 7:50	0.602	24.5	100.10	0.12
2021/5/18 8:00	0.605	24.5	100.12	0.13
2021/5/18 8:10	0.605	24.5	100.07	0.13
2021/5/18 8:20	0.605	24.6	100.03	0.12
2021/5/18 8:30	0.608	24.6	100.02	0.12
2021/5/18 8:40	0.608	24.6	100.03	0.12
2021/5/18 8:50	0.608	24.6	100.03	0.13
2021/5/18 9:00	0.611	24.6	99.99	0.15
2021/5/18 9:10	0.611	24.6	99.94	0.15
2021/5/18 9:20	0.615	24.6	99.93	0.14
2021/5/18 9:30	0.618	24.6	99.91	0.13
2021/5/18 9:40	0.614	24.7	99.94	0.15
2021/5/18 9:50	0.617	24.7	99.94	0.13
2021/5/18 10:00	0.617	24.7	99.93	0.14
2021/5/18 10:10	0.617	24.8	99.93	0.14
2021/5/18 10:20	0.617	24.8	99.91	0.14
2021/5/18 10:30	0.620	24.8	99.91	0.13
2021/5/18 10:40	0.620	24.8	99.88	0.15
2021/5/18 10:50	0.623	24.8	99.84	0.15
2021/5/18 11:00	0.623	24.8	99.80	0.14
2021/5/18 11:10	0.623	24.8	99.78	0.16
2021/5/18 11:20	0.627	24.8	99.77	0.16
2021/5/18 11:30	0.627	24.8	99.77	0.15
2021/5/18 11:40	0.627	24.8	99.75	0.15
2021/5/18 11:50	0.630	24.8	99.77	0.15
2021/5/18 12:00	0.630	24.8	99.74	0.16
2021/5/18 12:10	0.630	24.8	99.71	0.15
2021/5/18 12:20	0.630	24.8	99.68	0.15
2021/5/18 12:30	0.633	24.8	99.66	0.16
2021/5/18 12:40	0.633	24.8	99.65	0.16

2021/5/18 12:50	0.633	24.8	99.64	0.16
2021/5/18 13:00	0.636	24.8	99.64	0.17
2021/5/18 13:10	0.636	24.8	99.65	0.16
2021/5/18 13:20	0.632	24.9	99.65	0.17
2021/5/18 13:30	0.636	24.9	99.65	0.17
2021/5/18 13:40	0.636	24.9	99.65	0.17
2021/5/18 13:50	0.639	24.9	99.68	0.16
2021/5/18 14:00	0.639	24.9	99.69	0.16
2021/5/18 14:10	0.639	24.9	99.68	0.16
2021/5/18 14:20	0.642	24.9	99.69	0.16
2021/5/18 14:30	0.638	25.0	99.69	0.16
2021/5/18 14:40	0.641	25.0	99.71	0.16
2021/5/18 14:50	0.641	25.0	99.74	0.15
2021/5/18 15:00	0.641	25.0	99.77	0.16
2021/5/18 15:10	0.641	25.0	99.80	0.16
2021/5/18 15:20	0.641	25.0	99.83	0.16
2021/5/18 15:30	0.641	25.0	99.84	0.15
2021/5/18 15:40	0.644	25.0	99.84	0.15
2021/5/18 15:50	0.644	25.0	99.85	0.15
2021/5/18 16:00	0.644	25.0	99.85	0.15
2021/5/18 16:10	0.644	25.0	99.85	0.15
2021/5/18 16:20	0.648	25.0	99.85	0.16
2021/5/18 16:30	0.644	25.1	99.87	0.15
2021/5/18 16:40	0.647	25.1	99.88	0.15
2021/5/18 16:50	0.647	25.1	99.88	0.15
2021/5/18 17:00	0.647	25.1	99.88	0.15
2021/5/18 17:10	0.650	25.1	99.90	0.13
2021/5/18 17:20	0.650	25.1	99.93	0.14
2021/5/18 17:30	0.653	25.1	99.94	0.15
2021/5/18 17:40	0.653	25.1	99.94	0.14
2021/5/18 17:50	0.653	25.1	99.96	0.14
2021/5/18 18:00	0.656	25.1	99.97	0.14
2021/5/18 18:10	0.656	25.1	99.99	0.14
2021/5/18 18:20	0.659	25.1	99.99	0.12
2021/5/18 18:30	0.659	25.1	99.99	0.06
2021/5/18 18:40	0.658	24.9	99.99	0.05
2021/5/18 18:50	0.663	24.6	100.00	0.04
2021/5/18 19:00	0.664	24.5	100.00	0.04
2021/5/18 19:10	0.673	24.2	100.00	0.04
2021/5/18 19:20	0.673	24.1	100.02	0.04
2021/5/18 19:30	0.674	24.0	100.03	0.03
2021/5/18 19:40	0.675	23.9	100.05	0.03
2021/5/18 19:50	0.679	23.8	100.07	0.03
2021/5/18 20:00	0.679	23.7	100.07	0.04
2021/5/18 20:10	0.679	23.7	100.06	0.03
2021/5/18 20:20	0.680	23.6	100.07	0.03
2021/5/18 20:30	0.680	23.6	100.07	0.03
2021/5/18 20:40	0.684	23.5	100.09	0.03
2021/5/18 20:50	0.681	23.5	100.12	0.03
2021/5/18 21:00	0.681	23.5	100.13	0.02
2021/5/18 21:10	0.685	23.4	100.16	0.02
2021/5/18 21:20	0.681	23.4	100.18	0.02
2021/5/18 21:30	0.681	23.4	100.18	0.02
2021/5/18 21:40	0.681	23.4	100.19	0.02
2021/5/18 21:50	0.681	23.4	100.19	0.02
2021/5/18 22:00	0.686	23.3	100.22	0.03

2021/5/18 22:10	0.682	23.3	100.24	0.02
2021/5/18 22:20	0.682	23.3	100.27	0.02
2021/5/18 22:30	0.686	23.2	100.27	0.03
2021/5/18 22:40	0.686	23.2	100.27	0.03
2021/5/18 22:50	0.683	23.2	100.28	0.03
2021/5/18 23:00	0.683	23.2	100.31	0.03
2021/5/18 23:10	0.683	23.2	100.32	0.03
2021/5/18 23:20	0.679	23.2	100.32	0.03
2021/5/18 23:30	0.683	23.1	100.32	0.03
2021/5/18 23:40	0.683	23.1	100.34	0.03
2021/5/18 23:50	0.680	23.1	100.35	0.04
2021/5/19 0:00	0.680	23.1	100.38	0.04
2021/5/19 0:10	0.680	23.1	100.40	0.04
2021/5/19 0:20	0.684	23.0	100.41	0.04
2021/5/19 0:30	0.680	23.0	100.41	0.04
2021/5/19 0:40	0.680	23.0	100.41	0.04
2021/5/19 0:50	0.680	23.0	100.41	0.04
2021/5/19 1:00	0.677	23.0	100.41	0.04
2021/5/19 1:10	0.681	22.9	100.41	0.04
2021/5/19 1:20	0.681	22.9	100.41	0.04
2021/5/19 1:30	0.677	22.9	100.43	0.05
2021/5/19 1:40	0.677	22.9	100.43	0.05
2021/5/19 1:50	0.677	22.9	100.43	0.05
2021/5/19 2:00	0.674	22.9	100.44	0.05
2021/5/19 2:10	0.678	22.8	100.44	0.05
2021/5/19 2:20	0.678	22.8	100.46	0.05
2021/5/19 2:30	0.674	22.8	100.46	0.05
2021/5/19 2:40	0.674	22.8	100.46	0.04
2021/5/19 2:50	0.674	22.8	100.46	0.05
2021/5/19 3:00	0.671	22.8	100.46	0.04
2021/5/19 3:10	0.675	22.7	100.44	0.04
2021/5/19 3:20	0.675	22.7	100.44	0.04
2021/5/19 3:30	0.675	22.7	100.44	0.05
2021/5/19 3:40	0.671	22.7	100.46	0.04
2021/5/19 3:50	0.671	22.7	100.44	0.04
2021/5/19 4:00	0.675	22.6	100.43	0.04
2021/5/19 4:10	0.671	22.6	100.44	0.04
2021/5/19 4:20	0.671	22.6	100.44	0.04
2021/5/19 4:30	0.671	22.6	100.44	0.03
2021/5/19 4:40	0.671	22.6	100.44	0.03
2021/5/19 4:50	0.672	22.5	100.46	0.03
2021/5/19 5:00	0.672	22.5	100.47	0.03
2021/5/19 5:10	0.668	22.5	100.47	0.03
2021/5/19 5:20	0.668	22.5	100.49	0.03
2021/5/19 5:30	0.668	22.5	100.50	0.04
2021/5/19 5:40	0.668	22.5	100.50	0.03
2021/5/19 5:50	0.672	22.4	100.51	0.03
2021/5/19 6:00	0.669	22.4	100.53	0.03
2021/5/19 6:10	0.669	22.4	100.53	0.03
2021/5/19 6:20	0.669	22.4	100.54	0.05
2021/5/19 6:30	0.668	22.5	100.54	0.11
2021/5/19 6:40	0.663	22.8	100.54	0.12
2021/5/19 6:50	0.659	23.1	100.56	0.12
2021/5/19 7:00	0.658	23.3	100.56	0.12
2021/5/19 7:10	0.653	23.5	100.56	0.12
2021/5/19 7:20	0.649	23.7	100.56	0.13

2021/5/19 7:30	0.648	23.8	100.54	0.13
2021/5/19 7:40	0.652	23.8	100.54	0.13
2021/5/19 7:50	0.648	23.9	100.53	0.13
2021/5/19 8:00	0.647	24.0	100.53	0.13
2021/5/19 8:10	0.647	24.0	100.53	0.15
2021/5/19 8:20	0.651	24.0	100.53	0.15
2021/5/19 8:30	0.647	24.1	100.53	0.15
2021/5/19 8:40	0.647	24.1	100.54	0.16
2021/5/19 8:50	0.650	24.1	100.54	0.17
2021/5/19 9:00	0.650	24.1	100.53	0.17
2021/5/19 9:10	0.646	24.2	100.51	0.17
2021/5/19 9:20	0.649	24.2	100.49	0.18
2021/5/19 9:30	0.649	24.2	100.49	0.18
2021/5/19 9:40	0.646	24.3	100.47	0.18
2021/5/19 9:50	0.649	24.3	100.47	0.18
2021/5/19 10:00	0.649	24.3	100.47	0.18
2021/5/19 10:10	0.648	24.4	100.46	0.18
2021/5/19 10:20	0.648	24.4	100.41	0.19
2021/5/19 10:30	0.648	24.5	100.41	0.19
2021/5/19 10:40	0.648	24.5	100.40	0.19
2021/5/19 10:50	0.648	24.5	100.38	0.18
2021/5/19 11:00	0.644	24.6	100.38	0.18
2021/5/19 11:10	0.644	24.6	100.37	0.17
2021/5/19 11:20	0.640	24.7	100.34	0.17
2021/5/19 11:30	0.640	24.7	100.34	0.17
2021/5/19 11:40	0.640	24.7	100.31	0.17
2021/5/19 11:50	0.636	24.8	100.31	0.17
2021/5/19 12:00	0.636	24.8	100.28	0.17
2021/5/19 12:10	0.632	24.9	100.25	0.16
2021/5/19 12:20	0.632	24.9	100.22	0.17
2021/5/19 12:30	0.632	24.9	100.21	0.16
2021/5/19 12:40	0.632	24.9	100.19	0.16
2021/5/19 12:50	0.629	25.0	100.18	0.16
2021/5/19 13:00	0.629	25.0	100.15	0.16
2021/5/19 13:10	0.629	25.0	100.12	0.17
2021/5/19 13:20	0.625	25.1	100.09	0.17
2021/5/19 13:30	0.625	25.1	100.06	0.16
2021/5/19 13:40	0.625	25.1	100.03	0.16
2021/5/19 13:50	0.625	25.1	100.03	0.15
2021/5/19 14:00	0.625	25.1	100.02	0.16
2021/5/19 14:10	0.624	25.2	100.00	0.16
2021/5/19 14:20	0.624	25.2	99.97	0.16
2021/5/19 14:30	0.621	25.2	99.99	0.15
2021/5/19 14:40	0.621	25.2	99.96	0.14
2021/5/19 14:50	0.621	25.2	99.96	0.14
2021/5/19 15:00	0.621	25.2	99.94	0.14
2021/5/19 15:10	0.621	25.2	99.93	0.16
2021/5/19 15:20	0.618	25.2	99.94	0.14
2021/5/19 15:30	0.618	25.2	99.96	0.15
2021/5/19 15:40	0.618	25.2	99.96	0.14
2021/5/19 15:50	0.615	25.2	99.96	0.14
2021/5/19 16:00	0.615	25.2	99.93	0.14
2021/5/19 16:10	0.615	25.2	99.93	0.14
2021/5/19 16:20	0.612	25.2	99.93	0.13
2021/5/19 16:30	0.611	25.3	99.93	0.14
2021/5/19 16:40	0.611	25.3	99.93	0.14

2021/5/19 16:50	0.611	25.3	99.93	0.14
2021/5/19 17:00	0.611	25.3	99.91	0.15
2021/5/19 17:10	0.611	25.3	99.90	0.15
2021/5/19 17:20	0.608	25.3	99.90	0.14
2021/5/19 17:30	0.611	25.3	99.90	0.14
2021/5/19 17:40	0.611	25.3	99.88	0.13
2021/5/19 17:50	0.611	25.3	99.88	0.13
2021/5/19 18:00	0.611	25.3	99.90	0.14
2021/5/19 18:10	0.611	25.3	99.90	0.13
2021/5/19 18:20	0.611	25.3	99.88	0.11
2021/5/19 18:30	0.611	25.3	99.88	0.07
2021/5/19 18:40	0.612	25.2	99.90	0.04
2021/5/19 18:50	0.612	25.1	99.88	0.04
2021/5/19 19:00	0.616	24.9	99.88	0.03
2021/5/19 19:10	0.617	24.8	99.87	0.03
2021/5/19 19:20	0.617	24.7	99.87	0.03
2021/5/19 19:30	0.618	24.6	99.84	0.03
2021/5/19 19:40	0.618	24.5	99.81	0.03
2021/5/19 19:50	0.615	24.5	99.81	0.03
2021/5/19 20:00	0.615	24.4	99.81	0.03
2021/5/19 20:10	0.616	24.3	99.81	0.03
2021/5/19 20:20	0.616	24.3	99.81	0.04
2021/5/19 20:30	0.616	24.2	99.81	0.04
2021/5/19 20:40	0.616	24.2	99.80	0.04
2021/5/19 20:50	0.617	24.1	99.81	0.04
2021/5/19 21:00	0.617	24.0	99.80	0.04
2021/5/19 21:10	0.617	24.0	99.78	0.04
2021/5/19 21:20	0.617	24.0	99.78	0.04
2021/5/19 21:30	0.617	23.9	99.83	0.03
2021/5/19 21:40	0.617	23.9	99.81	0.04
2021/5/19 21:50	0.617	23.9	99.78	0.03
2021/5/19 22:00	0.617	23.9	99.78	0.03
2021/5/19 22:10	0.621	23.8	99.93	0.04
2021/5/19 22:20	0.621	23.8	99.99	0.03
2021/5/19 22:30	0.621	23.8	100.09	0.04
2021/5/19 22:40	0.618	23.8	100.13	0.03
2021/5/19 22:50	0.618	23.8	100.15	0.03
2021/5/19 23:00	0.621	23.7	100.18	0.03
2021/5/19 23:10	0.618	23.7	100.16	0.03
2021/5/19 23:20	0.622	23.6	100.15	0.02
2021/5/19 23:30	0.622	23.6	100.13	0.03
2021/5/19 23:40	0.622	23.6	100.12	0.03
2021/5/19 23:50	0.626	23.5	100.12	0.03
2021/5/20 0:00	0.626	23.5	100.12	0.03
2021/5/20 0:10	0.626	23.5	100.09	0.03
2021/5/20 0:20	0.626	23.5	100.06	0.03
2021/5/20 0:30	0.629	23.5	100.03	0.03
2021/5/20 0:40	0.633	23.4	99.99	0.03
2021/5/20 0:50	0.633	23.4	99.99	0.03
2021/5/20 1:00	0.633	23.4	99.99	0.03
2021/5/20 1:10	0.633	23.4	99.94	0.03
2021/5/20 1:20	0.633	23.4	99.94	0.03
2021/5/20 1:30	0.633	23.4	99.96	0.03
2021/5/20 1:40	0.633	23.4	99.93	0.02
2021/5/20 1:50	0.633	23.4	99.93	0.03
2021/5/20 2:00	0.637	23.3	99.88	0.03

2021/5/20 2:10	0.637	23.3	99.87	0.03
2021/5/20 2:20	0.637	23.3	99.85	0.03
2021/5/20 2:30	0.637	23.3	99.85	0.03
2021/5/20 2:40	0.637	23.3	99.85	0.03
2021/5/20 2:50	0.640	23.2	99.87	0.03
2021/5/20 3:00	0.640	23.2	99.87	0.03
2021/5/20 3:10	0.640	23.2	99.88	0.03
2021/5/20 3:20	0.640	23.2	99.88	0.03
2021/5/20 3:30	0.640	23.2	99.87	0.03
2021/5/20 3:40	0.640	23.2	99.88	0.03
2021/5/20 3:50	0.640	23.2	99.88	0.03
2021/5/20 4:00	0.640	23.2	99.88	0.04
2021/5/20 4:10	0.640	23.2	99.90	0.03
2021/5/20 4:20	0.640	23.2	99.91	0.03
2021/5/20 4:30	0.648	23.1	99.93	0.04
2021/5/20 4:40	0.648	23.1	99.94	0.04
2021/5/20 4:50	0.648	23.1	99.96	0.04
2021/5/20 5:00	0.648	23.1	99.96	0.04
2021/5/20 5:10	0.648	23.1	99.96	0.04
2021/5/20 5:20	0.648	23.1	99.96	0.04
2021/5/20 5:30	0.648	23.1	99.97	0.04
2021/5/20 5:40	0.652	23.0	99.99	0.05
2021/5/20 5:50	0.652	23.0	99.99	0.05
2021/5/20 6:00	0.652	23.0	99.99	0.05
2021/5/20 6:10	0.652	23.0	99.99	0.04
2021/5/20 6:20	0.655	23.0	100.00	0.06
2021/5/20 6:30	0.651	23.2	100.03	0.10
2021/5/20 6:40	0.654	23.4	100.03	0.13
2021/5/20 6:50	0.649	23.7	100.05	0.15
2021/5/20 7:00	0.644	24.0	100.06	0.14
2021/5/20 7:10	0.643	24.2	100.06	0.14
2021/5/20 7:20	0.642	24.3	100.07	0.16
2021/5/20 7:30	0.642	24.4	100.09	0.16
2021/5/20 7:40	0.641	24.5	100.09	0.14
2021/5/20 7:50	0.644	24.5	100.10	0.14
2021/5/20 8:00	0.644	24.6	100.12	0.16
2021/5/20 8:10	0.644	24.6	100.15	0.16
2021/5/20 8:20	0.643	24.7	100.16	0.15
2021/5/20 8:30	0.643	24.7	100.18	0.16
2021/5/20 8:40	0.643	24.7	100.19	0.16
2021/5/20 8:50	0.639	24.8	100.22	0.17
2021/5/20 9:00	0.639	24.8	100.24	0.17
2021/5/20 9:10	0.639	24.8	100.25	0.16
2021/5/20 9:20	0.639	24.8	100.25	0.17
2021/5/20 9:30	0.639	24.8	100.27	0.17
2021/5/20 9:40	0.636	24.9	100.27	0.16
2021/5/20 9:50	0.636	24.9	100.29	0.16
2021/5/20 10:00	0.636	24.9	100.31	0.15
2021/5/20 10:10	0.636	24.9	100.32	0.15
2021/5/20 10:20	0.632	25.0	100.35	0.16
2021/5/20 10:30	0.635	25.0	100.35	0.16
2021/5/20 10:40	0.635	25.0	100.38	0.16
2021/5/20 10:50	0.635	25.0	100.40	0.16
2021/5/20 11:00	0.635	25.0	100.40	0.16
2021/5/20 11:10	0.631	25.1	100.41	0.15
2021/5/20 11:20	0.628	25.1	100.41	0.16

2021/5/20 11:30	0.628	25.1	100.40	0.17
2021/5/20 11:40	0.628	25.1	100.41	0.15
2021/5/20 11:50	0.628	25.1	100.41	0.16
2021/5/20 12:00	0.628	25.1	100.41	0.15
2021/5/20 12:10	0.628	25.1	100.43	0.16
2021/5/20 12:20	0.628	25.1	100.43	0.16
2021/5/20 12:30	0.628	25.1	100.43	0.15
2021/5/20 12:40	0.628	25.1	100.44	0.15
2021/5/20 12:50	0.627	25.2	100.46	0.15
2021/5/20 13:00	0.627	25.2	100.46	0.15
2021/5/20 13:10	0.627	25.2	100.46	0.14
2021/5/20 13:20	0.627	25.2	100.47	0.13
2021/5/20 13:30	0.624	25.3	100.49	0.14
2021/5/20 13:40	0.624	25.3	100.47	0.13
2021/5/20 13:50	0.624	25.3	100.47	0.14
2021/5/20 14:00	0.624	25.3	100.49	0.13
2021/5/20 14:10	0.624	25.3	100.49	0.12
2021/5/20 14:20	0.624	25.3	100.49	0.13
2021/5/20 14:30	0.624	25.3	100.49	0.14
2021/5/20 14:40	0.620	25.4	100.47	0.14
2021/5/20 14:50	0.623	25.4	100.46	0.12
2021/5/20 15:00	0.623	25.4	100.46	0.13
2021/5/20 15:10	0.623	25.4	100.44	0.13
2021/5/20 15:20	0.623	25.4	100.44	0.12
2021/5/20 15:30	0.620	25.4	100.46	0.13
2021/5/20 15:40	0.623	25.4	100.46	0.13
2021/5/20 15:50	0.620	25.4	100.47	0.12
2021/5/20 16:00	0.620	25.4	100.49	0.14
2021/5/20 16:10	0.619	25.5	100.50	0.12
2021/5/20 16:20	0.619	25.5	100.53	0.12
2021/5/20 16:30	0.619	25.5	100.51	0.13
2021/5/20 16:40	0.619	25.5	100.51	0.13
2021/5/20 16:50	0.622	25.5	100.54	0.12
2021/5/20 17:00	0.622	25.5	100.54	0.14
2021/5/20 17:10	0.622	25.5	100.56	0.13
2021/5/20 17:20	0.622	25.5	100.56	0.12
2021/5/20 17:30	0.622	25.5	100.54	0.13
2021/5/20 17:40	0.625	25.5	100.56	0.13
2021/5/20 17:50	0.625	25.5	100.56	0.12
2021/5/20 18:00	0.622	25.6	100.57	0.12
2021/5/20 18:10	0.622	25.6	100.57	0.11
2021/5/20 18:20	0.625	25.6	100.59	0.11
2021/5/20 18:30	0.629	25.5	100.60	0.08
2021/5/20 18:40	0.626	25.4	100.62	0.05
2021/5/20 18:50	0.624	25.2	100.63	0.05
2021/5/20 19:00	0.625	25.1	100.65	0.04
2021/5/20 19:10	0.626	24.9	100.65	0.04
2021/5/20 19:20	0.630	24.7	100.66	0.04
2021/5/20 19:30	0.631	24.5	100.65	0.04
2021/5/20 19:40	0.632	24.4	100.66	0.04
2021/5/20 19:50	0.636	24.3	100.66	0.04
2021/5/20 20:00	0.636	24.2	100.68	0.03
2021/5/20 20:10	0.633	24.2	100.69	0.04
2021/5/20 20:20	0.633	24.1	100.69	0.04
2021/5/20 20:30	0.633	24.1	100.70	0.03
2021/5/20 20:40	0.634	24.0	100.72	0.03

2021/5/20 20:50	0.634	24.0	100.73	0.04
2021/5/20 21:00	0.634	24.0	100.73	0.03
2021/5/20 21:10	0.634	23.9	100.73	0.04
2021/5/20 21:20	0.634	23.9	100.72	0.03
2021/5/20 21:30	0.634	23.9	100.72	0.04
2021/5/20 21:40	0.631	23.9	100.72	0.04
2021/5/20 21:50	0.631	23.9	100.73	0.04
2021/5/20 22:00	0.635	23.8	100.75	0.03
2021/5/20 22:10	0.635	23.8	100.78	0.04
2021/5/20 22:20	0.635	23.8	100.79	0.03
2021/5/20 22:30	0.635	23.8	100.78	0.03
2021/5/20 22:40	0.635	23.8	100.76	0.03
2021/5/20 22:50	0.635	23.7	100.78	0.04
2021/5/20 23:00	0.635	23.7	100.76	0.03
2021/5/20 23:10	0.635	23.7	100.76	0.03
2021/5/20 23:20	0.635	23.7	100.76	0.03
2021/5/20 23:30	0.635	23.7	100.75	0.02
2021/5/20 23:40	0.639	23.6	100.73	0.02
2021/5/20 23:50	0.635	23.6	100.70	0.02
2021/5/21 0:00	0.635	23.6	100.70	0.03
2021/5/21 0:10	0.635	23.6	100.70	0.03
2021/5/21 0:20	0.635	23.6	100.68	0.03
2021/5/21 0:30	0.639	23.5	100.68	0.03
2021/5/21 0:40	0.636	23.5	100.68	0.03
2021/5/21 0:50	0.636	23.5	100.66	0.02
2021/5/21 1:00	0.636	23.5	100.68	0.03
2021/5/21 1:10	0.636	23.5	100.68	0.03
2021/5/21 1:20	0.632	23.5	100.68	0.03
2021/5/21 1:30	0.632	23.5	100.66	0.03
2021/5/21 1:40	0.636	23.4	100.66	0.03
2021/5/21 1:50	0.633	23.4	100.68	0.03
2021/5/21 2:00	0.633	23.4	100.68	0.03
2021/5/21 2:10	0.633	23.4	100.68	0.03
2021/5/21 2:20	0.633	23.4	100.68	0.03
2021/5/21 2:30	0.633	23.4	100.68	0.03
2021/5/21 2:40	0.633	23.3	100.69	0.03
2021/5/21 2:50	0.633	23.3	100.70	0.03
2021/5/21 3:00	0.633	23.3	100.70	0.03
2021/5/21 3:10	0.630	23.3	100.69	0.03
2021/5/21 3:20	0.630	23.3	100.70	0.04
2021/5/21 3:30	0.626	23.3	100.69	0.03
2021/5/21 3:40	0.630	23.2	100.69	0.03
2021/5/21 3:50	0.630	23.2	100.69	0.03
2021/5/21 4:00	0.626	23.2	100.69	0.03
2021/5/21 4:10	0.626	23.2	100.69	0.03
2021/5/21 4:20	0.626	23.2	100.69	0.03
2021/5/21 4:30	0.623	23.2	100.69	0.04
2021/5/21 4:40	0.623	23.2	100.70	0.03
2021/5/21 4:50	0.627	23.1	100.73	0.03
2021/5/21 5:00	0.623	23.1	100.73	0.04
2021/5/21 5:10	0.623	23.1	100.75	0.04
2021/5/21 5:20	0.620	23.1	100.75	0.04
2021/5/21 5:30	0.623	23.0	100.76	0.04
2021/5/21 5:40	0.623	23.0	100.79	0.04
2021/5/21 5:50	0.620	23.0	100.81	0.04
2021/5/21 6:00	0.620	23.0	100.79	0.05

2021/5/21 6:10	0.616	23.0	100.78	0.05
2021/5/21 6:20	0.616	23.0	100.78	0.05
2021/5/21 6:30	0.616	23.1	100.78	0.10
2021/5/21 6:40	0.612	23.4	100.76	0.10
2021/5/21 6:50	0.608	23.7	100.78	0.12
2021/5/21 7:00	0.607	23.9	100.79	0.13
2021/5/21 7:10	0.603	24.1	100.79	0.13
2021/5/21 7:20	0.599	24.3	100.79	0.13
2021/5/21 7:30	0.599	24.4	100.81	0.14
2021/5/21 7:40	0.596	24.5	100.82	0.14
2021/5/21 7:50	0.596	24.5	100.82	0.14
2021/5/21 8:00	0.595	24.6	100.82	0.15
2021/5/21 8:10	0.595	24.6	100.84	0.14
2021/5/21 8:20	0.595	24.7	100.85	0.15
2021/5/21 8:30	0.595	24.7	100.84	0.15
2021/5/21 8:40	0.595	24.7	100.84	0.16
2021/5/21 8:50	0.595	24.8	100.84	0.16
2021/5/21 9:00	0.591	24.8	100.84	0.17
2021/5/21 9:10	0.591	24.8	100.84	0.18
2021/5/21 9:20	0.595	24.8	100.82	0.17
2021/5/21 9:30	0.595	24.8	100.82	0.18
2021/5/21 9:40	0.595	24.8	100.82	0.17
2021/5/21 9:50	0.594	24.9	100.82	0.17
2021/5/21 10:00	0.594	24.9	100.82	0.18
2021/5/21 10:10	0.594	24.9	100.84	0.16
2021/5/21 10:20	0.594	25.0	100.84	0.17
2021/5/21 10:30	0.597	25.0	100.84	0.16
2021/5/21 10:40	0.593	25.1	100.84	0.15
2021/5/21 10:50	0.593	25.1	100.84	0.16
2021/5/21 11:00	0.593	25.2	100.84	0.15
2021/5/21 11:10	0.593	25.2	100.84	0.14
2021/5/21 11:20	0.593	25.2	100.84	0.14
2021/5/21 11:30	0.590	25.3	100.85	0.13
2021/5/21 11:40	0.593	25.3	100.84	0.14
2021/5/21 11:50	0.593	25.3	100.84	0.15
2021/5/21 12:00	0.596	25.3	100.84	0.14
2021/5/21 12:10	0.599	25.3	100.82	0.14
2021/5/21 12:20	0.599	25.3	100.82	0.16
2021/5/21 12:30	0.602	25.3	100.82	0.14
2021/5/21 12:40	0.605	25.3	100.82	0.14
2021/5/21 12:50	0.601	25.4	100.82	0.15
2021/5/21 13:00	0.608	25.3	100.81	0.14
2021/5/21 13:10	0.608	25.4	100.81	0.15
2021/5/21 13:20	0.608	25.4	100.81	0.14
2021/5/21 13:30	0.611	25.4	100.79	0.13
2021/5/21 13:40	0.614	25.4	100.79	0.14
2021/5/21 13:50	0.614	25.4	100.79	0.14
2021/5/21 14:00	0.617	25.4	100.79	0.14
2021/5/21 14:10	0.620	25.4	100.81	0.13
2021/5/21 14:20	0.620	25.4	100.81	0.14
2021/5/21 14:30	0.620	25.4	100.81	0.13
2021/5/21 14:40	0.623	25.4	100.81	0.12
2021/5/21 14:50	0.619	25.5	100.81	0.12
2021/5/21 15:00	0.619	25.5	100.82	0.14
2021/5/21 15:10	0.622	25.5	100.82	0.12
2021/5/21 15:20	0.622	25.5	100.84	0.13

2021/5/21 15:30	0.622	25.5	100.82	0.11
2021/5/21 15:40	0.622	25.5	100.84	0.13
2021/5/21 15:50	0.625	25.5	100.84	0.13
2021/5/21 16:00	0.625	25.5	100.84	0.13
2021/5/21 16:10	0.625	25.5	100.84	0.13
2021/5/21 16:20	0.629	25.5	100.84	0.14
2021/5/21 16:30	0.625	25.5	100.84	0.13
2021/5/21 16:40	0.629	25.5	100.84	0.14
2021/5/21 16:50	0.625	25.5	100.84	0.14
2021/5/21 17:00	0.634	25.2	100.84	0.15
2021/5/21 17:10	0.641	25.1	100.84	0.13
2021/5/21 17:20	0.640	25.2	100.84	0.14
2021/5/21 17:30	0.636	25.3	100.85	0.12
2021/5/21 17:40	0.635	25.4	100.87	0.13
2021/5/21 17:50	0.632	25.5	100.87	0.12
2021/5/21 18:00	0.632	25.5	100.87	0.12
2021/5/21 18:10	0.631	25.6	100.87	0.13
2021/5/21 18:20	0.631	25.6	100.88	0.09
2021/5/21 18:30	0.631	25.6	100.88	0.07
2021/5/21 18:40	0.629	25.5	100.90	0.06
2021/5/21 18:50	0.626	25.4	100.90	0.05
2021/5/21 19:00	0.630	25.2	100.90	0.04
2021/5/21 19:10	0.628	25.1	100.91	0.04
2021/5/21 19:20	0.632	24.9	100.92	0.05
2021/5/21 19:30	0.633	24.7	100.92	0.04
2021/5/21 19:40	0.634	24.6	100.92	0.04
2021/5/21 19:50	0.635	24.5	100.92	0.04
2021/5/21 20:00	0.635	24.4	100.92	0.03
2021/5/21 20:10	0.632	24.4	100.92	0.04
2021/5/21 20:20	0.632	24.3	100.92	0.03
2021/5/21 20:30	0.633	24.2	100.94	0.03
2021/5/21 20:40	0.633	24.2	100.94	0.03
2021/5/21 20:50	0.630	24.2	100.94	0.04
2021/5/21 21:00	0.630	24.1	100.95	0.04
2021/5/21 21:10	0.630	24.1	100.97	0.03
2021/5/21 21:20	0.627	24.1	100.97	0.03
2021/5/21 21:30	0.627	24.1	100.98	0.03
2021/5/21 21:40	0.630	24.0	101.00	0.03
2021/5/21 21:50	0.627	24.0	101.01	0.03
2021/5/21 22:00	0.627	24.0	101.01	0.03
2021/5/21 22:10	0.631	23.9	101.01	0.04
2021/5/21 22:20	0.631	23.9	101.03	0.03
2021/5/21 22:30	0.631	23.9	101.04	0.03
2021/5/21 22:40	0.631	23.9	101.04	0.03
2021/5/21 22:50	0.631	23.8	101.06	0.03
2021/5/21 23:00	0.631	23.8	101.06	0.03
2021/5/21 23:10	0.631	23.8	101.06	0.03
2021/5/21 23:20	0.628	23.8	101.06	0.03
2021/5/21 23:30	0.632	23.7	101.04	0.03
2021/5/21 23:40	0.632	23.7	101.04	0.03
2021/5/21 23:50	0.628	23.7	101.04	0.03
2021/5/22 0:00	0.628	23.7	101.04	0.02
2021/5/22 0:10	0.629	23.6	101.03	0.02
2021/5/22 0:20	0.629	23.6	101.04	0.02
2021/5/22 0:30	0.625	23.6	101.04	0.03
2021/5/22 0:40	0.625	23.6	101.03	0.03

2021/5/22 0:50	0.626	23.5	101.03	0.03
2021/5/22 1:00	0.626	23.5	101.04	0.02
2021/5/22 1:10	0.622	23.5	101.04	0.03
2021/5/22 1:20	0.622	23.5	101.04	0.03
2021/5/22 1:30	0.622	23.4	101.04	0.03
2021/5/22 1:40	0.619	23.4	101.04	0.04
2021/5/22 1:50	0.619	23.4	101.06	0.03
2021/5/22 2:00	0.619	23.4	101.06	0.04
2021/5/22 2:10	0.619	23.3	101.04	0.03
2021/5/22 2:20	0.619	23.3	101.03	0.04
2021/5/22 2:30	0.616	23.3	101.03	0.03
2021/5/22 2:40	0.619	23.2	101.03	0.04
2021/5/22 2:50	0.616	23.2	101.01	0.03
2021/5/22 3:00	0.612	23.2	101.01	0.04
2021/5/22 3:10	0.612	23.2	101.01	0.04
2021/5/22 3:20	0.616	23.1	101.01	0.04
2021/5/22 3:30	0.612	23.1	101.01	0.04
2021/5/22 3:40	0.609	23.1	101.01	0.04
2021/5/22 3:50	0.609	23.1	101.00	0.05
2021/5/22 4:00	0.609	23.0	101.01	0.04
2021/5/22 4:10	0.609	23.0	101.01	0.04
2021/5/22 4:20	0.606	23.0	101.01	0.04
2021/5/22 4:30	0.602	23.0	101.03	0.04
2021/5/22 4:40	0.606	22.9	101.04	0.04
2021/5/22 4:50	0.602	22.9	101.04	0.04
2021/5/22 5:00	0.602	22.9	101.06	0.03
2021/5/22 5:10	0.598	22.9	101.06	0.03
2021/5/22 5:20	0.602	22.8	101.04	0.03
2021/5/22 5:30	0.598	22.8	101.04	0.03
2021/5/22 5:40	0.598	22.8	101.04	0.03
2021/5/22 5:50	0.595	22.8	101.04	0.03
2021/5/22 6:00	0.598	22.7	101.04	0.03
2021/5/22 6:10	0.595	22.7	101.04	0.03
2021/5/22 6:20	0.595	22.7	101.04	0.04
2021/5/22 6:30	0.591	22.8	101.04	0.11
2021/5/22 6:40	0.588	23.1	101.04	0.11
2021/5/22 6:50	0.584	23.4	101.04	0.10
2021/5/22 7:00	0.584	23.6	101.04	0.11
2021/5/22 7:10	0.580	23.8	101.06	0.11
2021/5/22 7:20	0.580	23.9	101.04	0.10
2021/5/22 7:30	0.577	24.0	101.04	0.11
2021/5/22 7:40	0.577	24.1	101.04	0.11
2021/5/22 7:50	0.573	24.2	101.03	0.12
2021/5/22 8:00	0.577	24.2	101.03	0.11
2021/5/22 8:10	0.573	24.3	101.04	0.12
2021/5/22 8:20	0.573	24.3	101.04	0.13
2021/5/22 8:30	0.573	24.3	101.06	0.14
2021/5/22 8:40	0.573	24.3	101.06	0.16
2021/5/22 8:50	0.573	24.3	101.04	0.17
2021/5/22 9:00	0.573	24.4	101.04	0.17
2021/5/22 9:10	0.573	24.4	101.03	0.18
2021/5/22 9:20	0.576	24.4	101.03	0.18
2021/5/22 9:30	0.579	24.4	101.03	0.19
2021/5/22 9:40	0.579	24.5	101.04	0.18
2021/5/22 9:50	0.583	24.5	101.04	0.18
2021/5/22 10:00	0.583	24.5	101.04	0.20

2021/5/22 10:10	0.582	24.6	101.03	0.18
2021/5/22 10:20	0.582	24.7	101.03	0.17
2021/5/22 10:30	0.582	24.8	101.01	0.16
2021/5/22 10:40	0.585	24.8	101.03	0.16
2021/5/22 10:50	0.588	24.9	101.03	0.12
2021/5/22 11:00	0.581	25.1	101.04	0.13
2021/5/22 11:10	0.581	25.2	101.04	0.13
2021/5/22 11:20	0.581	25.2	101.04	0.14
2021/5/22 11:30	0.577	25.3	101.04	0.12
2021/5/22 11:40	0.586	25.3	101.04	0.10
2021/5/22 11:50	0.583	25.4	101.04	0.06
2021/5/22 12:00	0.579	25.5	101.04	0.07
2021/5/22 12:10	0.579	25.5	101.04	0.08
2021/5/22 12:20	0.576	25.6	101.04	0.06
2021/5/22 12:30	0.576	25.6	101.03	0.06
2021/5/22 12:40	0.576	25.6	101.03	0.07
2021/5/22 12:50	0.579	25.6	101.01	0.06
2021/5/22 13:00	0.576	25.7	101.01	0.08
2021/5/22 13:10	0.576	25.7	101.03	0.06
2021/5/22 13:20	0.576	25.7	101.04	0.09
2021/5/22 13:30	0.576	25.7	101.06	0.10
2021/5/22 13:40	0.569	25.8	101.06	0.08
2021/5/22 13:50	0.569	25.8	101.04	0.09
2021/5/22 14:00	0.569	25.8	101.06	0.07
2021/5/22 14:10	0.566	25.9	101.06	0.09
2021/5/22 14:20	0.566	25.9	101.04	0.09
2021/5/22 14:30	0.566	25.9	101.04	0.10
2021/5/22 14:40	0.569	25.9	101.04	0.06
2021/5/22 14:50	0.569	25.8	101.04	0.06
2021/5/22 15:00	0.570	25.7	101.04	0.05
2021/5/22 15:10	0.573	25.6	101.04	0.06
2021/5/22 15:20	0.573	25.5	101.03	0.06
2021/5/22 15:30	0.573	25.5	101.03	0.06
2021/5/22 15:40	0.574	25.4	101.01	0.05
2021/5/22 15:50	0.574	25.4	101.01	0.05
2021/5/22 16:00	0.577	25.3	101.01	0.05
2021/5/22 16:10	0.577	25.3	101.01	0.06
2021/5/22 16:20	0.577	25.3	101.03	0.05
2021/5/22 16:30	0.577	25.2	101.03	0.04
2021/5/22 16:40	0.581	25.1	101.04	0.06
2021/5/22 16:50	0.581	25.0	101.06	0.04
2021/5/22 17:00	0.585	24.9	101.06	0.04
2021/5/22 17:10	0.585	24.9	101.04	0.04
2021/5/22 17:20	0.588	24.8	101.04	0.04
2021/5/22 17:30	0.588	24.8	101.06	0.04
2021/5/22 17:40	0.592	24.7	101.06	0.05
2021/5/22 17:50	0.592	24.7	101.07	0.05
2021/5/22 18:00	0.595	24.6	101.07	0.07
2021/5/22 18:10	0.595	24.6	101.07	0.08
2021/5/22 18:20	0.602	24.5	101.09	0.07
2021/5/22 18:30	0.605	24.5	101.10	0.06
2021/5/22 18:40	0.615	24.4	101.13	0.06
2021/5/22 18:50	0.619	24.4	101.16	0.07
2021/5/22 19:00	0.622	24.4	101.19	0.07
2021/5/22 19:10	0.632	24.3	101.19	0.08
2021/5/22 19:20	0.632	24.3	101.19	0.08

2021/5/22 19:30	0.636	24.2	101.20	0.08
2021/5/22 19:40	0.633	24.2	101.20	0.07
2021/5/22 19:50	0.633	24.1	101.22	0.07
2021/5/22 20:00	0.633	24.1	101.22	0.08
2021/5/22 20:10	0.634	24.0	101.22	0.07
2021/5/22 20:20	0.634	24.0	101.23	0.07
2021/5/22 20:30	0.634	23.9	101.25	0.06
2021/5/22 20:40	0.634	23.9	101.26	0.07
2021/5/22 20:50	0.634	23.9	101.26	0.07
2021/5/22 21:00	0.638	23.8	101.26	0.07
2021/5/22 21:10	0.635	23.8	101.29	0.06
2021/5/22 21:20	0.635	23.8	101.29	0.06
2021/5/22 21:30	0.635	23.8	101.29	0.07
2021/5/22 21:40	0.635	23.7	101.31	0.07
2021/5/22 21:50	0.635	23.7	101.31	0.06
2021/5/22 22:00	0.635	23.7	101.31	0.07
2021/5/22 22:10	0.635	23.7	101.34	0.06
2021/5/22 22:20	0.635	23.6	101.34	0.06
2021/5/22 22:30	0.635	23.6	101.34	0.06
2021/5/22 22:40	0.635	23.6	101.32	0.05
2021/5/22 22:50	0.635	23.6	101.32	0.05
2021/5/22 23:00	0.635	23.6	101.32	0.05
2021/5/22 23:10	0.639	23.5	101.31	0.05
2021/5/22 23:20	0.639	23.5	101.32	0.05
2021/5/22 23:30	0.639	23.5	101.32	0.05
2021/5/22 23:40	0.639	23.5	101.32	0.05
2021/5/22 23:50	0.639	23.5	101.32	0.05
2021/5/23 0:00	0.639	23.5	101.31	0.05
2021/5/23 0:10	0.640	23.4	101.31	0.06
2021/5/23 0:20	0.640	23.4	101.31	0.05
2021/5/23 0:30	0.640	23.4	101.31	0.05
2021/5/23 0:40	0.636	23.4	101.31	0.05
2021/5/23 0:50	0.636	23.4	101.31	0.05
2021/5/23 1:00	0.640	23.3	101.31	0.05
2021/5/23 1:10	0.637	23.3	101.31	0.05
2021/5/23 1:20	0.637	23.3	101.31	0.05
2021/5/23 1:30	0.637	23.2	101.29	0.05
2021/5/23 1:40	0.637	23.2	101.29	0.04
2021/5/23 1:50	0.637	23.2	101.28	0.05
2021/5/23 2:00	0.633	23.2	101.28	0.05
2021/5/23 2:10	0.633	23.2	101.28	0.05
2021/5/23 2:20	0.630	23.2	101.28	0.04
2021/5/23 2:30	0.630	23.2	101.29	0.05
2021/5/23 2:40	0.630	23.1	101.29	0.05
2021/5/23 2:50	0.630	23.1	101.29	0.05
2021/5/23 3:00	0.627	23.1	101.29	0.05
2021/5/23 3:10	0.627	23.0	101.31	0.04
2021/5/23 3:20	0.627	23.0	101.31	0.05
2021/5/23 3:30	0.623	23.0	101.32	0.05
2021/5/23 3:40	0.624	22.9	101.32	0.05
2021/5/23 3:50	0.624	22.9	101.32	0.05
2021/5/23 4:00	0.620	22.9	101.32	0.05
2021/5/23 4:10	0.620	22.9	101.34	0.05
2021/5/23 4:20	0.620	22.8	101.32	0.05
2021/5/23 4:30	0.616	22.8	101.34	0.05
2021/5/23 4:40	0.616	22.8	101.34	0.05

2021/5/23 4:50	0.617	22.7	101.32	0.05
2021/5/23 5:00	0.617	22.7	101.32	0.05
2021/5/23 5:10	0.613	22.7	101.35	0.05
2021/5/23 5:20	0.613	22.7	101.35	0.05
2021/5/23 5:30	0.613	22.6	101.35	0.04
2021/5/23 5:40	0.609	22.6	101.36	0.05
2021/5/23 5:50	0.609	22.6	101.38	0.04
2021/5/23 6:00	0.606	22.6	101.38	0.05
2021/5/23 6:10	0.609	22.5	101.39	0.04
2021/5/23 6:20	0.609	22.5	101.39	0.04
2021/5/23 6:30	0.606	22.5	101.39	0.05
2021/5/23 6:40	0.606	22.5	101.41	0.05
2021/5/23 6:50	0.602	22.5	101.41	0.05
2021/5/23 7:00	0.602	22.5	101.41	0.04
2021/5/23 7:10	0.602	22.5	101.39	0.04
2021/5/23 7:20	0.598	22.5	101.41	0.05
2021/5/23 7:30	0.598	22.5	101.41	0.04
2021/5/23 7:40	0.598	22.5	101.41	0.05
2021/5/23 7:50	0.598	22.5	101.39	0.04
2021/5/23 8:00	0.591	22.6	101.39	0.04
2021/5/23 8:10	0.591	22.6	101.39	0.04
2021/5/23 8:20	0.591	22.6	101.41	0.04
2021/5/23 8:30	0.591	22.6	101.41	0.04
2021/5/23 8:40	0.588	22.7	101.41	0.04
2021/5/23 8:50	0.591	22.7	101.41	0.05
2021/5/23 9:00	0.588	22.8	101.41	0.03
2021/5/23 9:10	0.588	22.8	101.41	0.03
2021/5/23 9:20	0.584	22.9	101.41	0.03
2021/5/23 9:30	0.584	22.9	101.41	0.03
2021/5/23 9:40	0.581	23.0	101.39	0.02
2021/5/23 9:50	0.581	23.0	101.39	0.03
2021/5/23 10:00	0.581	23.1	101.39	0.03
2021/5/23 10:10	0.581	23.1	101.39	0.02
2021/5/23 10:20	0.581	23.2	101.38	0.02
2021/5/23 10:30	0.581	23.2	101.38	0.03
2021/5/23 10:40	0.584	23.2	101.38	0.03
2021/5/23 10:50	0.588	23.2	101.38	0.03
2021/5/23 11:00	0.588	23.3	101.38	0.03
2021/5/23 11:10	0.591	23.3	101.38	0.02
2021/5/23 11:20	0.595	23.3	101.38	0.03
2021/5/23 11:30	0.595	23.3	101.38	0.02
2021/5/23 11:40	0.595	23.4	101.36	0.02
2021/5/23 11:50	0.598	23.4	101.36	0.02
2021/5/23 12:00	0.601	23.4	101.36	0.02
2021/5/23 12:10	0.605	23.4	101.36	0.03
2021/5/23 12:20	0.605	23.5	101.36	0.03
2021/5/23 12:30	0.608	23.5	101.35	0.02
2021/5/23 12:40	0.612	23.5	101.35	0.03
2021/5/23 12:50	0.612	23.5	101.34	0.03
2021/5/23 13:00	0.615	23.5	101.34	0.02
2021/5/23 13:10	0.615	23.6	101.32	0.03
2021/5/23 13:20	0.615	23.6	101.32	0.03
2021/5/23 13:30	0.618	23.6	101.31	0.03
2021/5/23 13:40	0.618	23.6	101.31	0.03
2021/5/23 13:50	0.622	23.6	101.31	0.03
2021/5/23 14:00	0.622	23.6	101.31	0.03

2021/5/23 14:10	0.625	23.6	101.32	0.03
2021/5/23 14:20	0.621	23.7	101.32	0.04
2021/5/23 14:30	0.625	23.7	101.31	0.04
2021/5/23 14:40	0.625	23.7	101.31	0.03
2021/5/23 14:50	0.625	23.7	101.29	0.04
2021/5/23 15:00	0.628	23.7	101.28	0.04
2021/5/23 15:10	0.628	23.7	101.26	0.03
2021/5/23 15:20	0.632	23.7	101.26	0.04
2021/5/23 15:30	0.632	23.7	101.25	0.03
2021/5/23 15:40	0.632	23.7	101.25	0.03
2021/5/23 15:50	0.635	23.7	101.25	0.04
2021/5/23 16:00	0.635	23.7	101.23	0.04
2021/5/23 16:10	0.635	23.7	101.20	0.04
2021/5/23 16:20	0.635	23.7	101.20	0.04
2021/5/23 16:30	0.635	23.7	101.22	0.03
2021/5/23 16:40	0.635	23.7	101.22	0.04
2021/5/23 16:50	0.635	23.7	101.22	0.04
2021/5/23 17:00	0.639	23.7	101.20	0.04
2021/5/23 17:10	0.635	23.7	101.22	0.03
2021/5/23 17:20	0.635	23.7	101.23	0.04
2021/5/23 17:30	0.639	23.7	101.25	0.04
2021/5/23 17:40	0.639	23.7	101.26	0.04
2021/5/23 17:50	0.639	23.7	101.26	0.03
2021/5/23 18:00	0.639	23.7	101.28	0.03
2021/5/23 18:10	0.639	23.7	101.29	0.03
2021/5/23 18:20	0.639	23.7	101.31	0.04
2021/5/23 18:30	0.639	23.7	101.32	0.03
2021/5/23 18:40	0.639	23.7	101.34	0.04
2021/5/23 18:50	0.642	23.7	101.35	0.03
2021/5/23 19:00	0.642	23.7	101.38	0.04
2021/5/23 19:10	0.642	23.7	101.38	0.04
2021/5/23 19:20	0.642	23.7	101.38	0.04
2021/5/23 19:30	0.642	23.7	101.38	0.04
2021/5/23 19:40	0.642	23.7	101.39	0.04
2021/5/23 19:50	0.646	23.6	101.38	0.05
2021/5/23 20:00	0.646	23.6	101.39	0.05
2021/5/23 20:10	0.646	23.6	101.41	0.04
2021/5/23 20:20	0.650	23.5	101.42	0.04
2021/5/23 20:30	0.650	23.5	101.45	0.05
2021/5/23 20:40	0.650	23.5	101.44	0.04
2021/5/23 20:50	0.654	23.4	101.47	0.04
2021/5/23 21:00	0.657	23.4	101.47	0.04
2021/5/23 21:10	0.657	23.4	101.47	0.05
2021/5/23 21:20	0.657	23.4	101.47	0.04
2021/5/23 21:30	0.657	23.4	101.47	0.04
2021/5/23 21:40	0.657	23.4	101.50	0.04
2021/5/23 21:50	0.657	23.4	101.47	0.04
2021/5/23 22:00	0.661	23.3	101.47	0.04
2021/5/23 22:10	0.661	23.3	101.45	0.04
2021/5/23 22:20	0.661	23.3	101.45	0.04
2021/5/23 22:30	0.661	23.3	101.44	0.04
2021/5/23 22:40	0.661	23.3	101.44	0.04
2021/5/23 22:50	0.661	23.3	101.42	0.04
2021/5/23 23:00	0.658	23.3	101.42	0.04
2021/5/23 23:10	0.658	23.3	101.42	0.04
2021/5/23 23:20	0.658	23.3	101.42	0.04

2021/5/23 23:30	0.658	23.3	101.42	0.04
2021/5/23 23:40	0.658	23.2	101.42	0.04
2021/5/23 23:50	0.658	23.2	101.44	0.04
2021/5/24 0:00	0.658	23.2	101.44	0.04
2021/5/24 0:10	0.655	23.2	101.44	0.04
2021/5/24 0:20	0.655	23.2	101.44	0.04
2021/5/24 0:30	0.655	23.2	101.42	0.04
2021/5/24 0:40	0.655	23.2	101.44	0.04
2021/5/24 0:50	0.655	23.2	101.44	0.04
2021/5/24 1:00	0.655	23.2	101.44	0.04
2021/5/24 1:10	0.655	23.2	101.44	0.04
2021/5/24 1:20	0.659	23.1	101.44	0.05
2021/5/24 1:30	0.655	23.1	101.42	0.05
2021/5/24 1:40	0.655	23.1	101.42	0.04
2021/5/24 1:50	0.655	23.1	101.41	0.05
2021/5/24 2:00	0.655	23.1	101.39	0.05
2021/5/24 2:10	0.651	23.1	101.38	0.04
2021/5/24 2:20	0.651	23.1	101.36	0.05
2021/5/24 2:30	0.655	23.0	101.35	0.04
2021/5/24 2:40	0.655	23.0	101.35	0.04
2021/5/24 2:50	0.655	23.0	101.34	0.05
2021/5/24 3:00	0.655	23.0	101.34	0.05
2021/5/24 3:10	0.652	23.0	101.31	0.04
2021/5/24 3:20	0.652	23.0	101.31	0.05
2021/5/24 3:30	0.652	23.0	101.32	0.04
2021/5/24 3:40	0.652	23.0	101.32	0.04
2021/5/24 3:50	0.652	23.0	101.32	0.04
2021/5/24 4:00	0.648	23.0	101.32	0.04
2021/5/24 4:10	0.648	23.0	101.34	0.04
2021/5/24 4:20	0.648	23.0	101.35	0.04
2021/5/24 4:30	0.648	23.0	101.35	0.03
2021/5/24 4:40	0.645	23.0	101.34	0.04
2021/5/24 4:50	0.645	23.0	101.34	0.04
2021/5/24 5:00	0.645	23.0	101.34	0.04
2021/5/24 5:10	0.645	23.0	101.35	0.04
2021/5/24 5:20	0.645	23.0	101.36	0.04
2021/5/24 5:30	0.641	23.0	101.36	0.04
2021/5/24 5:40	0.637	23.1	101.36	0.04
2021/5/24 5:50	0.637	23.1	101.36	0.03
2021/5/24 6:00	0.637	23.1	101.38	0.04
2021/5/24 6:10	0.637	23.1	101.38	0.03
2021/5/24 6:20	0.637	23.1	101.39	0.04
2021/5/24 6:30	0.637	23.1	101.41	0.04
2021/5/24 6:40	0.637	23.1	101.42	0.03
2021/5/24 6:50	0.633	23.2	101.44	0.03
2021/5/24 7:00	0.633	23.2	101.45	0.03
2021/5/24 7:10	0.633	23.2	101.45	0.03
2021/5/24 7:20	0.630	23.2	101.44	0.03
2021/5/24 7:30	0.630	23.2	101.45	0.03
2021/5/24 7:40	0.630	23.2	101.45	0.04
2021/5/24 7:50	0.626	23.3	101.47	0.04
2021/5/24 8:00	0.626	23.3	101.47	0.04
2021/5/24 8:10	0.630	23.3	101.48	0.04
2021/5/24 8:20	0.630	23.3	101.50	0.04
2021/5/24 8:30	0.626	23.4	101.50	0.04
2021/5/24 8:40	0.626	23.4	101.48	0.04

2021/5/24 8:50	0.626	23.4	101.47	0.04
2021/5/24 9:00	0.626	23.4	101.47	0.04
2021/5/24 9:10	0.626	23.4	101.45	0.03
2021/5/24 9:20	0.626	23.4	101.45	0.03
2021/5/24 9:30	0.622	23.5	101.47	0.03
2021/5/24 9:40	0.622	23.5	101.47	0.03
2021/5/24 9:50	0.622	23.5	101.47	0.03
2021/5/24 10:00	0.622	23.5	101.47	0.03
2021/5/24 10:10	0.626	23.5	101.47	0.03
2021/5/24 10:20	0.626	23.5	101.47	0.03
2021/5/24 10:30	0.626	23.5	101.47	0.03
2021/5/24 10:40	0.629	23.5	101.47	0.03
2021/5/24 10:50	0.629	23.5	101.48	0.03
2021/5/24 11:00	0.632	23.5	101.47	0.03
2021/5/24 11:10	0.629	23.6	101.47	0.03
2021/5/24 11:20	0.629	23.6	101.45	0.03
2021/5/24 11:30	0.632	23.6	101.44	0.04
2021/5/24 11:40	0.632	23.6	101.42	0.03
2021/5/24 11:50	0.635	23.6	101.41	0.04
2021/5/24 12:00	0.635	23.6	101.39	0.04
2021/5/24 12:10	0.639	23.6	101.38	0.04
2021/5/24 12:20	0.635	23.7	101.38	0.04
2021/5/24 12:30	0.639	23.7	101.36	0.04
2021/5/24 12:40	0.639	23.7	101.34	0.04
2021/5/24 12:50	0.639	23.7	101.34	0.05
2021/5/24 13:00	0.642	23.7	101.32	0.04
2021/5/24 13:10	0.642	23.7	101.31	0.04
2021/5/24 13:20	0.638	23.8	101.29	0.05
2021/5/24 13:30	0.641	23.8	101.29	0.05
2021/5/24 13:40	0.655	23.8	101.31	0.18
2021/5/24 13:50	0.670	24.1	101.32	0.21
2021/5/24 14:00	0.668	24.4	101.31	0.19
2021/5/24 14:10	0.670	24.5	101.31	0.18
2021/5/24 14:20	0.670	24.6	101.29	0.19
2021/5/24 14:30	0.670	24.6	101.29	0.18
2021/5/24 14:40	0.666	24.7	101.29	0.17
2021/5/24 14:50	0.665	24.8	101.29	0.14
2021/5/24 15:00	0.664	24.9	101.28	0.08
2021/5/24 15:10	0.655	24.9	101.28	0.04
2021/5/24 15:20	0.648	24.9	101.29	0.05
2021/5/24 15:30	0.649	24.8	101.28	0.07
2021/5/24 15:40	0.653	24.7	101.29	0.07
2021/5/24 15:50	0.646	24.7	101.29	0.05
2021/5/24 16:00	0.646	24.7	101.28	0.05
2021/5/24 16:10	0.640	24.7	101.28	0.05
2021/5/24 16:20	0.637	24.7	101.26	0.05
2021/5/24 16:30	0.637	24.7	101.26	0.06
2021/5/24 16:40	0.633	24.7	101.26	0.04
2021/5/24 16:50	0.628	24.6	101.26	0.05
2021/5/24 17:00	0.637	24.6	101.26	0.05
2021/5/24 17:10	0.644	24.6	101.25	0.04
2021/5/24 17:20	0.644	24.5	101.25	0.04
2021/5/24 17:30	0.648	24.4	101.25	0.03
2021/5/24 17:40	0.649	24.3	101.25	0.03
2021/5/24 17:50	0.653	24.2	101.23	0.03
2021/5/24 18:00	0.646	24.2	101.23	0.03

2021/5/24 18:10	0.650	24.1	101.25	0.03
2021/5/24 18:20	0.651	24.0	101.26	0.03
2021/5/24 18:30	0.647	24.0	101.26	0.03
2021/5/24 18:40	0.648	23.9	101.26	0.03
2021/5/24 18:50	0.648	23.9	101.28	0.04
2021/5/24 19:00	0.644	23.9	101.28	0.03
2021/5/24 19:10	0.648	23.8	101.29	0.03
2021/5/24 19:20	0.645	23.8	101.29	0.03
2021/5/24 19:30	0.645	23.8	101.31	0.03
2021/5/24 19:40	0.641	23.8	101.31	0.04
2021/5/24 19:50	0.645	23.7	101.31	0.04
2021/5/24 20:00	0.642	23.7	101.29	0.04
2021/5/24 20:10	0.642	23.7	101.29	0.04
2021/5/24 20:20	0.642	23.7	101.28	0.04
2021/5/24 20:30	0.639	23.7	101.28	0.04
2021/5/24 20:40	0.639	23.7	101.28	0.05
2021/5/24 20:50	0.642	23.6	101.26	0.05
2021/5/24 21:00	0.642	23.6	101.28	0.04
2021/5/24 21:10	0.642	23.6	101.29	0.04
2021/5/24 21:20	0.642	23.6	101.29	0.05
2021/5/24 21:30	0.642	23.6	101.31	0.05
2021/5/24 21:40	0.639	23.6	101.32	0.05
2021/5/24 21:50	0.639	23.6	101.32	0.05
2021/5/24 22:00	0.639	23.6	101.34	0.05
2021/5/24 22:10	0.635	23.6	101.34	0.05
2021/5/24 22:20	0.635	23.6	101.32	0.05
2021/5/24 22:30	0.632	23.6	101.31	0.05
2021/5/24 22:40	0.632	23.6	101.29	0.05
2021/5/24 22:50	0.629	23.6	101.26	0.05
2021/5/24 23:00	0.632	23.5	101.23	0.05
2021/5/24 23:10	0.629	23.5	101.23	0.05
2021/5/24 23:20	0.629	23.5	101.22	0.05
2021/5/24 23:30	0.629	23.5	101.22	0.05
2021/5/24 23:40	0.629	23.5	101.20	0.05
2021/5/24 23:50	0.629	23.5	101.20	0.05
2021/5/25 0:00	0.626	23.5	101.19	0.05
2021/5/25 0:10	0.626	23.5	101.17	0.06
2021/5/25 0:20	0.626	23.5	101.17	0.05
2021/5/25 0:30	0.626	23.5	101.17	0.05
2021/5/25 0:40	0.626	23.5	101.19	0.05
2021/5/25 0:50	0.626	23.5	101.17	0.05
2021/5/25 1:00	0.626	23.5	101.19	0.05
2021/5/25 1:10	0.626	23.5	101.19	0.05
2021/5/25 1:20	0.626	23.5	101.16	0.06
2021/5/25 1:30	0.626	23.5	101.14	0.05
2021/5/25 1:40	0.626	23.5	101.14	0.05
2021/5/25 1:50	0.629	23.5	101.17	0.05
2021/5/25 2:00	0.629	23.5	101.19	0.05
2021/5/25 2:10	0.633	23.4	101.19	0.05
2021/5/25 2:20	0.633	23.4	101.17	0.06
2021/5/25 2:30	0.633	23.4	101.17	0.05
2021/5/25 2:40	0.633	23.4	101.17	0.05
2021/5/25 2:50	0.636	23.4	101.16	0.05
2021/5/25 3:00	0.636	23.4	101.16	0.05
2021/5/25 3:10	0.636	23.4	101.16	0.06
2021/5/25 3:20	0.636	23.4	101.17	0.05

2021/5/25 3:30	0.636	23.4	101.17	0.05
2021/5/25 3:40	0.636	23.4	101.17	0.05
2021/5/25 3:50	0.636	23.4	101.17	0.05
2021/5/25 4:00	0.636	23.4	101.16	0.05
2021/5/25 4:10	0.636	23.4	101.13	0.05
2021/5/25 4:20	0.636	23.4	101.13	0.05
2021/5/25 4:30	0.636	23.4	101.12	0.05
2021/5/25 4:40	0.636	23.4	101.10	0.05
2021/5/25 4:50	0.636	23.4	101.10	0.05
2021/5/25 5:00	0.636	23.4	101.09	0.05
2021/5/25 5:10	0.636	23.4	101.10	0.05
2021/5/25 5:20	0.636	23.4	101.13	0.05
2021/5/25 5:30	0.636	23.4	101.13	0.05
2021/5/25 5:40	0.636	23.4	101.12	0.06
2021/5/25 5:50	0.640	23.4	101.14	0.06
2021/5/25 6:00	0.640	23.4	101.13	0.05
2021/5/25 6:10	0.640	23.4	101.12	0.06
2021/5/25 6:20	0.640	23.4	101.10	0.06
2021/5/25 6:30	0.640	23.4	101.09	0.05
2021/5/25 6:40	0.640	23.4	101.07	0.05
2021/5/25 6:50	0.640	23.4	101.07	0.06
2021/5/25 7:00	0.640	23.4	101.07	0.05
2021/5/25 7:10	0.640	23.4	101.04	0.06
2021/5/25 7:20	0.640	23.4	101.04	0.05
2021/5/25 7:30	0.636	23.4	101.04	0.06
2021/5/25 7:40	0.636	23.4	101.04	0.05
2021/5/25 7:50	0.636	23.4	101.04	0.05
2021/5/25 8:00	0.636	23.4	101.04	0.05
2021/5/25 8:10	0.636	23.4	101.03	0.05
2021/5/25 8:20	0.636	23.4	101.03	0.05
2021/5/25 8:30	0.636	23.4	101.00	0.05
2021/5/25 8:40	0.636	23.4	100.98	0.05
2021/5/25 8:50	0.636	23.4	100.95	0.05
2021/5/25 9:00	0.632	23.5	100.95	0.05
2021/5/25 9:10	0.632	23.5	100.94	0.05
2021/5/25 9:20	0.632	23.5	100.95	0.05
2021/5/25 9:30	0.636	23.5	100.92	0.04
2021/5/25 9:40	0.636	23.5	100.90	0.04
2021/5/25 9:50	0.632	23.6	100.87	0.04
2021/5/25 10:00	0.632	23.6	100.85	0.04
2021/5/25 10:10	0.632	23.6	100.84	0.03
2021/5/25 10:20	0.632	23.7	100.82	0.03
2021/5/25 10:30	0.632	23.7	100.82	0.03
2021/5/25 10:40	0.632	23.7	100.82	0.04
2021/5/25 10:50	0.628	23.8	100.81	0.04
2021/5/25 11:00	0.628	23.8	100.81	0.05
2021/5/25 11:10	0.624	23.8	100.81	0.05
2021/5/25 11:20	0.628	23.8	100.81	0.05
2021/5/25 11:30	0.617	23.9	100.79	0.05
2021/5/25 11:40	0.610	24.0	100.81	0.05
2021/5/25 11:50	0.603	24.1	100.82	0.06
2021/5/25 12:00	0.603	24.1	100.81	0.06
2021/5/25 12:10	0.600	24.2	100.79	0.06
2021/5/25 12:20	0.596	24.3	100.79	0.06
2021/5/25 12:30	0.593	24.3	100.79	0.06
2021/5/25 12:40	0.589	24.4	100.79	0.05

2021/5/25 12:50	0.586	24.5	100.79	0.06
2021/5/25 13:00	0.583	24.5	100.79	0.05
2021/5/25 13:10	0.586	24.5	100.78	0.05
2021/5/25 13:20	0.582	24.6	100.75	0.05
2021/5/25 13:30	0.582	24.7	100.72	0.05
2021/5/25 13:40	0.582	24.7	100.72	0.06
2021/5/25 13:50	0.579	24.8	100.75	0.05
2021/5/25 14:00	0.579	24.8	100.73	0.05
2021/5/25 14:10	0.579	24.8	100.70	0.06
2021/5/25 14:20	0.575	24.8	100.68	0.05
2021/5/25 14:30	0.579	24.8	100.65	0.06
2021/5/25 14:40	0.579	24.8	100.63	0.05
2021/5/25 14:50	0.582	24.8	100.62	0.05
2021/5/25 15:00	0.585	24.8	100.60	0.05
2021/5/25 15:10	0.588	24.8	100.59	0.05
2021/5/25 15:20	0.585	24.9	100.54	0.05
2021/5/25 15:30	0.588	24.9	100.51	0.05
2021/5/25 15:40	0.588	24.9	100.50	0.05
2021/5/25 15:50	0.591	24.9	100.49	0.05
2021/5/25 16:00	0.591	24.9	100.47	0.05
2021/5/25 16:10	0.591	24.9	100.46	0.05
2021/5/25 16:20	0.591	25.0	100.43	0.05
2021/5/25 16:30	0.591	25.0	100.40	0.05
2021/5/25 16:40	0.591	25.0	100.40	0.06
2021/5/25 16:50	0.594	25.0	100.40	0.06
2021/5/25 17:00	0.600	25.0	100.37	0.06
2021/5/25 17:10	0.600	25.1	100.37	0.05
2021/5/25 17:20	0.606	25.1	100.35	0.06
2021/5/25 17:30	0.612	25.1	100.32	0.07
2021/5/25 17:40	0.615	25.1	100.31	0.07
2021/5/25 17:50	0.619	25.1	100.28	0.07
2021/5/25 18:00	0.622	25.1	100.25	0.07
2021/5/25 18:10	0.621	25.2	100.25	0.08
2021/5/25 18:20	0.624	25.2	100.24	0.07
2021/5/25 18:30	0.630	25.2	100.24	0.07
2021/5/25 18:40	0.630	25.2	100.24	0.05
2021/5/25 18:50	0.635	25.0	100.22	0.04
2021/5/25 19:00	0.636	24.9	100.21	0.03
2021/5/25 19:10	0.636	24.8	100.24	0.04
2021/5/25 19:20	0.641	24.6	100.28	0.04
2021/5/25 19:30	0.641	24.5	100.29	0.04
2021/5/25 19:40	0.645	24.4	100.25	0.04
2021/5/25 19:50	0.646	24.3	100.24	0.04
2021/5/25 20:00	0.649	24.2	100.24	0.03
2021/5/25 20:10	0.649	24.2	100.21	0.03
2021/5/25 20:20	0.653	24.1	100.16	0.03
2021/5/25 20:30	0.653	24.1	100.09	0.03
2021/5/25 20:40	0.657	24.0	100.05	0.03
2021/5/25 20:50	0.657	24.0	100.02	0.03
2021/5/25 21:00	0.661	23.9	100.05	0.03
2021/5/25 21:10	0.661	23.9	100.02	0.03
2021/5/25 21:20	0.661	23.9	99.99	0.04
2021/5/25 21:30	0.661	23.9	99.96	0.03
2021/5/25 21:40	0.658	23.9	99.96	0.04
2021/5/25 21:50	0.658	23.9	99.94	0.04
2021/5/25 22:00	0.662	23.8	99.90	0.04

2021/5/25 22:10	0.662	23.8	99.85	0.04
2021/5/25 22:20	0.662	23.8	99.84	0.03
2021/5/25 22:30	0.662	23.8	99.81	0.03
2021/5/25 22:40	0.662	23.8	99.81	0.04
2021/5/25 22:50	0.662	23.8	99.81	0.03
2021/5/25 23:00	0.662	23.8	99.80	0.03
2021/5/25 23:10	0.662	23.8	99.80	0.03
2021/5/25 23:20	0.662	23.8	99.78	0.04
2021/5/25 23:30	0.665	23.8	99.77	0.03
2021/5/25 23:40	0.665	23.8	99.74	0.04
2021/5/25 23:50	0.665	23.8	99.71	0.04
2021/5/26 0:00	0.665	23.8	99.69	0.04
2021/5/26 0:10	0.669	23.8	99.64	0.04
2021/5/26 0:20	0.669	23.8	99.65	0.04
2021/5/26 0:30	0.669	23.8	99.66	0.04
2021/5/26 0:40	0.672	23.8	99.61	0.03
2021/5/26 0:50	0.672	23.8	99.58	0.03
2021/5/26 1:00	0.675	23.8	99.53	0.03
2021/5/26 1:10	0.675	23.8	99.55	0.04
2021/5/26 1:20	0.675	23.8	99.56	0.03
2021/5/26 1:30	0.675	23.8	99.53	0.03
2021/5/26 1:40	0.679	23.8	99.59	0.04
2021/5/26 1:50	0.679	23.8	99.58	0.04
2021/5/26 2:00	0.679	23.8	99.56	0.04
2021/5/26 2:10	0.682	23.8	99.49	0.03
2021/5/26 2:20	0.682	23.8	99.49	0.03
2021/5/26 2:30	0.682	23.8	99.53	0.04
2021/5/26 2:40	0.686	23.8	99.47	0.04
2021/5/26 2:50	0.686	23.8	99.43	0.04
2021/5/26 3:00	0.686	23.8	99.43	0.04
2021/5/26 3:10	0.689	23.8	99.42	0.04
2021/5/26 3:20	0.689	23.8	99.43	0.04
2021/5/26 3:30	0.689	23.8	99.47	0.04
2021/5/26 3:40	0.692	23.8	99.49	0.04
2021/5/26 3:50	0.692	23.8	99.46	0.04
2021/5/26 4:00	0.692	23.8	99.44	0.04
2021/5/26 4:10	0.692	23.8	99.44	0.04
2021/5/26 4:20	0.696	23.8	99.44	0.04
2021/5/26 4:30	0.696	23.8	99.47	0.04
2021/5/26 4:40	0.696	23.8	99.46	0.04
2021/5/26 4:50	0.696	23.8	99.47	0.04
2021/5/26 5:00	0.696	23.8	99.47	0.04
2021/5/26 5:10	0.696	23.8	99.47	0.04
2021/5/26 5:20	0.699	23.8	99.47	0.04
2021/5/26 5:30	0.699	23.8	99.50	0.04
2021/5/26 5:40	0.699	23.8	99.49	0.04
2021/5/26 5:50	0.699	23.8	99.52	0.04
2021/5/26 6:00	0.699	23.8	99.56	0.04
2021/5/26 6:10	0.699	23.8	99.59	0.04
2021/5/26 6:20	0.703	23.8	99.61	0.04
2021/5/26 6:30	0.698	23.9	99.59	0.04
2021/5/26 6:40	0.698	24.0	99.59	0.05
2021/5/26 6:50	0.697	24.1	99.58	0.04
2021/5/26 7:00	0.692	24.3	99.59	0.04
2021/5/26 7:10	0.691	24.4	99.64	0.05
2021/5/26 7:20	0.690	24.5	99.62	0.05

2021/5/26 7:30	0.685	24.7	99.64	0.05
2021/5/26 7:40	0.681	24.8	99.65	0.06
2021/5/26 7:50	0.681	24.8	99.68	0.05
2021/5/26 8:00	0.683	24.9	99.72	0.06
2021/5/26 8:10	0.683	24.9	99.75	0.06
2021/5/26 8:20	0.676	25.0	99.75	0.05
2021/5/26 8:30	0.682	25.0	99.75	0.07
2021/5/26 8:40	0.682	25.0	99.77	0.06
2021/5/26 8:50	0.675	25.1	99.78	0.06
2021/5/26 9:00	0.678	25.1	99.80	0.06
2021/5/26 9:10	0.675	25.1	99.81	0.06
2021/5/26 9:20	0.675	25.1	99.81	0.07
2021/5/26 9:30	0.675	25.1	99.84	0.07
2021/5/26 9:40	0.675	25.1	99.87	0.06
2021/5/26 9:50	0.675	25.1	99.87	0.07
2021/5/26 10:00	0.675	25.1	99.85	0.07
2021/5/26 10:10	0.671	25.2	99.85	0.08
2021/5/26 10:20	0.668	25.2	99.85	0.07
2021/5/26 10:30	0.668	25.2	99.88	0.07
2021/5/26 10:40	0.671	25.2	99.90	0.08
2021/5/26 10:50	0.668	25.2	99.88	0.08
2021/5/26 11:00	0.668	25.2	99.90	0.07
2021/5/26 11:10	0.668	25.2	99.90	0.08
2021/5/26 11:20	0.668	25.2	99.91	0.07
2021/5/26 11:30	0.668	25.2	99.90	0.07
2021/5/26 11:40	0.665	25.2	99.90	0.07
2021/5/26 11:50	0.665	25.2	99.90	0.07
2021/5/26 12:00	0.668	25.2	99.90	0.08
2021/5/26 12:10	0.665	25.2	99.90	0.07
2021/5/26 12:20	0.668	25.2	99.90	0.08
2021/5/26 12:30	0.668	25.2	99.93	0.07
2021/5/26 12:40	0.668	25.2	99.93	0.08
2021/5/26 12:50	0.665	25.2	99.94	0.07
2021/5/26 13:00	0.665	25.2	99.93	0.08
2021/5/26 13:10	0.665	25.2	99.91	0.08
2021/5/26 13:20	0.659	25.2	99.93	0.07
2021/5/26 13:30	0.662	25.2	99.90	0.07
2021/5/26 13:40	0.659	25.2	99.93	0.07
2021/5/26 13:50	0.659	25.2	99.93	0.06
2021/5/26 14:00	0.659	25.2	99.93	0.07
2021/5/26 14:10	0.655	25.2	99.93	0.07
2021/5/26 14:20	0.659	25.2	99.93	0.07
2021/5/26 14:30	0.659	25.2	99.96	0.07
2021/5/26 14:40	0.655	25.2	99.96	0.07
2021/5/26 14:50	0.655	25.3	99.97	0.07
2021/5/26 15:00	0.652	25.3	99.97	0.07
2021/5/26 15:10	0.652	25.3	99.97	0.06
2021/5/26 15:20	0.652	25.3	99.99	0.07
2021/5/26 15:30	0.652	25.3	100.00	0.06
2021/5/26 15:40	0.648	25.3	100.00	0.07
2021/5/26 15:50	0.648	25.3	100.02	0.07
2021/5/26 16:00	0.652	25.2	100.02	0.07
2021/5/26 16:10	0.652	25.2	100.02	0.07
2021/5/26 16:20	0.652	25.2	100.03	0.06
2021/5/26 16:30	0.648	25.3	100.03	0.06
2021/5/26 16:40	0.648	25.3	100.03	0.07

2021/5/26 16:50	0.645	25.3	100.05	0.06
2021/5/26 17:00	0.645	25.3	100.05	0.08
2021/5/26 17:10	0.642	25.3	100.06	0.06
2021/5/26 17:20	0.639	25.3	100.09	0.06
2021/5/26 17:30	0.639	25.3	100.07	0.06
2021/5/26 17:40	0.639	25.3	100.07	0.07
2021/5/26 17:50	0.639	25.3	100.06	0.07
2021/5/26 18:00	0.636	25.3	100.06	0.06
2021/5/26 18:10	0.636	25.3	100.07	0.06
2021/5/26 18:20	0.633	25.3	100.07	0.06
2021/5/26 18:30	0.643	25.2	100.09	0.07
2021/5/26 18:40	0.643	25.2	100.09	0.07
2021/5/26 18:50	0.644	25.1	100.10	0.06
2021/5/26 19:00	0.644	25.0	100.10	0.05
2021/5/26 19:10	0.648	24.9	100.10	0.05
2021/5/26 19:20	0.649	24.8	100.12	0.05
2021/5/26 19:30	0.653	24.7	100.13	0.04
2021/5/26 19:40	0.653	24.7	100.13	0.05
2021/5/26 19:50	0.653	24.6	100.13	0.05
2021/5/26 20:00	0.653	24.6	100.13	0.05
2021/5/26 20:10	0.657	24.5	100.15	0.05
2021/5/26 20:20	0.657	24.5	100.16	0.05
2021/5/26 20:30	0.657	24.5	100.16	0.05
2021/5/26 20:40	0.661	24.4	100.16	0.04
2021/5/26 20:50	0.661	24.4	100.16	0.04
2021/5/26 21:00	0.661	24.4	100.19	0.05
2021/5/26 21:10	0.665	24.3	100.21	0.05
2021/5/26 21:20	0.665	24.3	100.21	0.04
2021/5/26 21:30	0.665	24.3	100.21	0.04
2021/5/26 21:40	0.662	24.3	100.22	0.04
2021/5/26 21:50	0.666	24.2	100.22	0.04
2021/5/26 22:00	0.666	24.2	100.24	0.04
2021/5/26 22:10	0.666	24.2	100.24	0.04
2021/5/26 22:20	0.666	24.2	100.24	0.04
2021/5/26 22:30	0.663	24.2	100.24	0.04
2021/5/26 22:40	0.667	24.1	100.24	0.03
2021/5/26 22:50	0.667	24.1	100.24	0.04
2021/5/26 23:00	0.667	24.1	100.24	0.04
2021/5/26 23:10	0.663	24.1	100.24	0.03
2021/5/26 23:20	0.663	24.1	100.24	0.04
2021/5/26 23:30	0.667	24.0	100.25	0.03
2021/5/26 23:40	0.664	24.0	100.24	0.04
2021/5/26 23:50	0.664	24.0	100.22	0.04
2021/5/27 0:00	0.664	24.0	100.22	0.04
2021/5/27 0:10	0.661	24.0	100.21	0.04
2021/5/27 0:20	0.665	23.9	100.19	0.04
2021/5/27 0:30	0.665	23.9	100.19	0.05
2021/5/27 0:40	0.661	23.9	100.18	0.04
2021/5/27 0:50	0.661	23.9	100.19	0.04
2021/5/27 1:00	0.661	23.9	100.19	0.04
2021/5/27 1:10	0.658	23.9	100.18	0.04
2021/5/27 1:20	0.662	23.8	100.18	0.04
2021/5/27 1:30	0.662	23.8	100.16	0.04
2021/5/27 1:40	0.658	23.8	100.16	0.04
2021/5/27 1:50	0.658	23.8	100.16	0.05
2021/5/27 2:00	0.655	23.8	100.16	0.05

2021/5/27 2:10	0.655	23.8	100.18	0.05
2021/5/27 2:20	0.656	23.7	100.15	0.05
2021/5/27 2:30	0.656	23.7	100.16	0.04
2021/5/27 2:40	0.652	23.7	100.18	0.04
2021/5/27 2:50	0.649	23.7	100.16	0.05
2021/5/27 3:00	0.645	23.7	100.19	0.04
2021/5/27 3:10	0.649	23.6	100.18	0.04
2021/5/27 3:20	0.646	23.6	100.18	0.05
2021/5/27 3:30	0.642	23.6	100.21	0.05
2021/5/27 3:40	0.642	23.6	100.21	0.05
2021/5/27 3:50	0.639	23.6	100.21	0.05
2021/5/27 4:00	0.639	23.5	100.19	0.05
2021/5/27 4:10	0.639	23.5	100.22	0.05
2021/5/27 4:20	0.636	23.5	100.21	0.05
2021/5/27 4:30	0.632	23.5	100.24	0.05
2021/5/27 4:40	0.632	23.5	100.22	0.05
2021/5/27 4:50	0.633	23.4	100.21	0.05
2021/5/27 5:00	0.633	23.4	100.22	0.05
2021/5/27 5:10	0.629	23.4	100.21	0.05
2021/5/27 5:20	0.626	23.4	100.21	0.05
2021/5/27 5:30	0.626	23.4	100.22	0.05
2021/5/27 5:40	0.622	23.4	100.22	0.05
2021/5/27 5:50	0.626	23.3	100.22	0.05
2021/5/27 6:00	0.623	23.3	100.22	0.05
2021/5/27 6:10	0.623	23.3	100.22	0.05
2021/5/27 6:20	0.619	23.3	100.22	0.07
2021/5/27 6:30	0.619	23.3	100.22	0.06
2021/5/27 6:40	0.615	23.4	100.22	0.06
2021/5/27 6:50	0.615	23.5	100.21	0.06
2021/5/27 7:00	0.608	23.7	100.22	0.07
2021/5/27 7:10	0.604	23.8	100.25	0.07
2021/5/27 7:20	0.604	23.9	100.24	0.06
2021/5/27 7:30	0.600	24.0	100.22	0.07
2021/5/27 7:40	0.597	24.1	100.24	0.06
2021/5/27 7:50	0.593	24.2	100.25	0.06
2021/5/27 8:00	0.590	24.3	100.24	0.06
2021/5/27 8:10	0.590	24.3	100.22	0.07
2021/5/27 8:20	0.589	24.4	100.24	0.06
2021/5/27 8:30	0.589	24.4	100.25	0.06
2021/5/27 8:40	0.586	24.5	100.24	0.07
2021/5/27 8:50	0.586	24.5	100.25	0.07
2021/5/27 9:00	0.586	24.5	100.25	0.06
2021/5/27 9:10	0.586	24.5	100.22	0.07
2021/5/27 9:20	0.582	24.6	100.21	0.07
2021/5/27 9:30	0.586	24.6	100.21	0.07
2021/5/27 9:40	0.586	24.6	100.25	0.07
2021/5/27 9:50	0.582	24.7	100.25	0.06
2021/5/27 10:00	0.582	24.7	100.24	0.07
2021/5/27 10:10	0.585	24.7	100.25	0.06
2021/5/27 10:20	0.579	24.8	100.27	0.05
2021/5/27 10:30	0.582	24.8	100.27	0.06
2021/5/27 10:40	0.582	24.8	100.28	0.06
2021/5/27 10:50	0.578	24.9	100.28	0.05
2021/5/27 11:00	0.578	24.9	100.27	0.05
2021/5/27 11:10	0.581	24.9	100.25	0.06
2021/5/27 11:20	0.578	24.9	100.27	0.05

2021/5/27 11:30	0.578	24.9	100.28	0.04
2021/5/27 11:40	0.582	24.7	100.29	0.06
2021/5/27 11:50	0.586	24.6	100.28	0.05
2021/5/27 12:00	0.582	24.7	100.28	0.05
2021/5/27 12:10	0.582	24.7	100.27	0.05
2021/5/27 12:20	0.579	24.8	100.28	0.06
2021/5/27 12:30	0.579	24.8	100.24	0.05
2021/5/27 12:40	0.578	24.9	100.22	0.06
2021/5/27 12:50	0.575	24.9	100.22	0.05
2021/5/27 13:00	0.572	25.0	100.22	0.06
2021/5/27 13:10	0.572	25.0	100.24	0.05
2021/5/27 13:20	0.569	25.0	100.22	0.05
2021/5/27 13:30	0.565	25.1	100.22	0.05
2021/5/27 13:40	0.565	25.1	100.24	0.06
2021/5/27 13:50	0.565	25.1	100.24	0.06
2021/5/27 14:00	0.562	25.1	100.24	0.06
2021/5/27 14:10	0.559	25.1	100.24	0.06
2021/5/27 14:20	0.562	25.1	100.22	0.06
2021/5/27 14:30	0.559	25.2	100.22	0.05
2021/5/27 14:40	0.559	25.2	100.24	0.05
2021/5/27 14:50	0.559	25.2	100.24	0.05
2021/5/27 15:00	0.559	25.2	100.25	0.05
2021/5/27 15:10	0.559	25.2	100.24	0.06
2021/5/27 15:20	0.559	25.2	100.22	0.05
2021/5/27 15:30	0.559	25.2	100.22	0.05
2021/5/27 15:40	0.559	25.2	100.24	0.05
2021/5/27 15:50	0.559	25.2	100.24	0.05
2021/5/27 16:00	0.562	25.2	100.24	0.06
2021/5/27 16:10	0.562	25.2	100.25	0.05
2021/5/27 16:20	0.559	25.2	100.24	0.05
2021/5/27 16:30	0.559	25.2	100.22	0.05
2021/5/27 16:40	0.562	25.2	100.21	0.06
2021/5/27 16:50	0.562	25.2	100.18	0.05
2021/5/27 17:00	0.562	25.2	100.18	0.05
2021/5/27 17:10	0.562	25.2	100.16	0.05
2021/5/27 17:20	0.562	25.2	100.18	0.05
2021/5/27 17:30	0.562	25.2	100.18	0.05
2021/5/27 17:40	0.562	25.2	100.16	0.05
2021/5/27 17:50	0.559	25.2	100.16	0.05
2021/5/27 18:00	0.562	25.2	100.15	0.05
2021/5/27 18:10	0.562	25.2	100.16	0.05
2021/5/27 18:20	0.562	25.2	100.18	0.05
2021/5/27 18:30	0.565	25.2	100.21	0.06
2021/5/27 18:40	0.568	25.1	100.19	0.06
2021/5/27 18:50	0.569	25.0	100.18	0.06
2021/5/27 19:00	0.572	24.9	100.18	0.05
2021/5/27 19:10	0.575	24.8	100.18	0.05
2021/5/27 19:20	0.576	24.7	100.18	0.05
2021/5/27 19:30	0.576	24.7	100.18	0.05
2021/5/27 19:40	0.579	24.6	100.21	0.06
2021/5/27 19:50	0.583	24.5	100.19	0.05
2021/5/27 20:00	0.583	24.5	100.19	0.05
2021/5/27 20:10	0.583	24.5	100.19	0.06
2021/5/27 20:20	0.586	24.4	100.21	0.05
2021/5/27 20:30	0.586	24.4	100.22	0.05
2021/5/27 20:40	0.586	24.4	100.22	0.04

2021/5/27 20:50	0.590	24.3	100.22	0.04
2021/5/27 21:00	0.590	24.3	100.24	0.04
2021/5/27 21:10	0.590	24.3	100.25	0.04
2021/5/27 21:20	0.590	24.3	100.25	0.04
2021/5/27 21:30	0.590	24.2	100.24	0.04
2021/5/27 21:40	0.590	24.2	100.25	0.04
2021/5/27 21:50	0.590	24.2	100.25	0.04
2021/5/27 22:00	0.590	24.2	100.25	0.04
2021/5/27 22:10	0.590	24.2	100.27	0.05
2021/5/27 22:20	0.590	24.2	100.28	0.04
2021/5/27 22:30	0.593	24.1	100.27	0.04
2021/5/27 22:40	0.593	24.1	100.28	0.04
2021/5/27 22:50	0.590	24.1	100.29	0.04
2021/5/27 23:00	0.590	24.1	100.31	0.05
2021/5/27 23:10	0.590	24.1	100.31	0.04
2021/5/27 23:20	0.590	24.1	100.31	0.04
2021/5/27 23:30	0.587	24.1	100.29	0.04
2021/5/27 23:40	0.590	24.0	100.29	0.05
2021/5/27 23:50	0.587	24.0	100.29	0.05
2021/5/28 0:00	0.587	24.0	100.29	0.04
2021/5/28 0:10	0.583	24.0	100.32	0.05
2021/5/28 0:20	0.583	24.0	100.32	0.05
2021/5/28 0:30	0.580	24.0	100.32	0.04
2021/5/28 0:40	0.580	24.0	100.29	0.05
2021/5/28 0:50	0.580	23.9	100.31	0.04
2021/5/28 1:00	0.577	23.9	100.31	0.05
2021/5/28 1:10	0.577	23.9	100.31	0.05
2021/5/28 1:20	0.574	23.9	100.31	0.04
2021/5/28 1:30	0.570	23.9	100.31	0.04
2021/5/28 1:40	0.574	23.8	100.31	0.05
2021/5/28 1:50	0.570	23.8	100.31	0.05
2021/5/28 2:00	0.570	23.8	100.31	0.05
2021/5/28 2:10	0.567	23.8	100.31	0.04
2021/5/28 2:20	0.567	23.8	100.31	0.05
2021/5/28 2:30	0.567	23.8	100.31	0.04
2021/5/28 2:40	0.563	23.8	100.31	0.04
2021/5/28 2:50	0.563	23.8	100.32	0.05
2021/5/28 3:00	0.560	23.8	100.37	0.05
2021/5/28 3:10	0.560	23.8	100.37	0.05
2021/5/28 3:20	0.557	23.8	100.38	0.05
2021/5/28 3:30	0.557	23.7	100.40	0.05
2021/5/28 3:40	0.557	23.7	100.43	0.05
2021/5/28 3:50	0.553	23.7	100.41	0.05
2021/5/28 4:00	0.553	23.7	100.43	0.05
2021/5/28 4:10	0.553	23.7	100.41	0.05
2021/5/28 4:20	0.550	23.7	100.41	0.05
2021/5/28 4:30	0.550	23.7	100.44	0.05
2021/5/28 4:40	0.550	23.7	100.47	0.05
2021/5/28 4:50	0.550	23.6	100.49	0.05
2021/5/28 5:00	0.550	23.6	100.50	0.05
2021/5/28 5:10	0.550	23.6	100.51	0.05
2021/5/28 5:20	0.550	23.6	100.53	0.05
2021/5/28 5:30	0.546	23.6	100.53	0.05
2021/5/28 5:40	0.546	23.6	100.54	0.05
2021/5/28 5:50	0.546	23.6	100.54	0.05
2021/5/28 6:00	0.546	23.6	100.54	0.06

2021/5/28 6:10	0.546	23.6	100.54	0.05
2021/5/28 6:20	0.549	23.5	100.53	0.06
2021/5/28 6:30	0.546	23.6	100.53	0.05
2021/5/28 6:40	0.543	23.7	100.53	0.04
2021/5/28 6:50	0.543	23.8	100.54	0.04
2021/5/28 7:00	0.540	23.9	100.56	0.04
2021/5/28 7:10	0.537	24.1	100.57	0.05
2021/5/28 7:20	0.537	24.2	100.57	0.05
2021/5/28 7:30	0.534	24.3	100.59	0.06
2021/5/28 7:40	0.534	24.4	100.59	0.05
2021/5/28 7:50	0.530	24.5	100.60	0.06
2021/5/28 8:00	0.530	24.5	100.60	0.06
2021/5/28 8:10	0.531	24.6	100.62	0.06
2021/5/28 8:20	0.531	24.6	100.65	0.06
2021/5/28 8:30	0.527	24.7	100.65	0.06
2021/5/28 8:40	0.527	24.7	100.65	0.06
2021/5/28 8:50	0.527	24.8	100.65	0.05
2021/5/28 9:00	0.527	24.8	100.65	0.05
2021/5/28 9:10	0.527	24.8	100.65	0.05
2021/5/28 9:20	0.527	24.9	100.65	0.05
2021/5/28 9:30	0.527	24.9	100.65	0.04
2021/5/28 9:40	0.531	24.9	100.63	0.05
2021/5/28 9:50	0.527	24.9	100.65	0.05
2021/5/28 10:00	0.531	24.9	100.65	0.04
2021/5/28 10:10	0.527	25.0	100.65	0.05
2021/5/28 10:20	0.527	25.0	100.65	0.05
2021/5/28 10:30	0.527	25.0	100.66	0.04
2021/5/28 10:40	0.527	25.0	100.66	0.05
2021/5/28 10:50	0.524	25.1	100.66	0.05
2021/5/28 11:00	0.521	25.1	100.68	0.04
2021/5/28 11:10	0.521	25.1	100.68	0.05
2021/5/28 11:20	0.518	25.2	100.68	0.05
2021/5/28 11:30	0.518	25.2	100.70	0.04
2021/5/28 11:40	0.518	25.2	100.70	0.05
2021/5/28 11:50	0.518	25.2	100.72	0.05
2021/5/28 12:00	0.515	25.2	100.72	0.05
2021/5/28 12:10	0.515	25.2	100.73	0.05
2021/5/28 12:20	0.515	25.2	100.73	0.04
2021/5/28 12:30	0.515	25.2	100.73	0.05
2021/5/28 12:40	0.515	25.3	100.73	0.04
2021/5/28 12:50	0.515	25.3	100.73	0.05
2021/5/28 13:00	0.515	25.3	100.73	0.05
2021/5/28 13:10	0.515	25.4	100.75	0.05
2021/5/28 13:20	0.518	25.4	100.73	0.05
2021/5/28 13:30	0.521	25.4	100.75	0.06
2021/5/28 13:40	0.521	25.4	100.73	0.05
2021/5/28 13:50	0.521	25.5	100.75	0.05
2021/5/28 14:00	0.521	25.5	100.75	0.05
2021/5/28 14:10	0.524	25.5	100.73	0.05
2021/5/28 14:20	0.524	25.5	100.73	0.05
2021/5/28 14:30	0.530	25.5	100.73	0.05
2021/5/28 14:40	0.530	25.5	100.75	0.05
2021/5/28 14:50	0.533	25.5	100.75	0.05
2021/5/28 15:00	0.533	25.5	100.73	0.05
2021/5/28 15:10	0.537	25.3	100.73	0.05
2021/5/28 15:20	0.543	25.2	100.73	0.05

2021/5/28 15:30	0.546	25.2	100.73	0.05
2021/5/28 15:40	0.546	25.3	100.73	0.05
2021/5/28 15:50	0.546	25.4	100.75	0.05
2021/5/28 16:00	0.552	25.4	100.75	0.05
2021/5/28 16:10	0.555	25.5	100.75	0.05
2021/5/28 16:20	0.558	25.5	100.76	0.05
2021/5/28 16:30	0.561	25.5	100.78	0.05
2021/5/28 16:40	0.567	25.5	100.79	0.05
2021/5/28 16:50	0.570	25.5	100.79	0.05
2021/5/28 17:00	0.576	25.5	100.81	0.05
2021/5/28 17:10	0.576	25.6	100.82	0.05
2021/5/28 17:20	0.582	25.6	100.84	0.05
2021/5/28 17:30	0.582	25.6	100.84	0.06
2021/5/28 17:40	0.582	25.6	100.85	0.05
2021/5/28 17:50	0.585	25.6	100.85	0.06
2021/5/28 18:00	0.582	25.6	100.85	0.05
2021/5/28 18:10	0.585	25.6	100.88	0.05
2021/5/28 18:20	0.585	25.6	100.88	0.05
2021/5/28 18:30	0.588	25.6	100.90	0.06
2021/5/28 18:40	0.589	25.5	100.91	0.06
2021/5/28 18:50	0.592	25.4	100.92	0.06
2021/5/28 19:00	0.596	25.3	100.92	0.06
2021/5/28 19:10	0.596	25.2	100.91	0.05
2021/5/28 19:20	0.599	25.2	100.91	0.05
2021/5/28 19:30	0.603	25.1	100.90	0.05
2021/5/28 19:40	0.603	25.1	100.90	0.05
2021/5/28 19:50	0.606	25.0	100.90	0.05
2021/5/28 20:00	0.603	25.0	100.90	0.04
2021/5/28 20:10	0.607	24.9	100.90	0.04
2021/5/28 20:20	0.607	24.9	100.88	0.04
2021/5/28 20:30	0.610	24.9	100.90	0.05
2021/5/28 20:40	0.610	24.9	100.88	0.04
2021/5/28 20:50	0.611	24.8	100.87	0.04
2021/5/28 21:00	0.614	24.8	100.85	0.04
2021/5/28 21:10	0.614	24.8	100.85	0.04
2021/5/28 21:20	0.614	24.8	100.85	0.04
2021/5/28 21:30	0.614	24.8	100.87	0.04
2021/5/28 21:40	0.614	24.8	100.90	0.04
2021/5/28 21:50	0.617	24.7	100.90	0.04
2021/5/28 22:00	0.617	24.7	100.90	0.04
2021/5/28 22:10	0.617	24.7	100.90	0.04
2021/5/28 22:20	0.617	24.7	100.90	0.04
2021/5/28 22:30	0.614	24.7	100.92	0.05
2021/5/28 22:40	0.614	24.7	100.95	0.05
2021/5/28 22:50	0.614	24.7	100.94	0.04
2021/5/28 23:00	0.614	24.7	100.97	0.04
2021/5/28 23:10	0.614	24.7	101.00	0.04
2021/5/28 23:20	0.618	24.6	101.01	0.04
2021/5/28 23:30	0.618	24.6	101.01	0.04
2021/5/28 23:40	0.618	24.6	101.01	0.04
2021/5/28 23:50	0.618	24.6	101.03	0.04
2021/5/29 0:00	0.618	24.6	101.03	0.05
2021/5/29 0:10	0.621	24.6	101.03	0.04
2021/5/29 0:20	0.621	24.6	101.03	0.04
2021/5/29 0:30	0.621	24.6	101.03	0.04
2021/5/29 0:40	0.621	24.6	101.03	0.05

2021/5/29 0:50	0.621	24.6	101.04	0.04
2021/5/29 1:00	0.624	24.6	101.06	0.04
2021/5/29 1:10	0.624	24.6	101.06	0.05
2021/5/29 1:20	0.624	24.6	101.06	0.04
2021/5/29 1:30	0.624	24.6	101.06	0.04
2021/5/29 1:40	0.628	24.6	101.04	0.04
2021/5/29 1:50	0.628	24.6	101.06	0.04
2021/5/29 2:00	0.628	24.6	101.04	0.04
2021/5/29 2:10	0.631	24.6	101.06	0.04
2021/5/29 2:20	0.631	24.6	101.06	0.04
2021/5/29 2:30	0.635	24.5	101.07	0.04
2021/5/29 2:40	0.635	24.5	101.09	0.04
2021/5/29 2:50	0.635	24.5	101.09	0.04
2021/5/29 3:00	0.631	24.5	101.09	0.04
2021/5/29 3:10	0.631	24.5	101.10	0.04
2021/5/29 3:20	0.631	24.5	101.12	0.04
2021/5/29 3:30	0.631	24.5	101.14	0.04
2021/5/29 3:40	0.628	24.5	101.16	0.04
2021/5/29 3:50	0.628	24.5	101.17	0.04
2021/5/29 4:00	0.628	24.5	101.19	0.04
2021/5/29 4:10	0.625	24.5	101.19	0.04
2021/5/29 4:20	0.625	24.5	101.20	0.04
2021/5/29 4:30	0.625	24.5	101.20	0.05
2021/5/29 4:40	0.625	24.4	101.22	0.04
2021/5/29 4:50	0.625	24.4	101.22	0.04
2021/5/29 5:00	0.625	24.4	101.22	0.05
2021/5/29 5:10	0.622	24.4	101.23	0.05
2021/5/29 5:20	0.622	24.4	101.25	0.05
2021/5/29 5:30	0.619	24.4	101.26	0.05
2021/5/29 5:40	0.619	24.4	101.26	0.05
2021/5/29 5:50	0.619	24.4	101.26	0.05
2021/5/29 6:00	0.615	24.4	101.25	0.04
2021/5/29 6:10	0.615	24.4	101.26	0.04
2021/5/29 6:20	0.615	24.4	101.26	0.04
2021/5/29 6:30	0.612	24.4	101.25	0.03
2021/5/29 6:40	0.612	24.5	101.25	0.04
2021/5/29 6:50	0.608	24.6	101.25	0.04
2021/5/29 7:00	0.605	24.7	101.25	0.04
2021/5/29 7:10	0.598	24.8	101.26	0.04
2021/5/29 7:20	0.597	24.9	101.29	0.03
2021/5/29 7:30	0.601	24.9	101.29	0.04
2021/5/29 7:40	0.597	25.0	101.29	0.04
2021/5/29 7:50	0.590	25.1	101.29	0.04
2021/5/29 8:00	0.590	25.1	101.28	0.04
2021/5/29 8:10	0.593	25.1	101.26	0.05
2021/5/29 8:20	0.590	25.2	101.26	0.04
2021/5/29 8:30	0.590	25.2	101.28	0.05
2021/5/29 8:40	0.587	25.2	101.26	0.05
2021/5/29 8:50	0.587	25.2	101.26	0.05
2021/5/29 9:00	0.587	25.2	101.25	0.05
2021/5/29 9:10	0.586	25.3	101.25	0.05
2021/5/29 9:20	0.586	25.3	101.25	0.05
2021/5/29 9:30	0.586	25.3	101.23	0.05
2021/5/29 9:40	0.586	25.4	101.23	0.05
2021/5/29 9:50	0.586	25.4	101.23	0.06
2021/5/29 10:00	0.586	25.4	101.23	0.06

2021/5/29 10:10	0.579	25.5	101.22	0.05
2021/5/29 10:20	0.583	25.5	101.22	0.07
2021/5/29 10:30	0.579	25.5	101.22	0.05
2021/5/29 10:40	0.583	25.5	101.22	0.06
2021/5/29 10:50	0.579	25.5	101.22	0.05
2021/5/29 11:00	0.583	25.5	101.22	0.06
2021/5/29 11:10	0.579	25.6	101.22	0.05
2021/5/29 11:20	0.579	25.6	101.22	0.06
2021/5/29 11:30	0.579	25.6	101.20	0.05
2021/5/29 11:40	0.579	25.6	101.22	0.06
2021/5/29 11:50	0.582	25.6	101.22	0.06
2021/5/29 12:00	0.579	25.6	101.22	0.06
2021/5/29 12:10	0.579	25.6	101.22	0.06
2021/5/29 12:20	0.579	25.6	101.22	0.05
2021/5/29 12:30	0.579	25.6	101.22	0.05
2021/5/29 12:40	0.579	25.6	101.22	0.06
2021/5/29 12:50	0.579	25.6	101.22	0.05
2021/5/29 13:00	0.582	25.6	101.22	0.05
2021/5/29 13:10	0.582	25.6	101.22	0.06
2021/5/29 13:20	0.582	25.6	101.22	0.05
2021/5/29 13:30	0.585	25.6	101.22	0.05
2021/5/29 13:40	0.585	25.6	101.22	0.06
2021/5/29 13:50	0.585	25.6	101.23	0.05
2021/5/29 14:00	0.585	25.6	101.25	0.05
2021/5/29 14:10	0.588	25.6	101.25	0.05
2021/5/29 14:20	0.583	25.5	101.25	0.06
2021/5/29 14:30	0.589	25.4	101.26	0.05
2021/5/29 14:40	0.592	25.4	101.26	0.05
2021/5/29 14:50	0.586	25.5	101.26	0.06
2021/5/29 15:00	0.586	25.5	101.26	0.05
2021/5/29 15:10	0.585	25.6	101.26	0.06
2021/5/29 15:20	0.588	25.6	101.26	0.06
2021/5/29 15:30	0.588	25.6	101.26	0.05
2021/5/29 15:40	0.588	25.7	101.26	0.06
2021/5/29 15:50	0.588	25.7	101.26	0.05
2021/5/29 16:00	0.588	25.7	101.26	0.06
2021/5/29 16:10	0.588	25.7	101.26	0.05
2021/5/29 16:20	0.588	25.7	101.26	0.05
2021/5/29 16:30	0.594	25.6	101.28	0.05
2021/5/29 16:40	0.591	25.7	101.28	0.06
2021/5/29 16:50	0.591	25.7	101.28	0.05
2021/5/29 17:00	0.594	25.7	101.29	0.04
2021/5/29 17:10	0.594	25.7	101.29	0.06
2021/5/29 17:20	0.597	25.7	101.29	0.05
2021/5/29 17:30	0.600	25.7	101.29	0.06
2021/5/29 17:40	0.603	25.7	101.31	0.05
2021/5/29 17:50	0.603	25.7	101.31	0.05
2021/5/29 18:00	0.603	25.7	101.31	0.04
2021/5/29 18:10	0.603	25.7	101.32	0.06
2021/5/29 18:20	0.606	25.7	101.32	0.05
2021/5/29 18:30	0.609	25.7	101.32	0.07
2021/5/29 18:40	0.613	25.6	101.32	0.07
2021/5/29 18:50	0.616	25.6	101.32	0.06
2021/5/29 19:00	0.619	25.5	101.32	0.07
2021/5/29 19:10	0.619	25.5	101.32	0.06
2021/5/29 19:20	0.619	25.5	101.32	0.06

2021/5/29 19:30	0.623	25.4	101.31	0.06
2021/5/29 19:40	0.623	25.4	101.31	0.07
2021/5/29 19:50	0.623	25.4	101.29	0.06
2021/5/29 20:00	0.623	25.4	101.31	0.07
2021/5/29 20:10	0.623	25.4	101.31	0.07
2021/5/29 20:20	0.627	25.3	101.31	0.07
2021/5/29 20:30	0.627	25.3	101.31	0.06
2021/5/29 20:40	0.627	25.3	101.32	0.06
2021/5/29 20:50	0.627	25.3	101.34	0.06
2021/5/29 21:00	0.627	25.3	101.35	0.07
2021/5/29 21:10	0.627	25.3	101.35	0.06
2021/5/29 21:20	0.630	25.2	101.36	0.06
2021/5/29 21:30	0.630	25.2	101.35	0.06
2021/5/29 21:40	0.634	25.2	101.36	0.06
2021/5/29 21:50	0.634	25.2	101.36	0.06
2021/5/29 22:00	0.634	25.2	101.36	0.06
2021/5/29 22:10	0.634	25.2	101.36	0.06
2021/5/29 22:20	0.634	25.2	101.35	0.05
2021/5/29 22:30	0.634	25.2	101.34	0.06
2021/5/29 22:40	0.634	25.2	101.34	0.06
2021/5/29 22:50	0.634	25.2	101.34	0.06
2021/5/29 23:00	0.634	25.2	101.32	0.06
2021/5/29 23:10	0.634	25.2	101.29	0.06
2021/5/29 23:20	0.634	25.2	101.29	0.06
2021/5/29 23:30	0.630	25.2	101.29	0.06
2021/5/29 23:40	0.630	25.2	101.31	0.06
2021/5/29 23:50	0.630	25.2	101.29	0.05
2021/5/30 0:00	0.630	25.2	101.28	0.05
2021/5/30 0:10	0.630	25.2	101.26	0.06
2021/5/30 0:20	0.627	25.2	101.25	0.05
2021/5/30 0:30	0.627	25.2	101.23	0.06
2021/5/30 0:40	0.627	25.2	101.25	0.05
2021/5/30 0:50	0.631	25.1	101.25	0.05
2021/5/30 1:00	0.628	25.1	101.25	0.05
2021/5/30 1:10	0.628	25.1	101.23	0.06
2021/5/30 1:20	0.628	25.1	101.22	0.05
2021/5/30 1:30	0.628	25.1	101.22	0.05
2021/5/30 1:40	0.628	25.1	101.22	0.05
2021/5/30 1:50	0.628	25.1	101.22	0.05
2021/5/30 2:00	0.628	25.1	101.22	0.05
2021/5/30 2:10	0.628	25.1	101.25	0.05
2021/5/30 2:20	0.628	25.1	101.25	0.05
2021/5/30 2:30	0.628	25.1	101.26	0.05
2021/5/30 2:40	0.628	25.1	101.26	0.05
2021/5/30 2:50	0.628	25.1	101.28	0.05
2021/5/30 3:00	0.631	25.1	101.29	0.05
2021/5/30 3:10	0.631	25.1	101.28	0.05
2021/5/30 3:20	0.631	25.1	101.29	0.06
2021/5/30 3:30	0.634	25.1	101.32	0.05
2021/5/30 3:40	0.634	25.1	101.34	0.05
2021/5/30 3:50	0.637	25.1	101.35	0.05
2021/5/30 4:00	0.637	25.1	101.35	0.05
2021/5/30 4:10	0.641	25.1	101.36	0.05
2021/5/30 4:20	0.644	25.0	101.35	0.05
2021/5/30 4:30	0.644	25.0	101.35	0.05
2021/5/30 4:40	0.648	25.0	101.35	0.06

2021/5/30 4:50	0.648	25.0	101.36	0.05
2021/5/30 5:00	0.648	25.0	101.36	0.05
2021/5/30 5:10	0.648	25.0	101.38	0.05
2021/5/30 5:20	0.648	25.0	101.36	0.05
2021/5/30 5:30	0.648	25.0	101.36	0.05
2021/5/30 5:40	0.648	25.0	101.38	0.05
2021/5/30 5:50	0.648	25.0	101.39	0.05
2021/5/30 6:00	0.648	25.0	101.39	0.05
2021/5/30 6:10	0.648	25.0	101.41	0.05
2021/5/30 6:20	0.644	25.0	101.41	0.05
2021/5/30 6:30	0.641	25.0	101.42	0.05
2021/5/30 6:40	0.641	25.1	101.44	0.04
2021/5/30 6:50	0.641	25.1	101.45	0.05
2021/5/30 7:00	0.637	25.2	101.45	0.05
2021/5/30 7:10	0.633	25.3	101.45	0.06
2021/5/30 7:20	0.636	25.3	101.47	0.05
2021/5/30 7:30	0.632	25.4	101.47	0.05
2021/5/30 7:40	0.635	25.4	101.47	0.05
2021/5/30 7:50	0.632	25.5	101.47	0.07
2021/5/30 8:00	0.632	25.5	101.47	0.05
2021/5/30 8:10	0.632	25.5	101.48	0.06
2021/5/30 8:20	0.628	25.6	101.50	0.07
2021/5/30 8:30	0.631	25.6	101.47	0.05
2021/5/30 8:40	0.628	25.6	101.47	0.07
2021/5/30 8:50	0.628	25.6	101.47	0.06
2021/5/30 9:00	0.631	25.6	101.47	0.06
2021/5/30 9:10	0.628	25.6	101.47	0.06
2021/5/30 9:20	0.631	25.6	101.47	0.06
2021/5/30 9:30	0.631	25.6	101.47	0.06
2021/5/30 9:40	0.631	25.6	101.45	0.05
2021/5/30 9:50	0.631	25.6	101.44	0.06
2021/5/30 10:00	0.628	25.6	101.42	0.06
2021/5/30 10:10	0.634	25.6	101.42	0.06
2021/5/30 10:20	0.634	25.6	101.42	0.05
2021/5/30 10:30	0.634	25.6	101.42	0.05
2021/5/30 10:40	0.637	25.6	101.42	0.06
2021/5/30 10:50	0.634	25.6	101.44	0.07
2021/5/30 11:00	0.633	25.7	101.45	0.06
2021/5/30 11:10	0.637	25.6	101.44	0.05
2021/5/30 11:20	0.637	25.6	101.45	0.05
2021/5/30 11:30	0.634	25.6	101.44	0.06
2021/5/30 11:40	0.631	25.6	101.44	0.06
2021/5/30 11:50	0.638	25.4	101.44	0.05
2021/5/30 12:00	0.638	25.4	101.44	0.06
2021/5/30 12:10	0.632	25.5	101.44	0.06
2021/5/30 12:20	0.632	25.5	101.44	0.05
2021/5/30 12:30	0.632	25.5	101.44	0.05
2021/5/30 12:40	0.625	25.6	101.44	0.06
2021/5/30 12:50	0.628	25.6	101.44	0.06
2021/5/30 13:00	0.628	25.6	101.44	0.06
2021/5/30 13:10	0.625	25.6	101.45	0.06
2021/5/30 13:20	0.628	25.6	101.45	0.05
2021/5/30 13:30	0.625	25.6	101.47	0.06
2021/5/30 13:40	0.624	25.7	101.47	0.06
2021/5/30 13:50	0.627	25.7	101.47	0.06
2021/5/30 14:00	0.630	25.7	101.45	0.05

2021/5/30 14:10	0.630	25.7	101.47	0.06
2021/5/30 14:20	0.631	25.6	101.47	0.05
2021/5/30 14:30	0.637	25.6	101.47	0.05
2021/5/30 14:40	0.637	25.6	101.48	0.05
2021/5/30 14:50	0.637	25.6	101.47	0.07
2021/5/30 15:00	0.637	25.6	101.45	0.04
2021/5/30 15:10	0.633	25.7	101.44	0.06
2021/5/30 15:20	0.636	25.7	101.44	0.06
2021/5/30 15:30	0.636	25.7	101.44	0.05
2021/5/30 15:40	0.639	25.7	101.42	0.05
2021/5/30 15:50	0.639	25.7	101.41	0.06
2021/5/30 16:00	0.636	25.7	101.41	0.05
2021/5/30 16:10	0.639	25.7	101.41	0.06
2021/5/30 16:20	0.642	25.7	101.41	0.05
2021/5/30 16:30	0.642	25.7	101.42	0.06
2021/5/30 16:40	0.642	25.7	101.39	0.06
2021/5/30 16:50	0.639	25.7	101.39	0.05
2021/5/30 17:00	0.639	25.7	101.39	0.06
2021/5/30 17:10	0.642	25.7	101.38	0.05
2021/5/30 17:20	0.645	25.7	101.39	0.06
2021/5/30 17:30	0.648	25.7	101.38	0.05
2021/5/30 17:40	0.642	25.7	101.39	0.05
2021/5/30 17:50	0.645	25.7	101.39	0.05
2021/5/30 18:00	0.648	25.7	101.38	0.05
2021/5/30 18:10	0.645	25.7	101.36	0.05
2021/5/30 18:20	0.645	25.7	101.38	0.07
2021/5/30 18:30	0.648	25.7	101.41	0.05
2021/5/30 18:40	0.654	25.7	101.41	0.06
2021/5/30 18:50	0.658	25.6	101.42	0.07
2021/5/30 19:00	0.658	25.6	101.44	0.07
2021/5/30 19:10	0.661	25.6	101.44	0.08
2021/5/30 19:20	0.665	25.5	101.44	0.08
2021/5/30 19:30	0.668	25.5	101.44	0.07
2021/5/30 19:40	0.668	25.5	101.44	0.07
2021/5/30 19:50	0.665	25.5	101.45	0.08
2021/5/30 20:00	0.665	25.5	101.45	0.07
2021/5/30 20:10	0.668	25.5	101.45	0.07
2021/5/30 20:20	0.668	25.5	101.47	0.07
2021/5/30 20:30	0.671	25.5	101.48	0.07
2021/5/30 20:40	0.668	25.5	101.50	0.08
2021/5/30 20:50	0.668	25.5	101.51	0.08
2021/5/30 21:00	0.672	25.4	101.53	0.07
2021/5/30 21:10	0.675	25.4	101.53	0.07
2021/5/30 21:20	0.675	25.4	101.53	0.07
2021/5/30 21:30	0.679	25.4	101.54	0.07
2021/5/30 21:40	0.679	25.4	101.54	0.07
2021/5/30 21:50	0.679	25.4	101.54	0.07
2021/5/30 22:00	0.679	25.4	101.54	0.07
2021/5/30 22:10	0.682	25.4	101.54	0.07
2021/5/30 22:20	0.682	25.4	101.53	0.07
2021/5/30 22:30	0.682	25.4	101.53	0.08
2021/5/30 22:40	0.685	25.4	101.53	0.07
2021/5/30 22:50	0.685	25.4	101.53	0.07
2021/5/30 23:00	0.685	25.4	101.53	0.07
2021/5/30 23:10	0.685	25.4	101.51	0.08
2021/5/30 23:20	0.682	25.4	101.51	0.08

2021/5/30 23:30	0.685	25.4	101.50	0.07
2021/5/30 23:40	0.685	25.4	101.50	0.07
2021/5/30 23:50	0.685	25.4	101.50	0.08
2021/5/31 0:00	0.685	25.4	101.48	0.07
2021/5/31 0:10	0.685	25.4	101.48	0.08
2021/5/31 0:20	0.685	25.4	101.47	0.08
2021/5/31 0:30	0.685	25.4	101.45	0.08
2021/5/31 0:40	0.685	25.4	101.45	0.07
2021/5/31 0:50	0.685	25.4	101.45	0.08
2021/5/31 1:00	0.685	25.4	101.45	0.08
2021/5/31 1:10	0.685	25.4	101.45	0.07
2021/5/31 1:20	0.685	25.4	101.44	0.07
2021/5/31 1:30	0.682	25.4	101.45	0.08
2021/5/31 1:40	0.682	25.4	101.45	0.08
2021/5/31 1:50	0.679	25.4	101.45	0.07
2021/5/31 2:00	0.683	25.3	101.45	0.08
2021/5/31 2:10	0.679	25.3	101.44	0.08
2021/5/31 2:20	0.679	25.3	101.44	0.08
2021/5/31 2:30	0.676	25.3	101.42	0.07
2021/5/31 2:40	0.676	25.3	101.41	0.07
2021/5/31 2:50	0.673	25.3	101.39	0.07
2021/5/31 3:00	0.670	25.3	101.39	0.07
2021/5/31 3:10	0.671	25.2	101.41	0.07
2021/5/31 3:20	0.668	25.2	101.41	0.07
2021/5/31 3:30	0.668	25.2	101.41	0.07
2021/5/31 3:40	0.665	25.2	101.41	0.07
2021/5/31 3:50	0.665	25.2	101.42	0.07
2021/5/31 4:00	0.662	25.2	101.44	0.07
2021/5/31 4:10	0.662	25.2	101.44	0.07
2021/5/31 4:20	0.659	25.2	101.45	0.07
2021/5/31 4:30	0.659	25.2	101.45	0.07
2021/5/31 4:40	0.659	25.2	101.48	0.07
2021/5/31 4:50	0.655	25.2	101.50	0.07
2021/5/31 5:00	0.655	25.2	101.50	0.06
2021/5/31 5:10	0.656	25.1	101.51	0.07
2021/5/31 5:20	0.653	25.1	101.51	0.06
2021/5/31 5:30	0.653	25.1	101.53	0.07
2021/5/31 5:40	0.650	25.1	101.53	0.06
2021/5/31 5:50	0.650	25.1	101.54	0.06
2021/5/31 6:00	0.647	25.1	101.54	0.06
2021/5/31 6:10	0.647	25.1	101.57	0.06
2021/5/31 6:20	0.648	25.0	101.57	0.07
2021/5/31 6:30	0.641	25.1	101.58	0.06
2021/5/31 6:40	0.641	25.1	101.58	0.05
2021/5/31 6:50	0.634	25.2	101.58	0.05
2021/5/31 7:00	0.634	25.2	101.60	0.05
2021/5/31 7:10	0.633	25.3	101.61	0.06
2021/5/31 7:20	0.629	25.4	101.63	0.06
2021/5/31 7:30	0.629	25.4	101.63	0.08
2021/5/31 7:40	0.625	25.5	101.64	0.06
2021/5/31 7:50	0.625	25.5	101.64	0.06
2021/5/31 8:00	0.625	25.5	101.64	0.04
2021/5/31 8:10	0.630	25.3	101.63	0.06
2021/5/31 8:20	0.630	25.3	101.63	0.06
2021/5/31 8:30	0.630	25.3	101.63	0.06
2021/5/31 8:40	0.626	25.4	101.63	0.07

2021/5/31 8:50	0.626	25.4	101.63	0.06
2021/5/31 9:00	0.625	25.5	101.63	0.06
2021/5/31 9:10	0.625	25.5	101.63	0.08
2021/5/31 9:20	0.622	25.5	101.64	0.05
2021/5/31 9:30	0.622	25.6	101.64	0.07
2021/5/31 9:40	0.622	25.6	101.64	0.06
2021/5/31 9:50	0.625	25.6	101.66	0.06
2021/5/31 10:00	0.628	25.6	101.66	0.06
2021/5/31 10:10	0.631	25.6	101.64	0.05
2021/5/31 10:20	0.634	25.6	101.64	0.07
2021/5/31 10:30	0.637	25.6	101.64	0.05
2021/5/31 10:40	0.637	25.6	101.64	0.07
2021/5/31 10:50	0.640	25.6	101.64	0.05
2021/5/31 11:00	0.639	25.7	101.63	0.07
2021/5/31 11:10	0.639	25.7	101.63	0.06
2021/5/31 11:20	0.642	25.7	101.61	0.06
2021/5/31 11:30	0.642	25.7	101.61	0.05
2021/5/31 11:40	0.642	25.7	101.61	0.05
2021/5/31 11:50	0.645	25.7	101.60	0.05
2021/5/31 12:00	0.642	25.7	101.60	0.06
2021/5/31 12:10	0.648	25.7	101.60	0.05
2021/5/31 12:20	0.645	25.7	101.60	0.07
2021/5/31 12:30	0.651	25.7	101.58	0.05
2021/5/31 12:40	0.648	25.7	101.58	0.06
2021/5/31 12:50	0.654	25.7	101.57	0.06
2021/5/31 13:00	0.658	25.6	101.55	0.06
2021/5/31 13:10	0.655	25.6	101.54	0.07
2021/5/31 13:20	0.661	25.6	101.54	0.05
2021/5/31 13:30	0.654	25.7	101.54	0.05
2021/5/31 13:40	0.661	25.6	101.54	0.06
2021/5/31 13:50	0.657	25.7	101.54	0.05
2021/5/31 14:00	0.657	25.7	101.53	0.05
2021/5/31 14:10	0.661	25.7	101.53	0.05
2021/5/31 14:20	0.657	25.7	101.53	0.07
2021/5/31 14:30	0.661	25.7	101.53	0.05
2021/5/31 14:40	0.661	25.7	101.51	0.06
2021/5/31 14:50	0.661	25.7	101.50	0.05
2021/5/31 15:00	0.657	25.8	101.50	0.05
2021/5/31 15:10	0.664	25.7	101.50	0.06
2021/5/31 15:20	0.667	25.7	101.48	0.05
2021/5/31 15:30	0.664	25.7	101.48	0.06
2021/5/31 15:40	0.667	25.7	101.48	0.05
2021/5/31 15:50	0.667	25.7	101.47	0.06
2021/5/31 16:00	0.670	25.7	101.45	0.05
2021/5/31 16:10	0.666	25.8	101.45	0.06
2021/5/31 16:20	0.666	25.8	101.44	0.05
2021/5/31 16:30	0.666	25.8	101.44	0.05
2021/5/31 16:40	0.673	25.7	101.44	0.06
2021/5/31 16:50	0.673	25.7	101.44	0.05
2021/5/31 17:00	0.670	25.7	101.42	0.06
2021/5/31 17:10	0.670	25.7	101.42	0.05
2021/5/31 17:20	0.654	25.7	101.41	0.06
2021/5/31 17:30	0.671	25.5	101.41	0.08
2021/5/31 17:40	0.675	25.5	101.39	0.06
2021/5/31 17:50	0.678	25.5	101.39	0.05
2021/5/31 18:00	0.671	25.6	101.38	0.07

2021/5/31 18:10	0.671	25.6	101.38	0.06
2021/5/31 18:20	0.670	25.7	101.38	0.06
2021/5/31 18:30	0.677	25.6	101.41	0.07
2021/5/31 18:40	0.680	25.6	101.41	0.07
2021/5/31 18:50	0.680	25.6	101.41	0.07
2021/5/31 19:00	0.677	25.6	101.41	0.08
2021/5/31 19:10	0.678	25.5	101.42	0.08
2021/5/31 19:20	0.678	25.5	101.42	0.07
2021/5/31 19:30	0.678	25.5	101.44	0.08
2021/5/31 19:40	0.678	25.5	101.44	0.08
2021/5/31 19:50	0.678	25.5	101.44	0.07
2021/5/31 20:00	0.678	25.5	101.44	0.08
2021/5/31 20:10	0.678	25.5	101.44	0.08
2021/5/31 20:20	0.675	25.5	101.45	0.08
2021/5/31 20:30	0.675	25.5	101.45	0.08
2021/5/31 20:40	0.675	25.5	101.45	0.08
2021/5/31 20:50	0.679	25.4	101.47	0.08
2021/5/31 21:00	0.682	25.4	101.47	0.08
2021/5/31 21:10	0.682	25.4	101.47	0.08
2021/5/31 21:20	0.679	25.4	101.48	0.08
2021/5/31 21:30	0.679	25.4	101.48	0.08
2021/5/31 21:40	0.679	25.4	101.48	0.08
2021/5/31 21:50	0.679	25.4	101.50	0.08
2021/5/31 22:00	0.679	25.4	101.51	0.08
2021/5/31 22:10	0.679	25.4	101.51	0.08
2021/5/31 22:20	0.679	25.4	101.51	0.08
2021/5/31 22:30	0.679	25.4	101.51	0.08
2021/5/31 22:40	0.679	25.4	101.51	0.08
2021/5/31 22:50	0.679	25.4	101.51	0.08
2021/5/31 23:00	0.679	25.4	101.53	0.08
2021/5/31 23:10	0.679	25.4	101.53	0.08
2021/5/31 23:20	0.679	25.4	101.53	0.08
2021/5/31 23:30	0.675	25.4	101.53	0.08
2021/5/31 23:40	0.675	25.4	101.53	0.08
2021/5/31 23:50	0.675	25.4	101.53	0.08
2021/6/1 0:00	0.675	25.4	101.53	0.08
