# Supplementary material for: Effect of Nutrient Solution Flow Rate on Hydroponic Plant Growth and Root Morphology
Source: Plants (Basel). 2021 Sep 5;10(9):1840. doi: 10.3390/plants10091840 (PMC8465728; doi:10.3390/plants10091840)
Supplement: Supplementary file 1 [file plants-10-01840-s001.zip › plants-1359110-supplementary/Table S2. Detail data of leaf area, fresh weight, and dry weight under different flow rates in this study..pdf]

Table S2. Detail data of leaf area, fresh weight, and dry weight under different flow rates in this study.

Flow rate (L/min)	Number	Leaf area (cm2/plant)	Fresh weight (shoots)(g/plant)	Fresh weight (roots)(g/plant)	Dry weight (shoots)(g/plant)	Dry weight (roots)(g/plant)
2	2-1	815.23	63.13	9.24	6.07	0.83
	2-2	889.09	77.43	11.38	7.44	0.95
	2-3	815.21	75.39	13.81	7.07	1.15
	2-4	853.81	83.84	14.15	5.95	1.12
4	4-1	1207.79	121.00	19.20	6.98	1.11
	4-2	935.41	99.91	14.50	6.06	1.06
	4-3	943.74	80.89	12.55	7.66	1.04
	4-4	928.87	77.28	13.55	7.25	1.05
6	6-1	786.90	59.89	8.57	5.82	0.94
	6-2	681.42	55.56	9.37	6.41	1.07
	6-3	650.97	47.72	8.59	6.06	1.04
	6-4	680.81	48.94	9.01	6.45	1.17
8	8-1	549.09	38.66	7.17	5.09	0.83
	8-2	664.64	31.52	6.87	5.03	0.85
	8-3	566.00	38.65	6.12	5.02	0.74
	8-4	531.00	46.05	8.00	4.44	0.82
