# Supplementary material for: Effect of Nutrient Solution Flow Rate on Hydroponic Plant Growth and Root Morphology
Source: Plants (Basel). 2021 Sep 5;10(9):1840. doi: 10.3390/plants10091840 (PMC8465728; doi:10.3390/plants10091840)
Supplement: Supplementary file 1 [file plants-10-01840-s001.zip › plants-1359110-supplementary/Table S3. Detail data of root morphology and N contents of plants under different flow rates in this study..pdf]

Table S3. Detail data of root morphology and N contents of plants under different flow rates in this study.

Flow rate (L/min)	Number	Root length (cm/plant)	Root surface area (cm2/plant)	Root volume (cm3/plant)	N contents (shoots)(mg/g dw)	N contents (roots)(mg/g dw)
2	2-1	4299.22	694.83	9.36	54.19	43.48
	2-2	5566.07	918.46	12.70	49.51	50.01
	2-3	5925.07	989.04	14.21	46.52	42.35
	2-4	6160.09	1032.86	13.99	52.51	48.64
4	4-1	6663.06	1468.58	27.53	55.60	44.21
	4-2	5983.97	1118.30	16.70	55.30	44.08
	4-3	5391.11	933.27	13.07	50.33	43.80
	4-4	3945.64	867.68	17.61	47.58	42.53
6	6-1	4749.08	656.26	7.26	45.43	41.53
	6-2	5000.74	688.33	7.55	47.20	40.92
	6-3	4348.74	585.97	6.30	51.06	40.43
	6-4	4181.59	589.53	6.63	48.11	40.90
8	8-1	2577.49	379.55	4.45	43.65	40.29
	8-2	2370.77	334.86	3.78	40.32	36.74
	8-3	1889.31	278.93	3.35	43.69	35.09
	8-4	3252.92	541.35	7.42	47.40	37.59
