# Supplementary material for: Effect of Nutrient Solution Flow Rate on Hydroponic Plant Growth and Root Morphology
Source: Plants (Basel). 2021 Sep 5;10(9):1840. doi: 10.3390/plants10091840 (PMC8465728; doi:10.3390/plants10091840)
Supplement: Supplementary file 1 [file plants-10-01840-s001.zip › plants-1359110-supplementary/Table S4. Detail data of Hemicellulose I, II, and Cellulose contents of roots under different flow rates in this study..pdf]

Table S4. Detail data of Hemicellulose I, II, and Cellulose contents of roots under different flow rates in this study.

Flow rate (L/min)	Number	Hemiellulose I contents per unit dry weight of root (mg/g)	Hemiellulose II contents per unit dry weight of root (mg/g)	Cellulose contents per unit dry weight of root (mg/g)
2	2-1	373.71	424.68	159.82
	2-2	265.18	369.63	104.02
	2-3	238.32	420.89	182.35
	2-4	297.82	417.23	225.04
4	4-1	308.81	485.79	154.02
	4-2	319.05	502.44	155.53
	4-3	375.19	441.48	105.02
	4-4	287.12	452.69	111.32
6	6-1	279.32	417.88	87.97
	6-2	294.00	387.45	126.89
	6-3	275.83	360.11	71.71
	6-4	247.54	355.49	133.31
8	8-1	335.11	353.16	256.11
	8-2	239.15	313.84	124.48
	8-3	246.95	328.84	172.41
	8-4	274.34	368.29	172.23
